# Supplementary material for: A Computational Approach to Explore the Interaction of Semisynthetic Nitrogenous Heterocyclic Compounds with the SARS-CoV-2 Main Protease
Source: Biomolecules. 2020 Dec 27;11(1):18. doi: 10.3390/biom11010018 (PMC7824519; doi:10.3390/biom11010018)
Supplement: Supplementary file 1 [file biomolecules-11-00018-s001.zip › Figure S1.pdf]

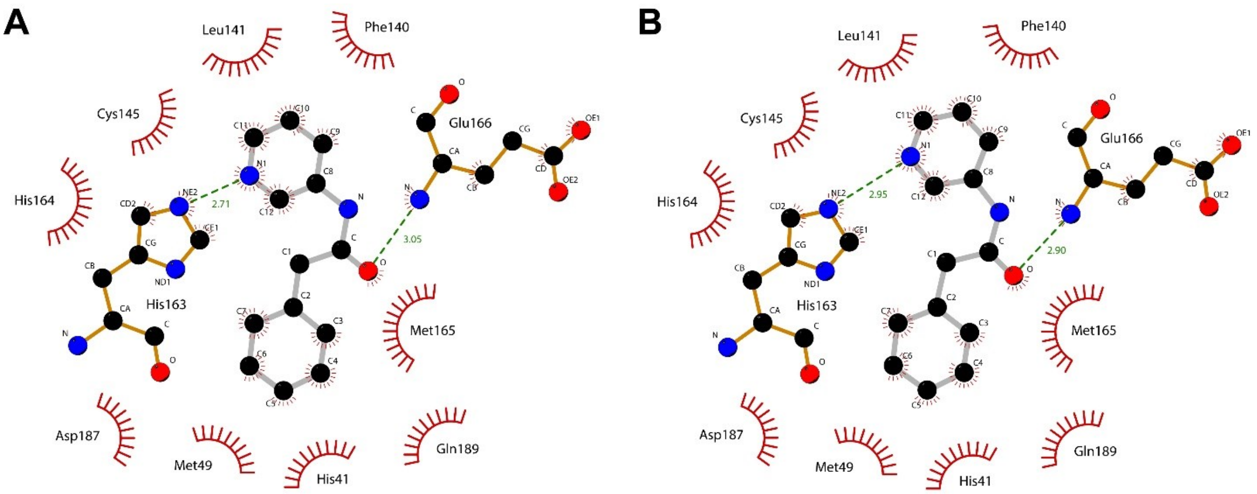

Figure S1. Non-covalent interactions among the GWS ligand and amino acid residues from the active site of SARS-CoV-2 M^{pro}. **(A)** Interactions of the co-crystalized ligand. **(B)** Interactions after redocking with Smina and the flexible docking approach **(B)**. Hydrogen bonds are represented by dashed green lines together with the estimated length in Angstroms (Å). Other non-covalent interactions are represented with red arches.
