# Supplementary material for: A Computational Approach to Explore the Interaction of Semisynthetic Nitrogenous Heterocyclic Compounds with the SARS-CoV-2 Main Protease
Source: Biomolecules. 2020 Dec 27;11(1):18. doi: 10.3390/biom11010018 (PMC7824519; doi:10.3390/biom11010018)
Supplement: Supplementary file 1 [file biomolecules-11-00018-s001.zip › Table S3.pdf]

Table S3. Docking energy scores and lowest-energy poses for the compounds considered in this study.

Compound	Energy scores (kcal/mol)		Pose comparison<br>Blue = DOCK, Green = Smina	
	DOCK GBSA	Smina	Non-covalent interactions	Surface<br>Red wire = N3 orientation
**PQ**	-25.8	-8.5	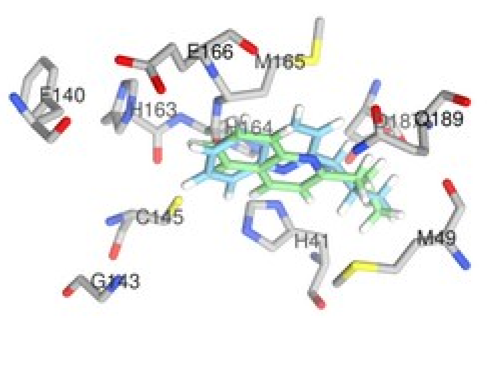	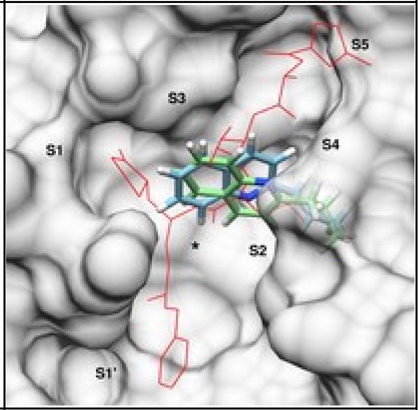
**1**	-25.6	-8.3	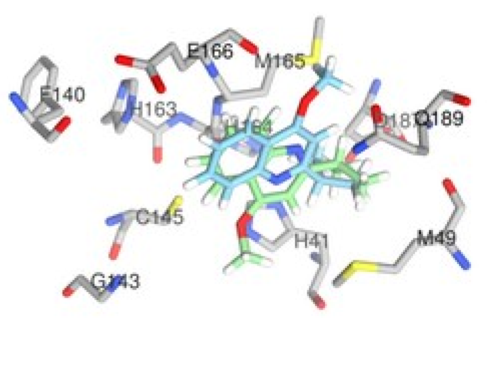	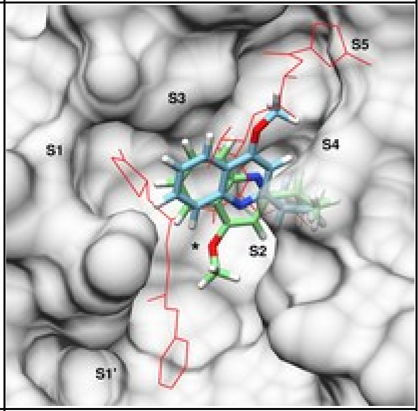
**2**	-25.3	-7.8	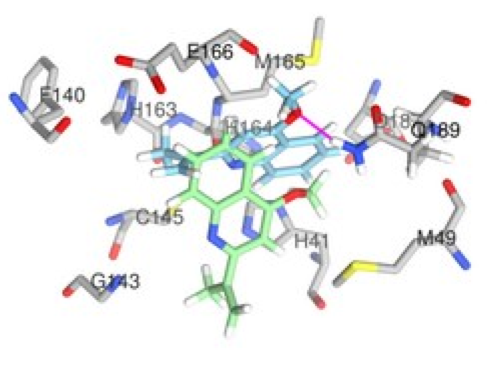	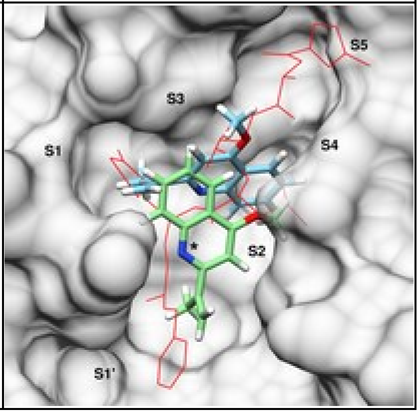

**3**	-24.9	-9.0	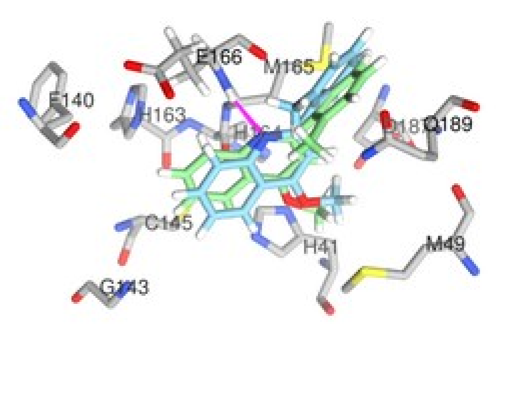	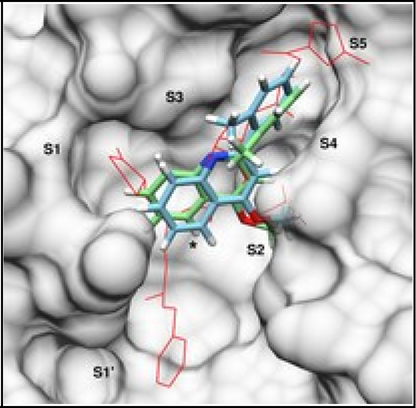
**4**	-28.2	-10.2	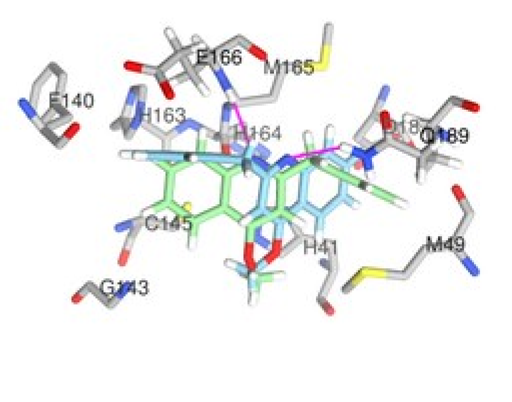	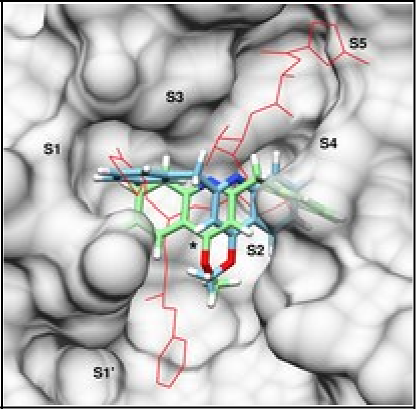
**5**	-27.6	-10.0	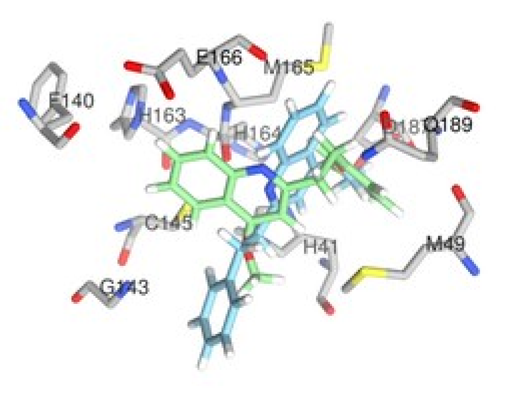	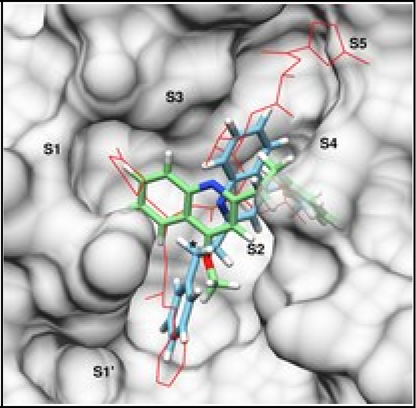
**6**	-30.1	-10.7	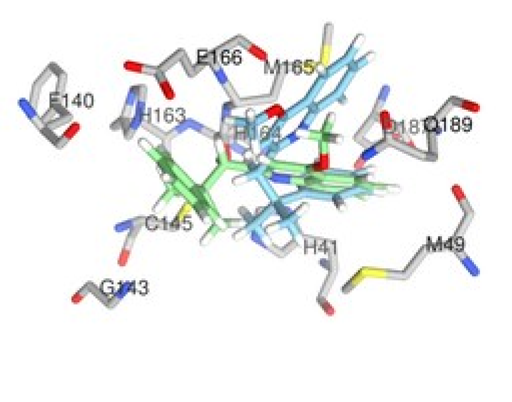	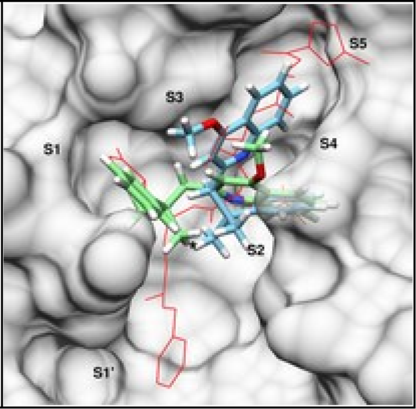

**7**	-25.5	-9.0	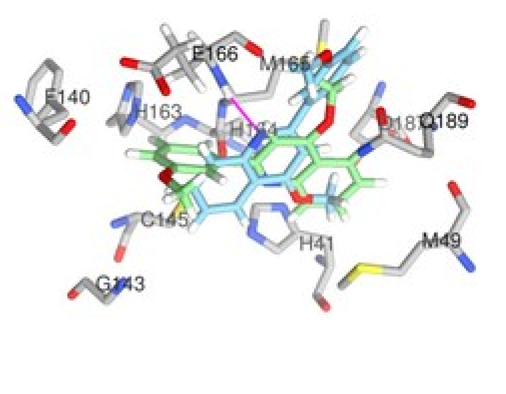	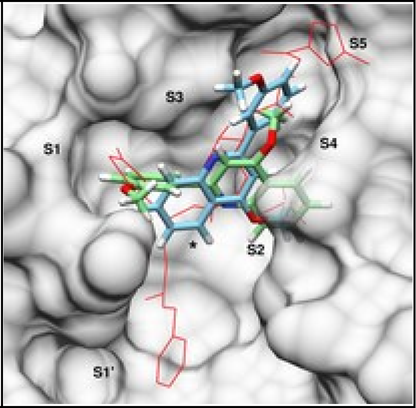
**8**	-28.0	-8.5	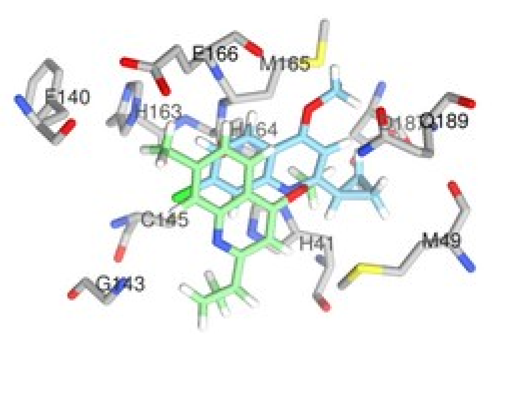	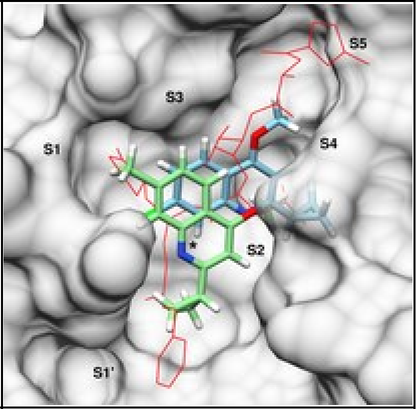
**9**	-29.7	-9.8	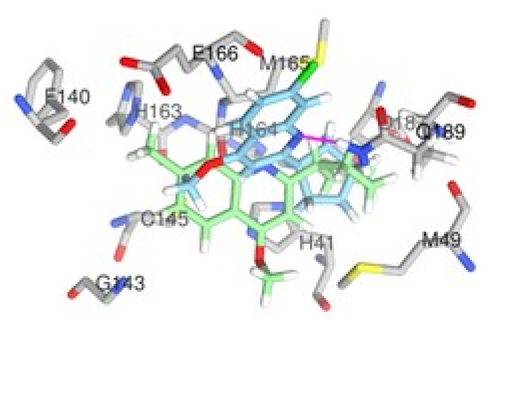	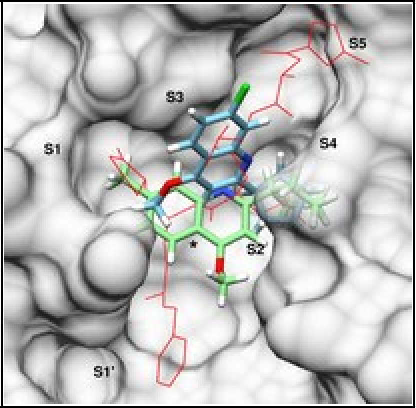
**10**	-21.2	-8.1	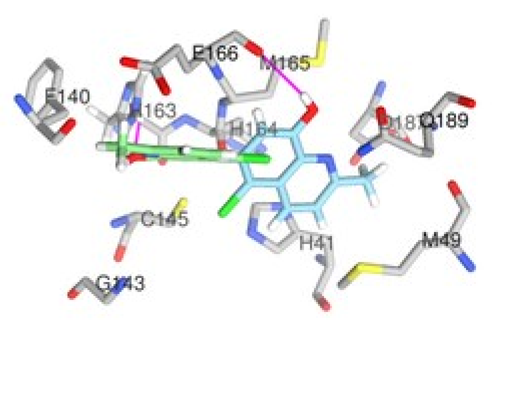	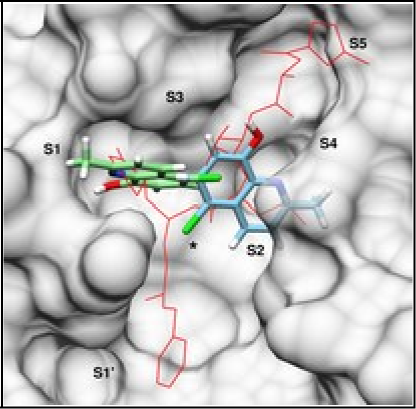

<table>
<thead>
<tr><th></th><th></th><th></th><th></th><th></th></tr>
</thead>
<tbody>
<tr>
<td>11</td>
<td>-24.2</td>
<td>-8.5</td>
<td>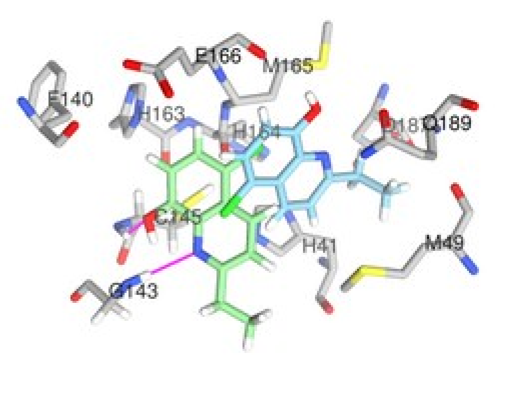
</td>
<td>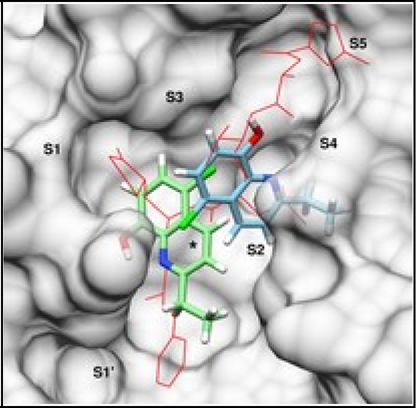
</td>
</tr>
<tr>
<td>12</td>
<td>-25.3</td>
<td>-8.7</td>
<td>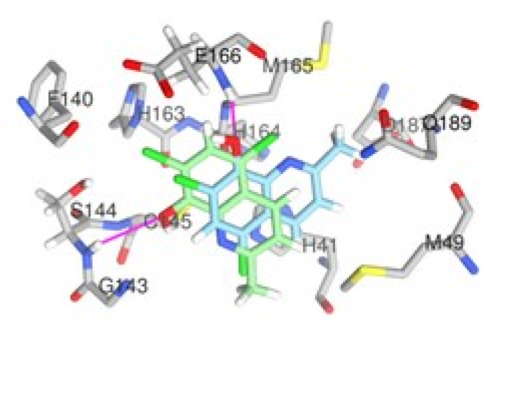
</td>
<td>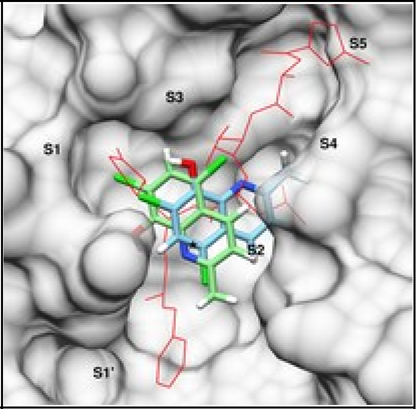
</td>
</tr>
<tr>
<td>13</td>
<td>-28.7</td>
<td>-9.6</td>
<td>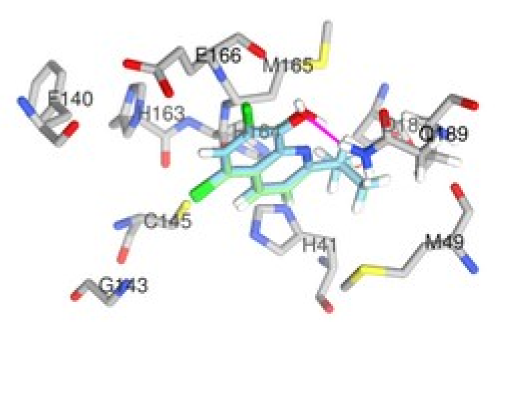
</td>
<td>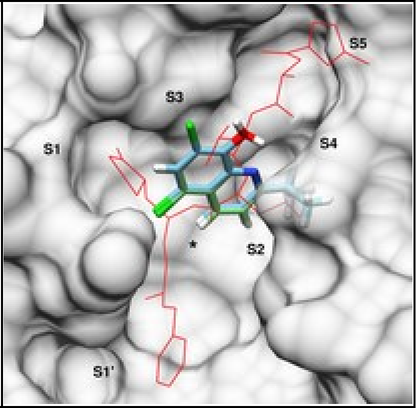
</td>
</tr>
<tr>
<td>14</td>
<td>-29.1</td>
<td>-8.5</td>
<td>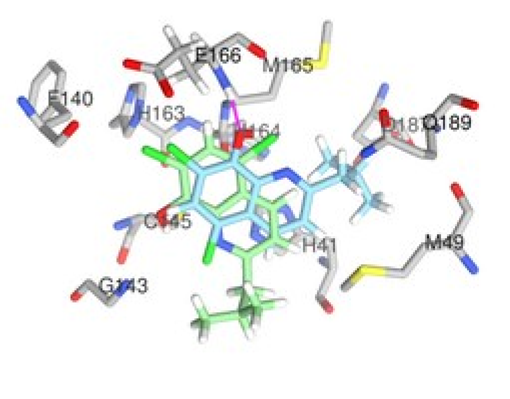
</td>
<td>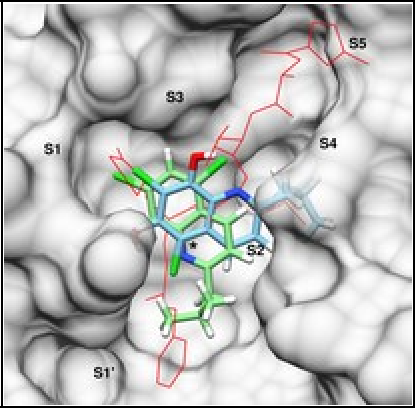
</td>
</tr>
</tbody>
</table>

**15**	-31.4	-9.5	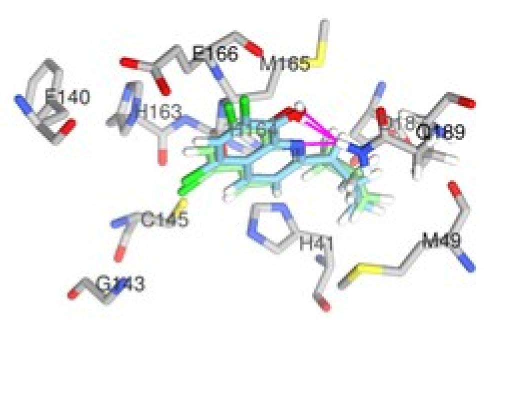	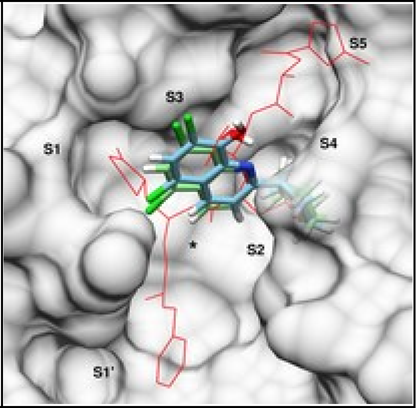
**16**	-28.1	-9.3	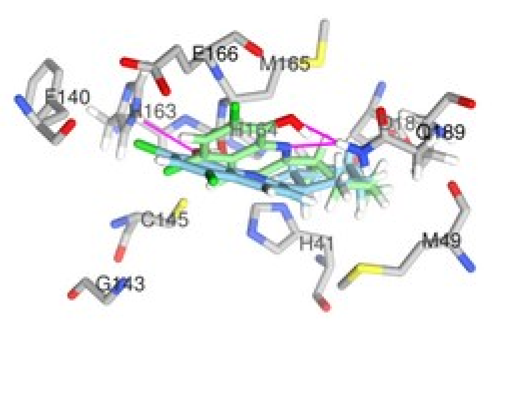	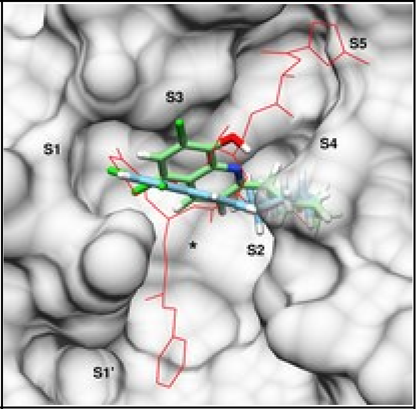
**17**	-29.1	-11.4	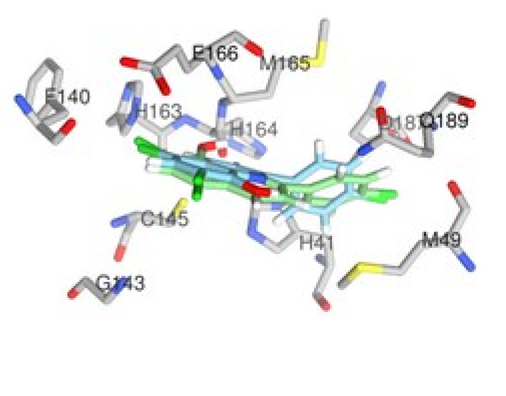	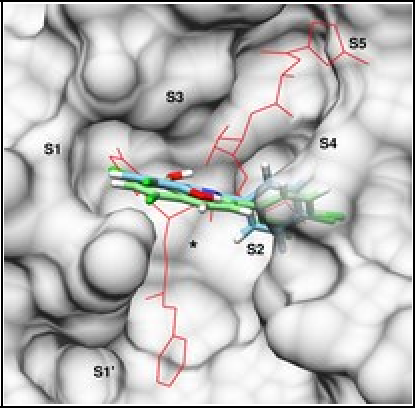
**18**	-32.3	-8.7	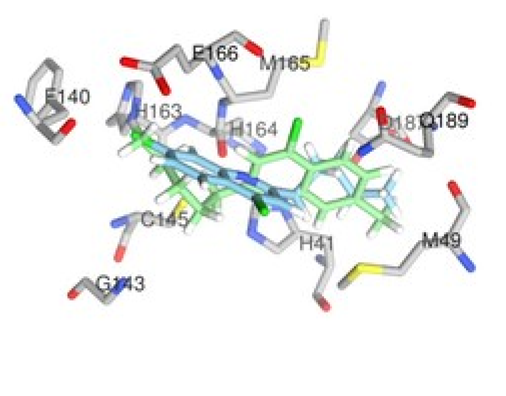	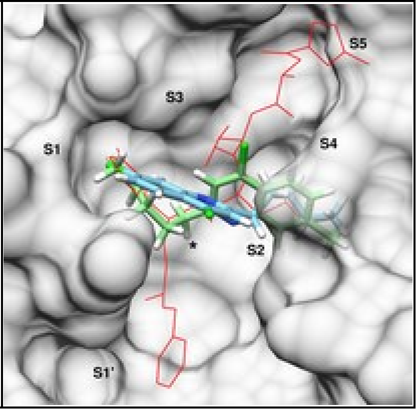

<table>
<tr><td>19</td><td>-29.0</td><td>-8.8</td><td>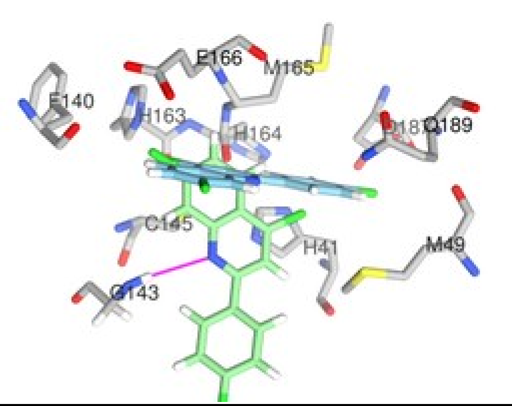
</td><td>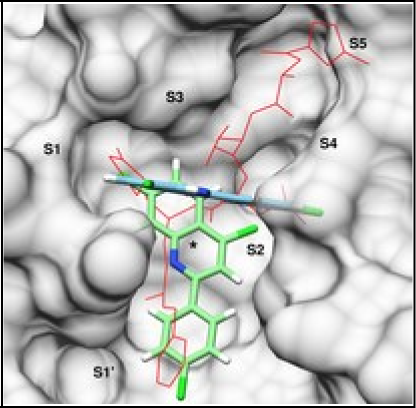
</td></tr>
<tr><td>20</td><td>-33.0</td><td>-10.3</td><td>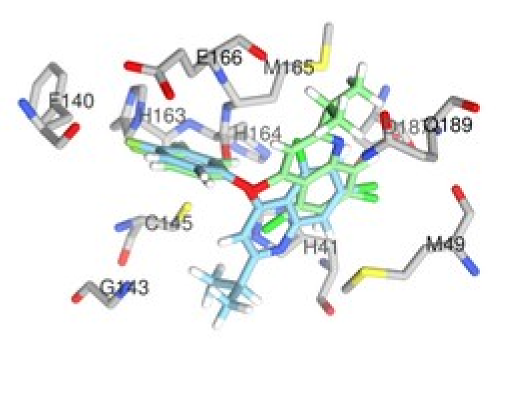
</td><td>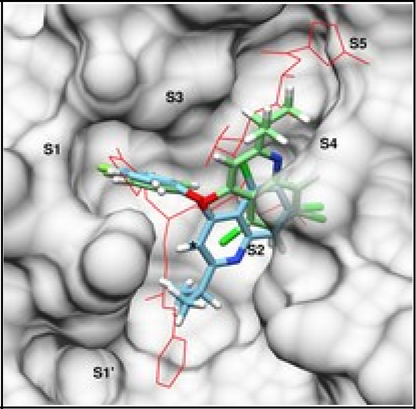
</td></tr>
<tr><td>21</td><td>-32.4</td><td>-12.5</td><td>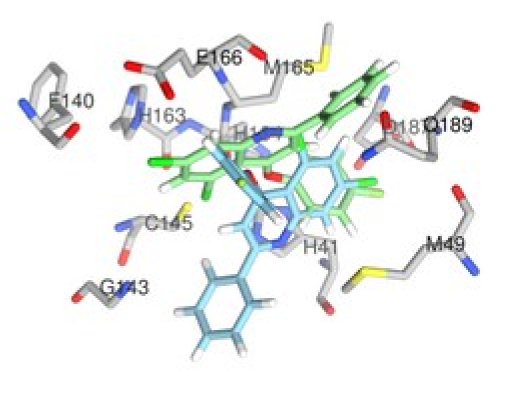
</td><td>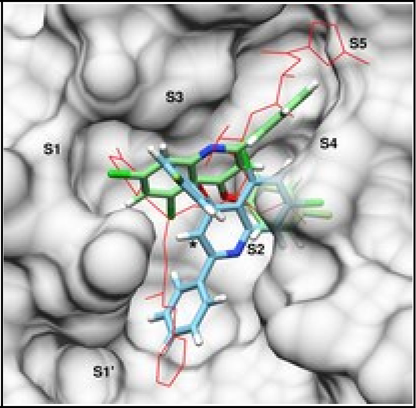
</td></tr>
<tr><td>22_R</td><td>-38.5</td><td>-10.0</td><td>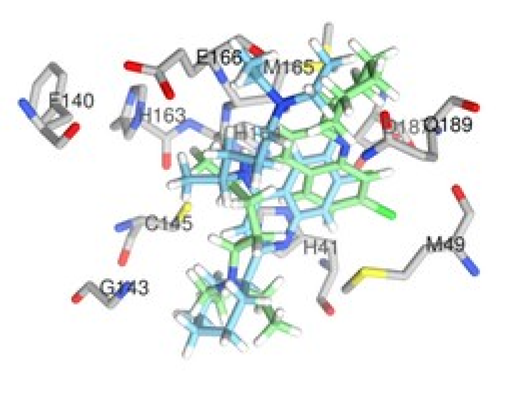
</td><td>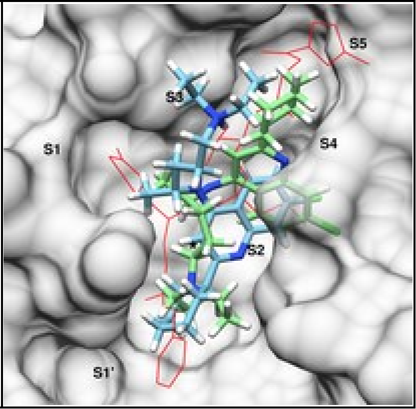
</td></tr>
</table>

**22_S**	-39.2	-9.8	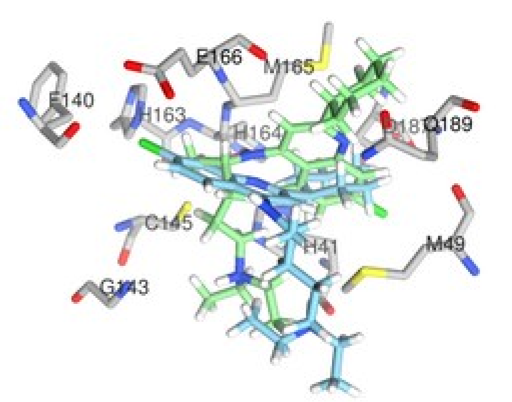	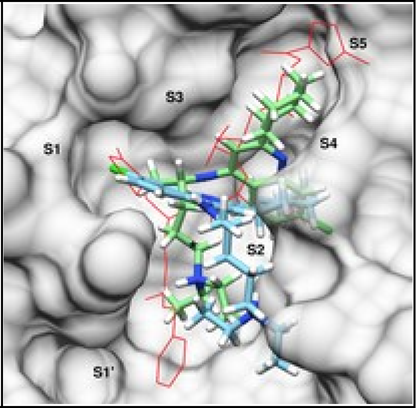
**23**	-37.1	-11.1	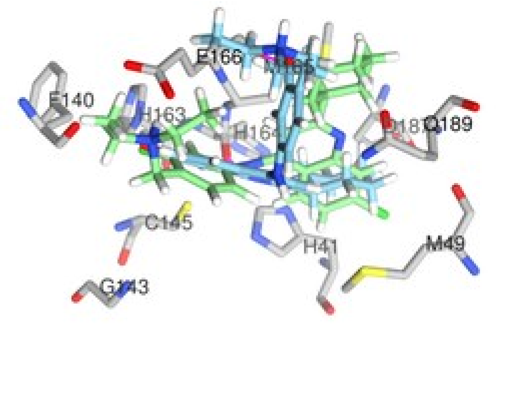	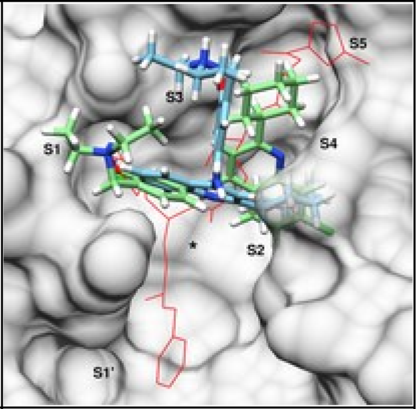
**24**	-36.2	-11.9	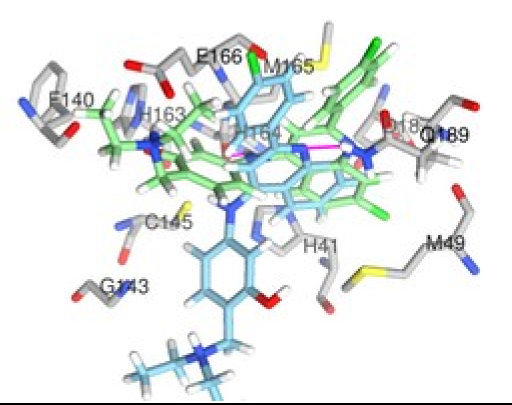	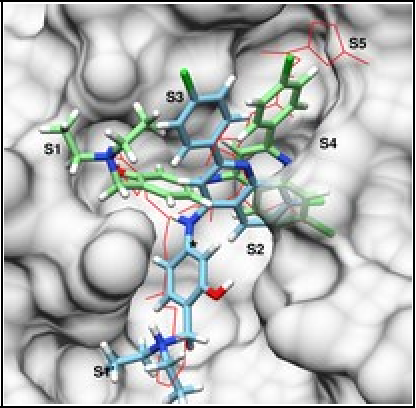
**25**	-35.9	-11.2	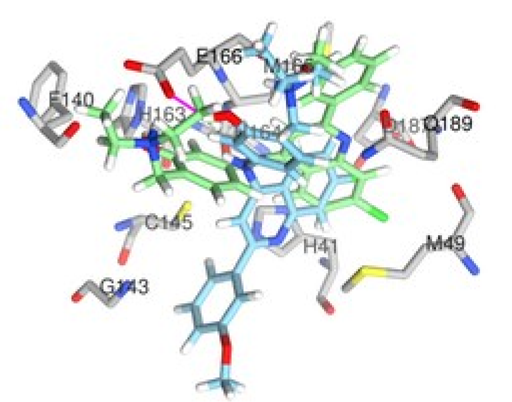	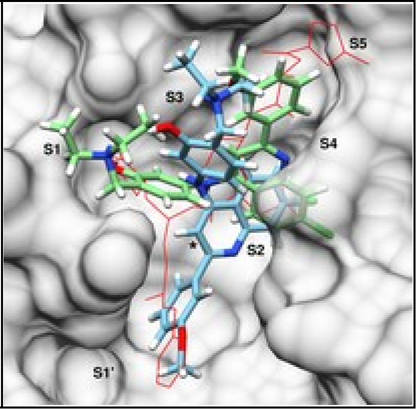

**26**	-21.5	-8.6	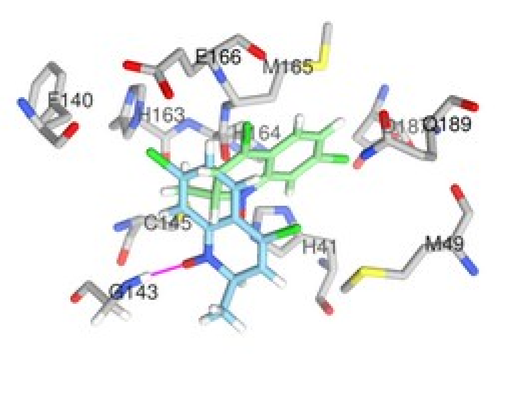	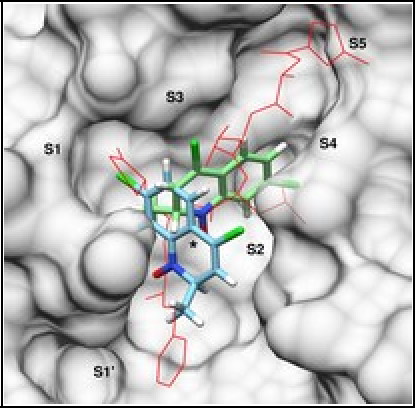
**27**	-32.2	-10.4	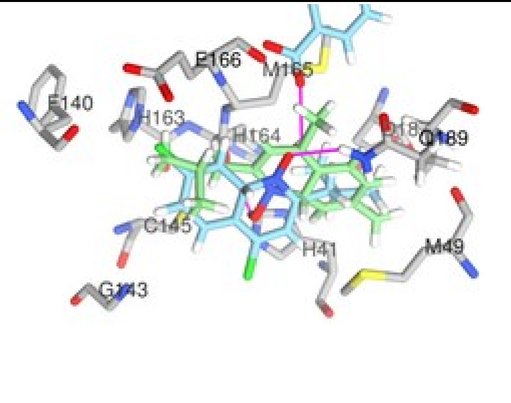	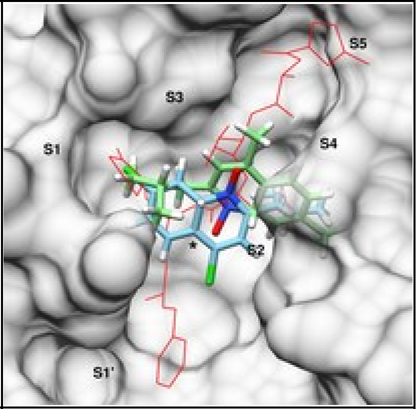
**28**	-25.6	-9.9	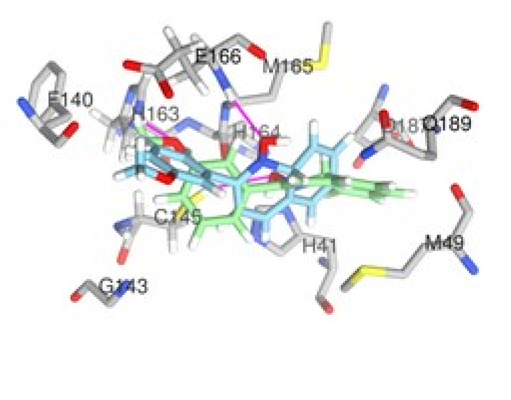	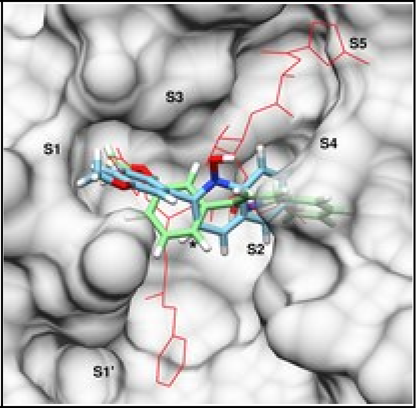
**29**	-28.8	-8.3	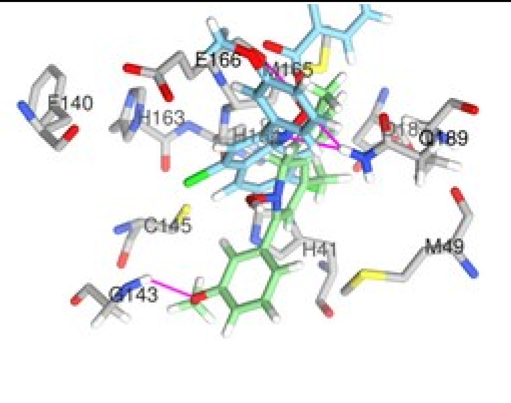	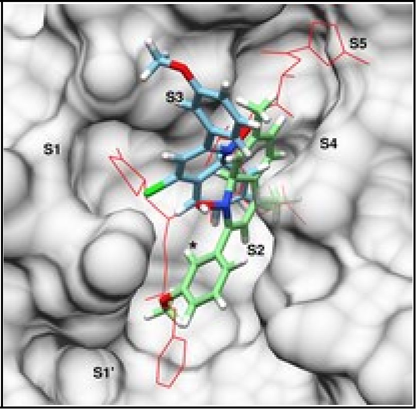

**30**	-29.3	-11.1	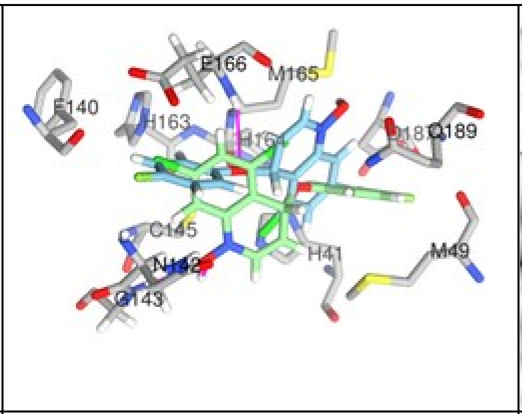	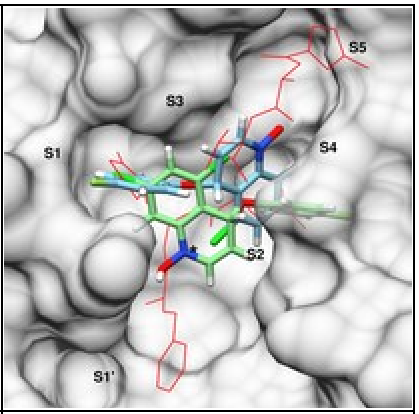
**31**	-22.9	-9.7	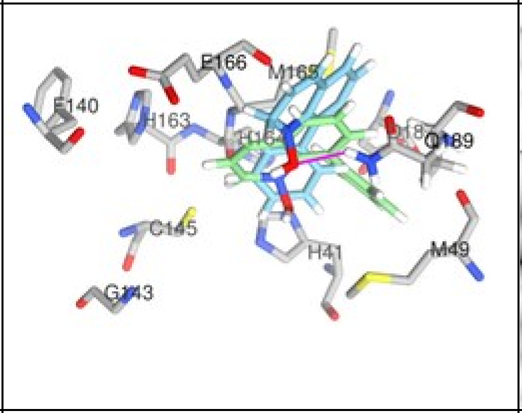	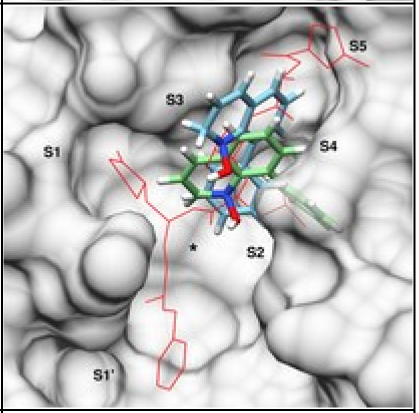
**32**	-22.3	-8.7	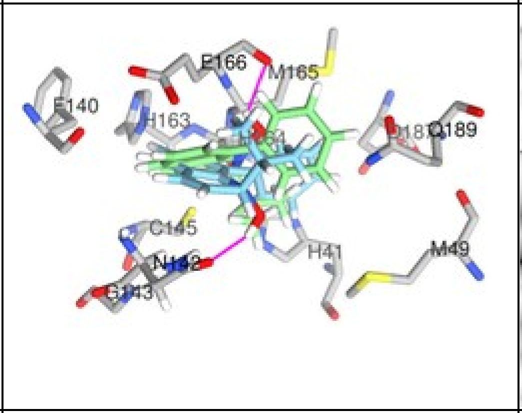	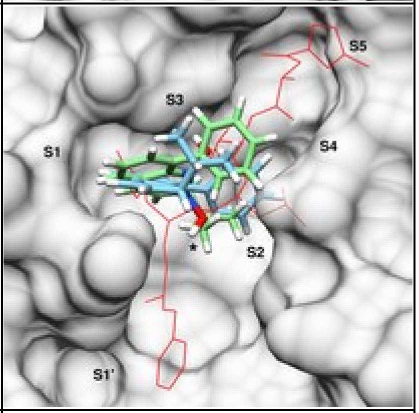
**33**	-28.1	-9.4	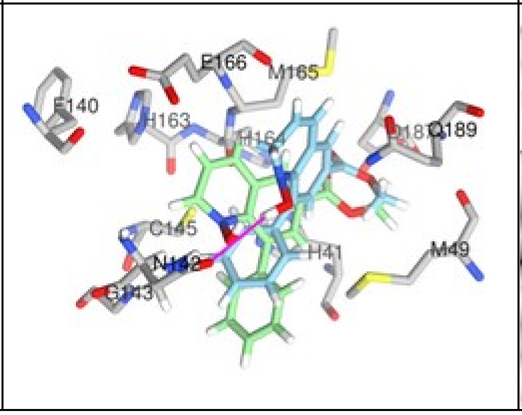	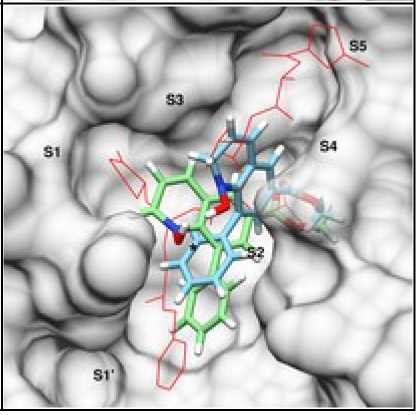

**34**	-29.4	-10.1	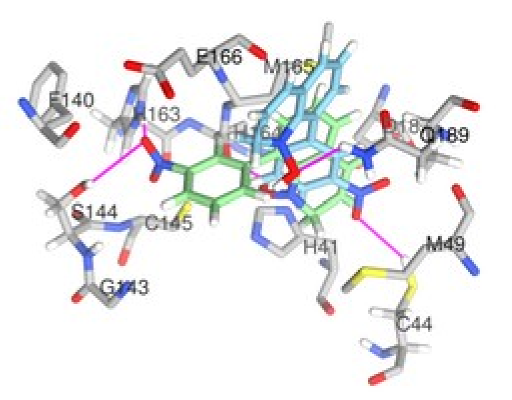	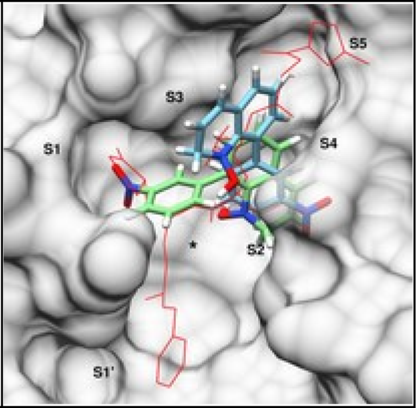
**35**	-27.3	-11.1	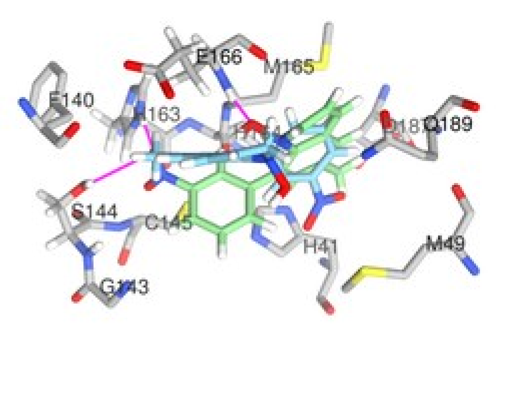	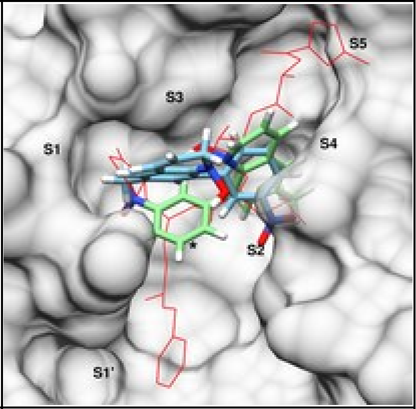
**36**	-30.6	-9.1	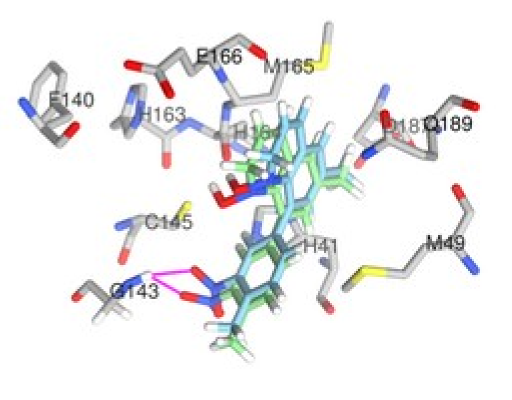	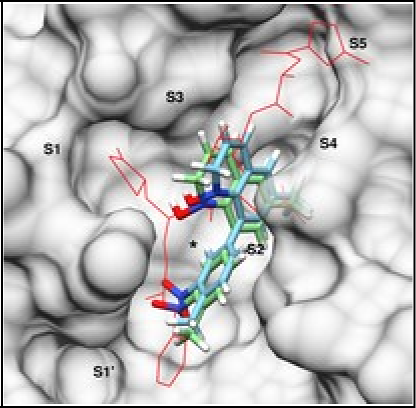
**37**	-22.6	-10.5	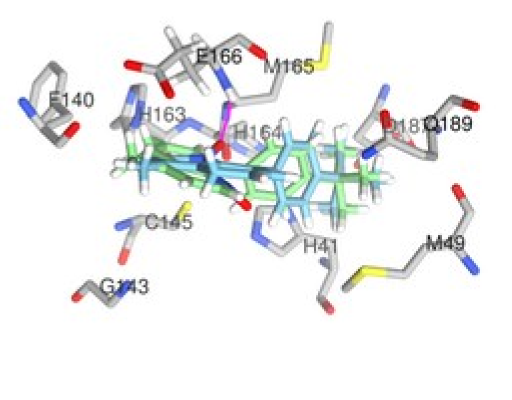	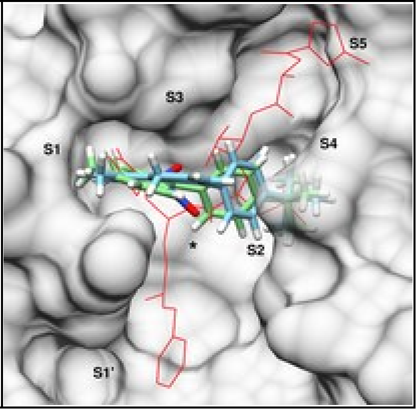

**38**	-23.0	-8.3	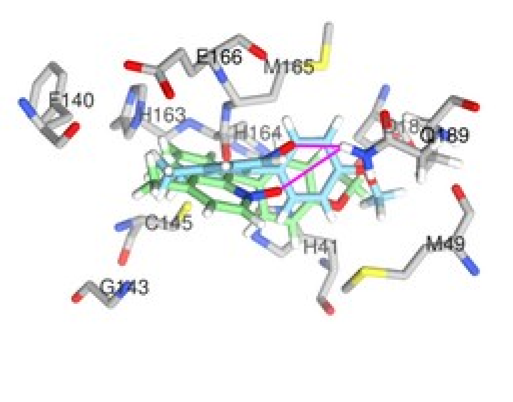	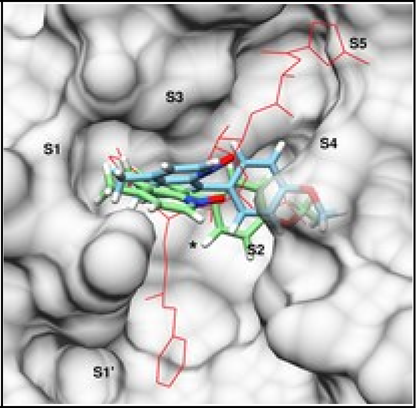
**39**	-28.5	-9.3	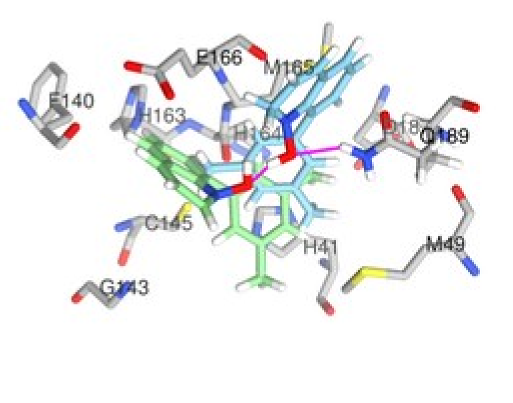	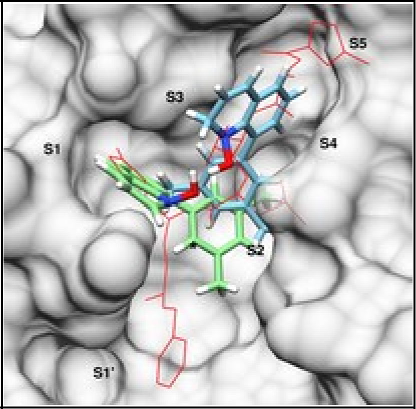
**40**	-30.0	-10.0	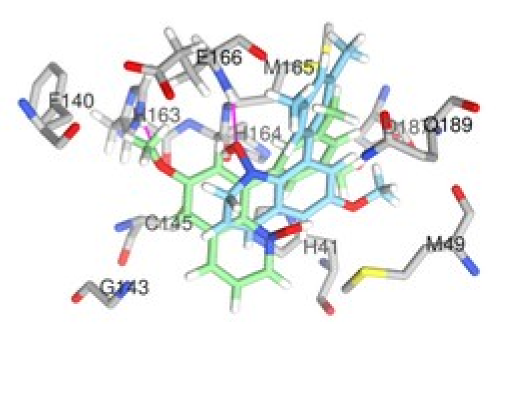	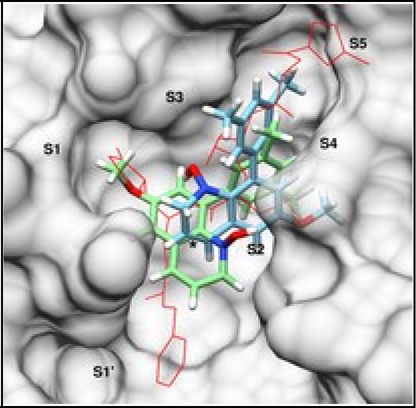
**41**	-27.5	-9.0	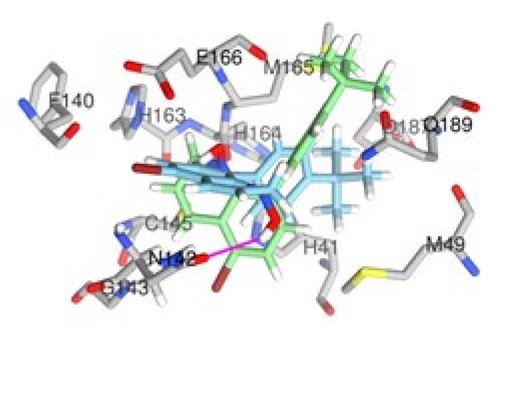	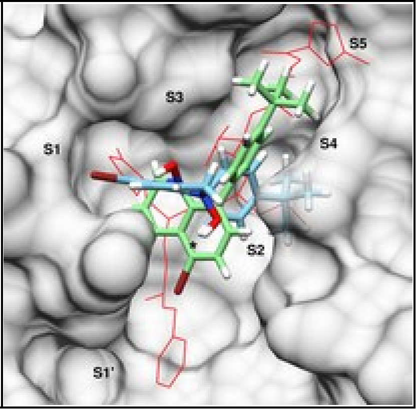

**42**	-29.2	-9.3	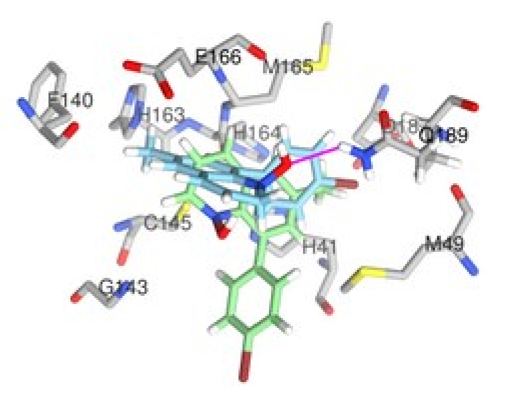	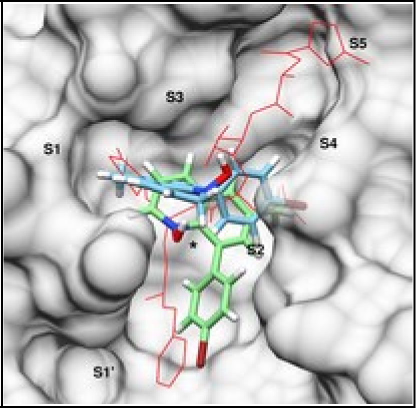
**43**	-29.7	-9.5	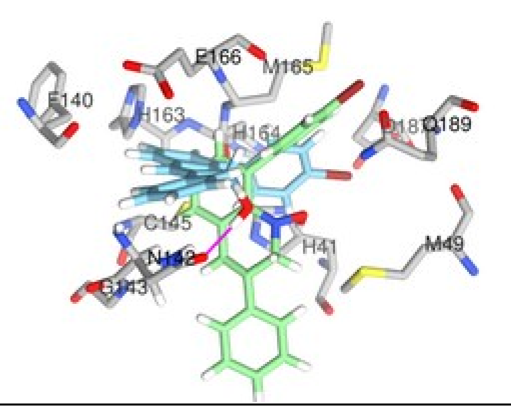	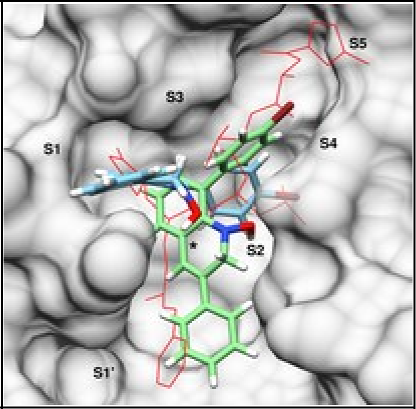
**44**	-30.2	-10.4	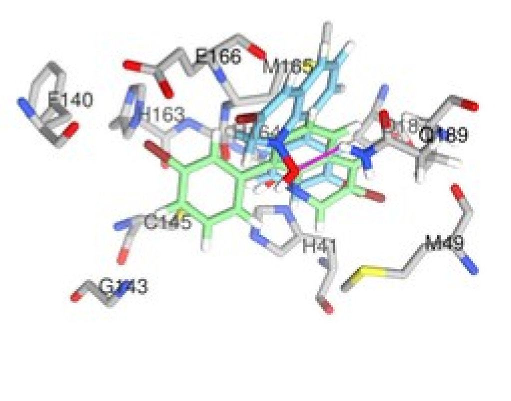	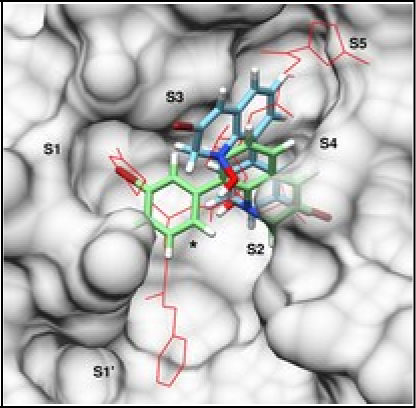
**45**	-23.9	-10.2	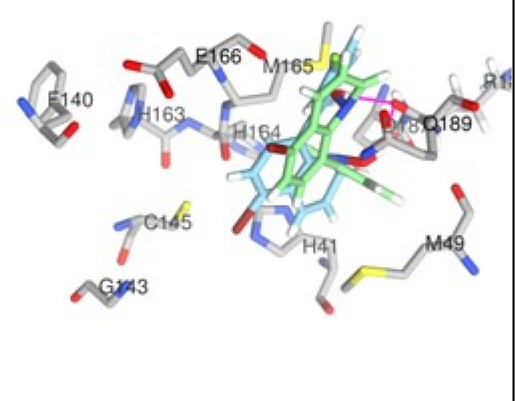	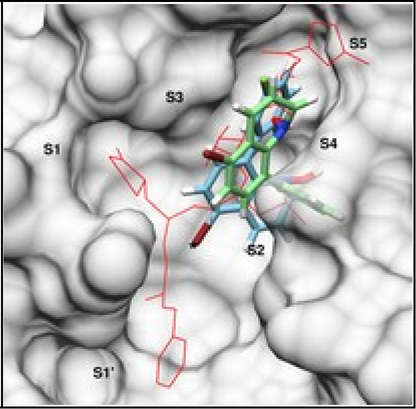

**46**	-23.2	-10.2	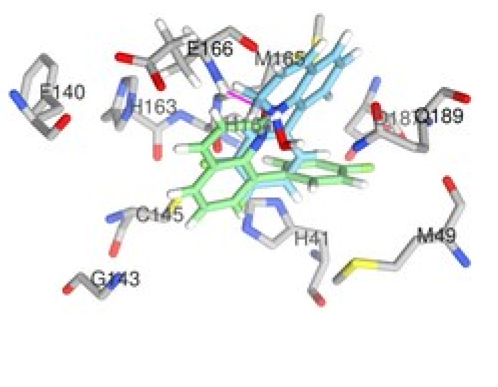	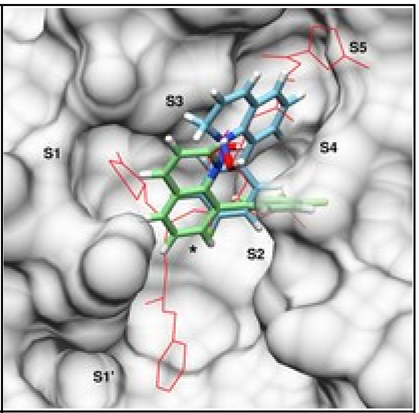
**47**	-28.5	-9.2	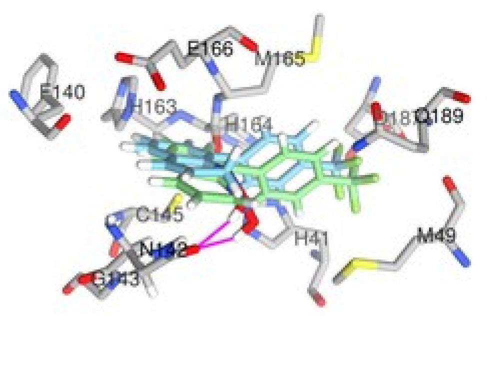	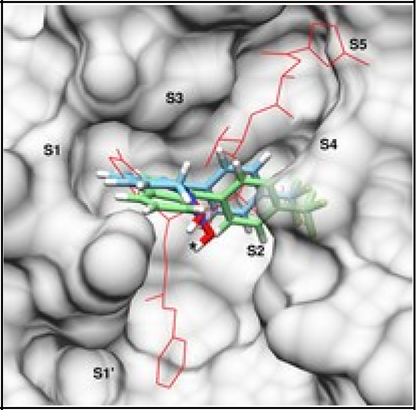
**48**	-29.7	-10.9	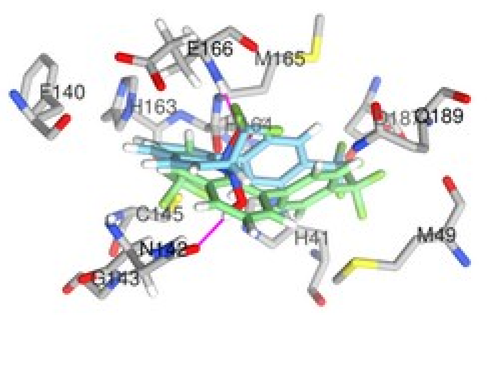	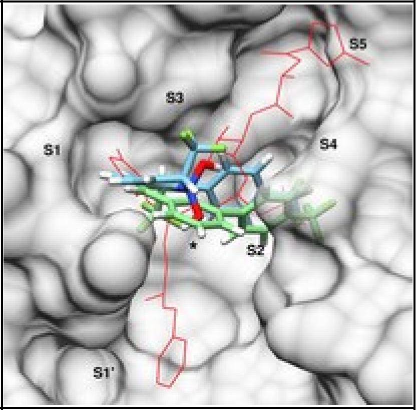
**49**	-30.2	-10.4	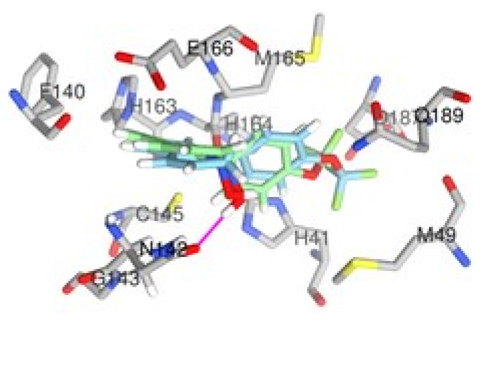	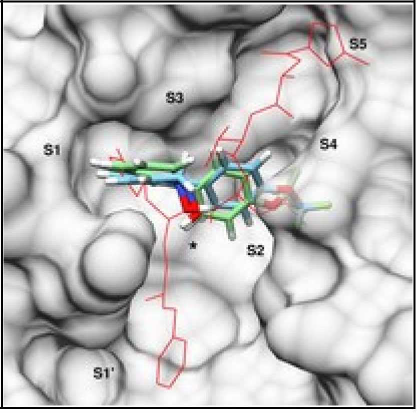

**50**	-30.1	-10.4	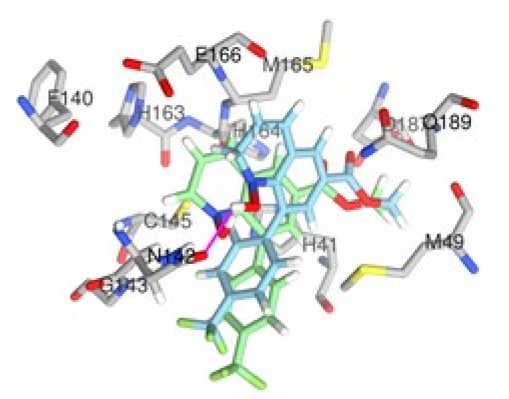	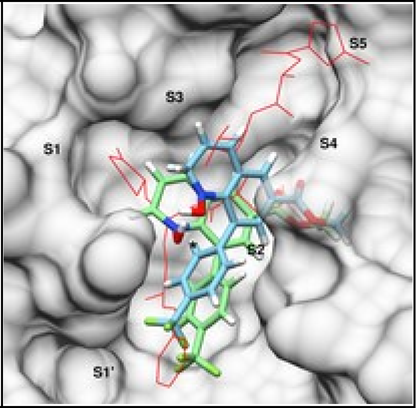
**51**	-27.4	-10.6	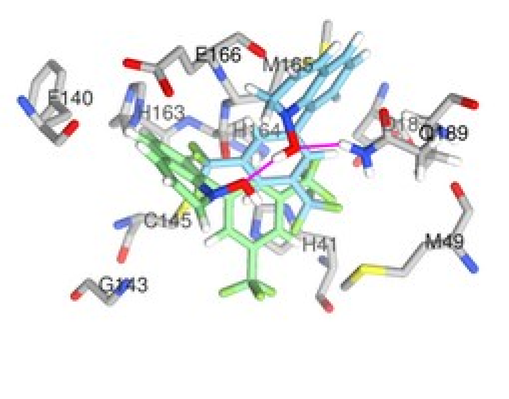	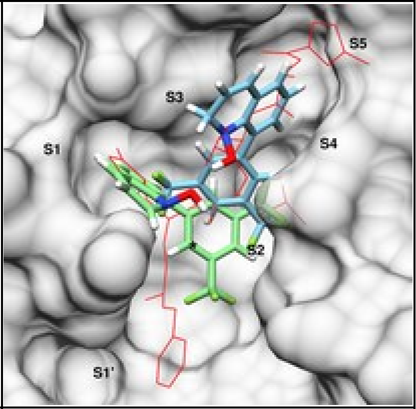
**52**	-30.6	-10.3	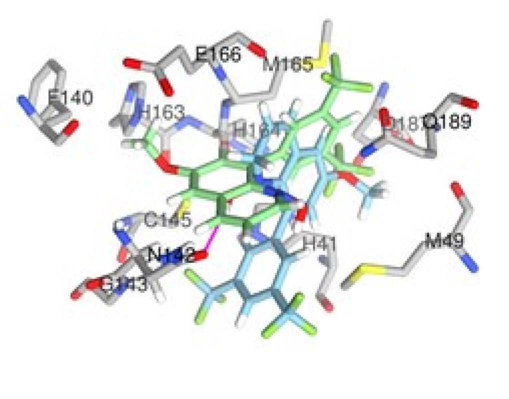	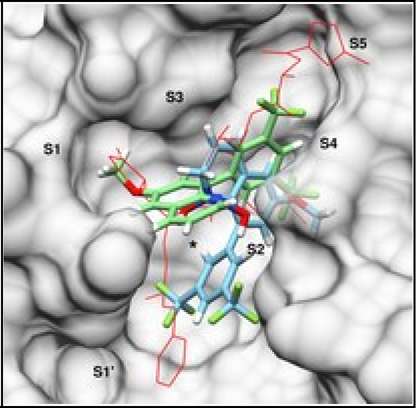
**PS**	-26.5	-10.1	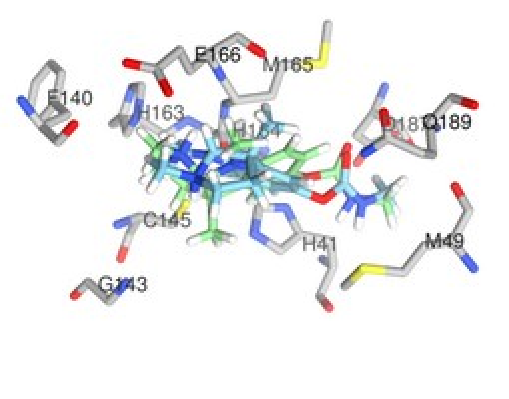	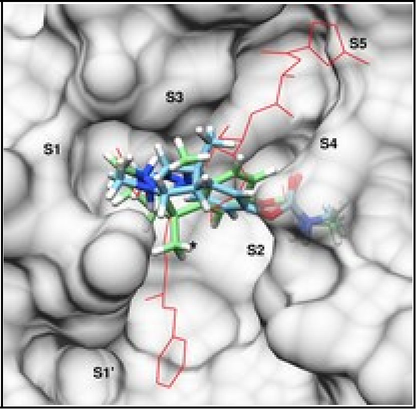

<table>
<tr><td>53</td><td>-28.6</td><td>-6.7</td><td>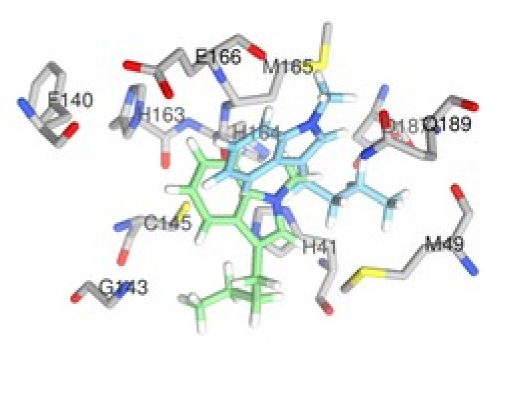
</td><td>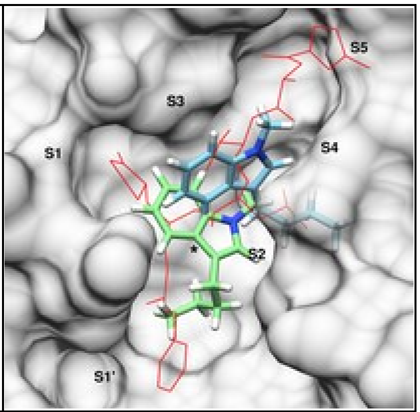
</td></tr>
<tr><td>54</td><td>-31.1</td><td>-9.1</td><td>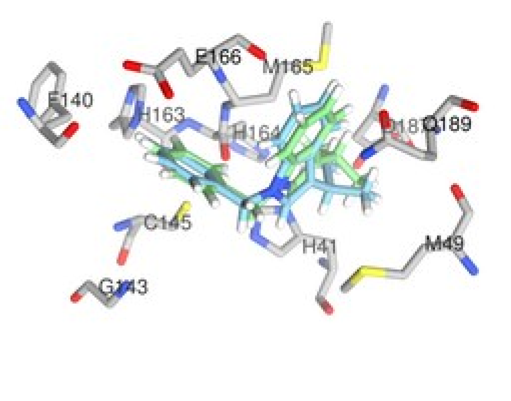
</td><td>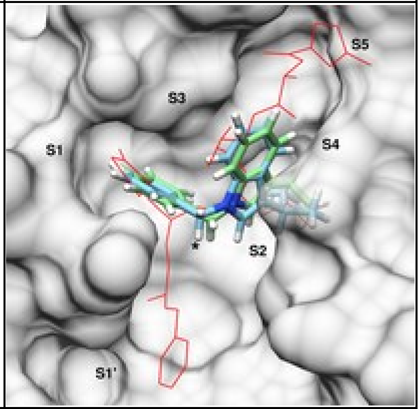
</td></tr>
<tr><td>55</td><td>-30.5</td><td>-9.1</td><td>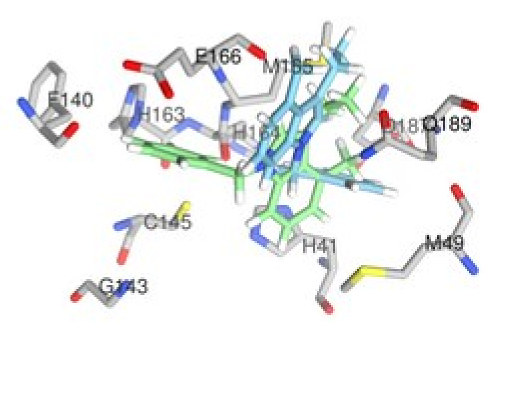
</td><td>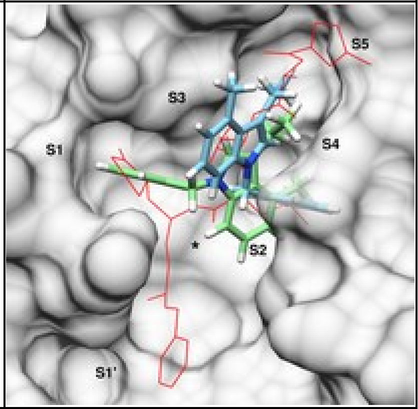
</td></tr>
<tr><td>56</td><td>-26.4</td><td>-8.9</td><td>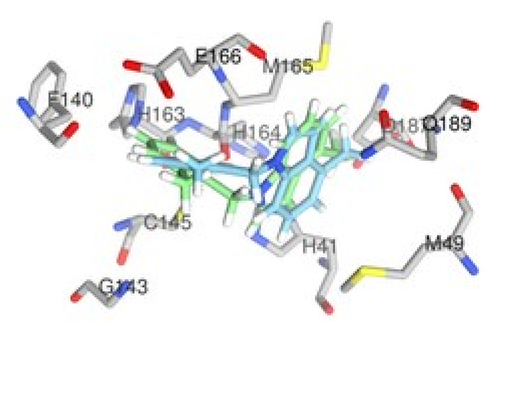
</td><td>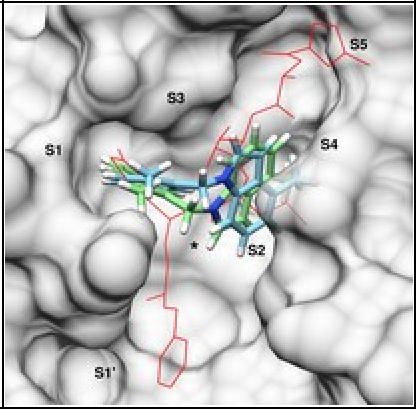
</td></tr>
</table>

**57**	-28.3	-8.4	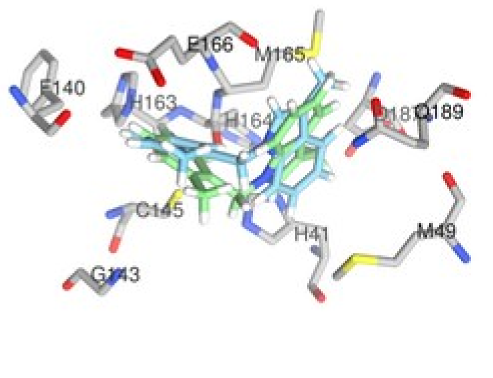	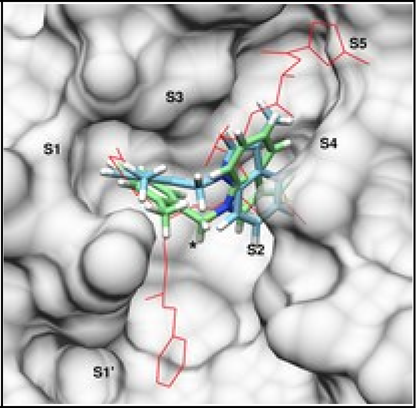
**58**	-30.7	-9.0	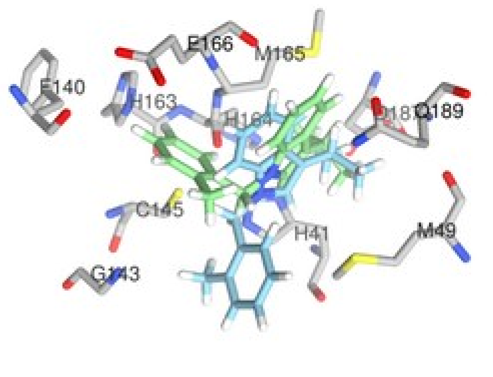	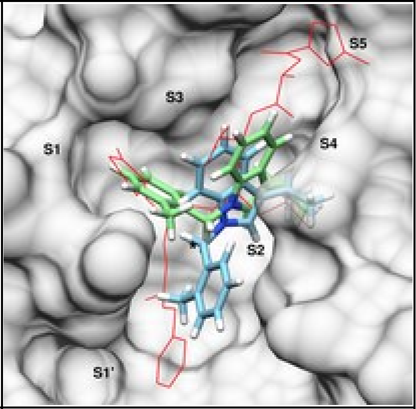
**59**	-29.6	-8.7	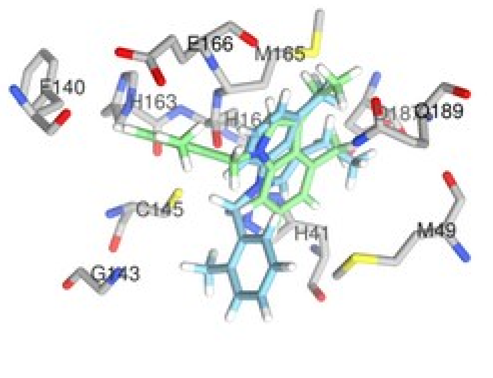	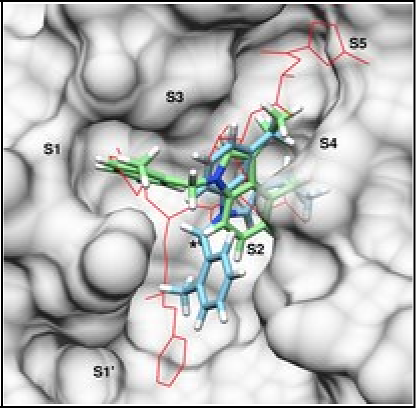
**60**	-28.0	-9.5	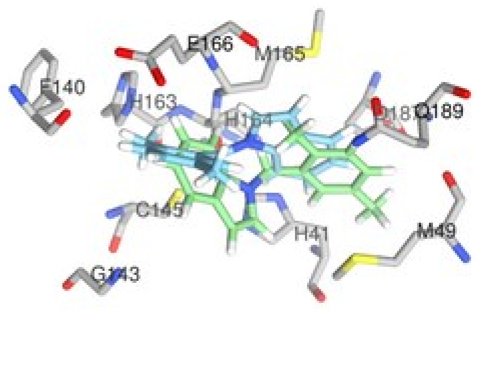	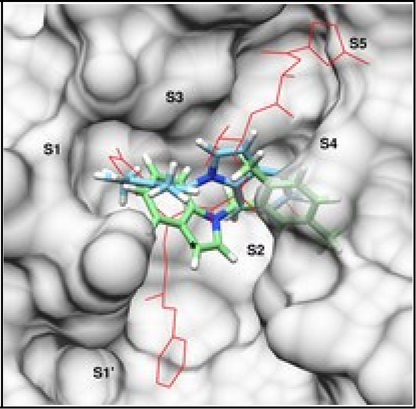

**61**	-28.6	-9.8	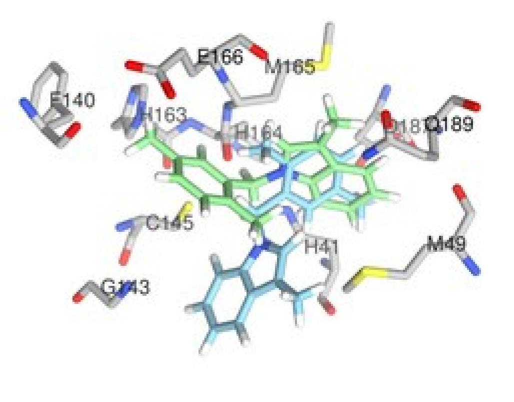	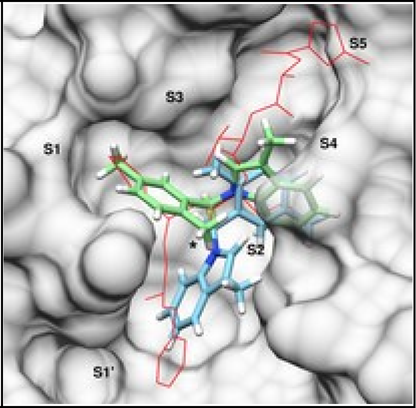
**62**	-31.9	-9.6	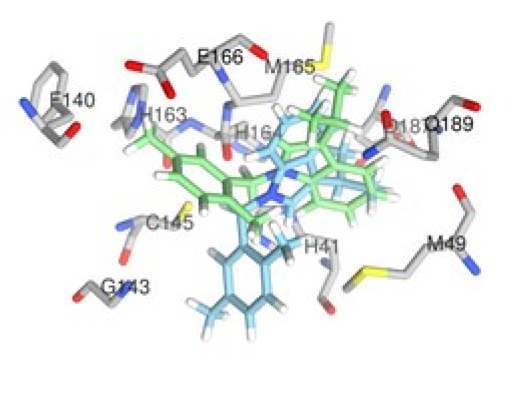	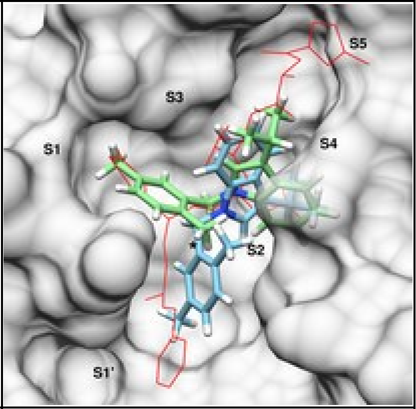
**63**	-32.9	-9.3	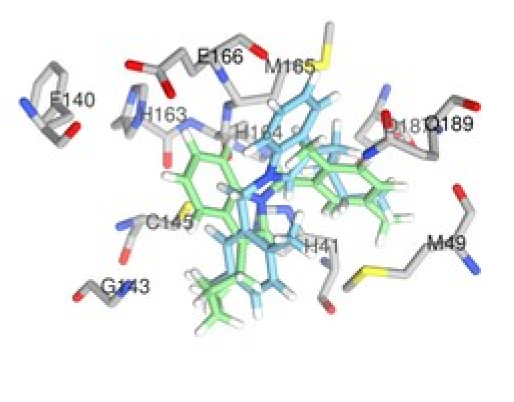	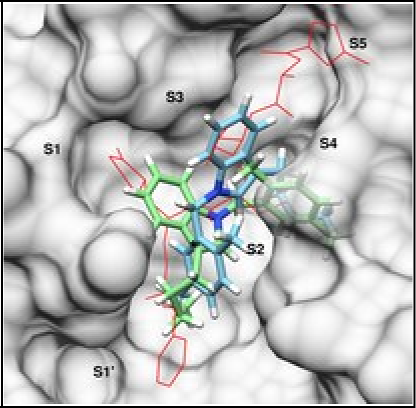
**64**	-31.3	-9.6	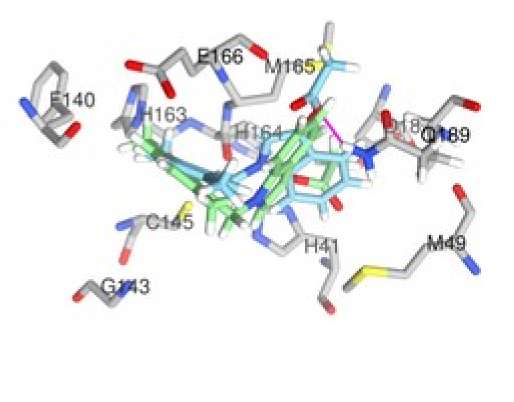	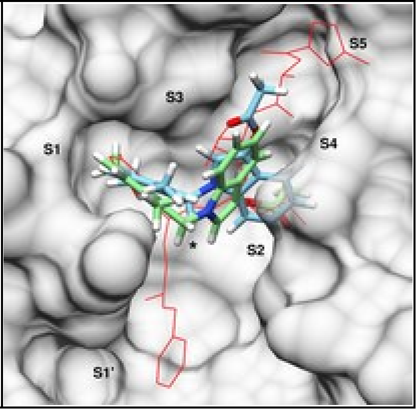

**65**	-31.7	-11.2	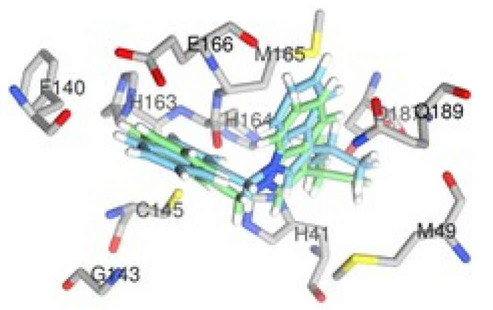	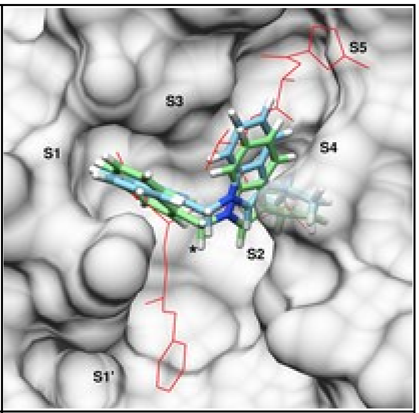
**66**	-33.0	-13.0	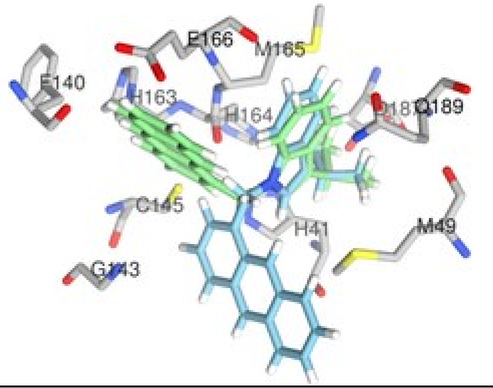	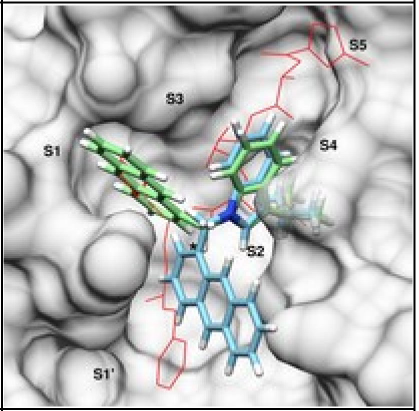
**67**	-27.0	-11.8	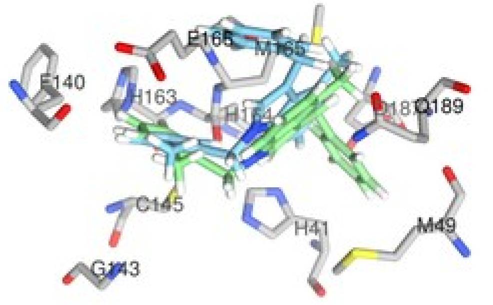	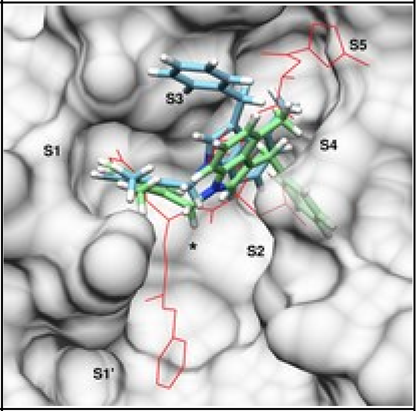
**68**	-29.9	-11.5	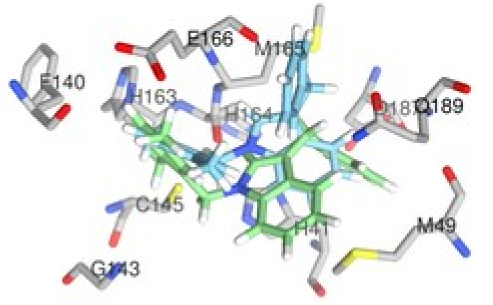	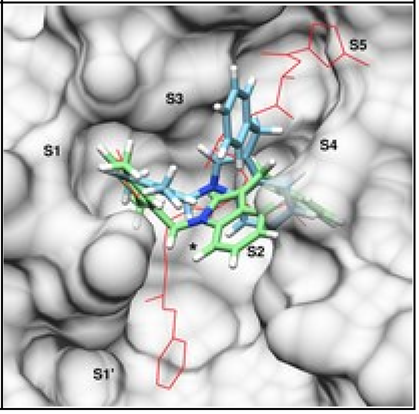

**69**	-26.4	-8.4	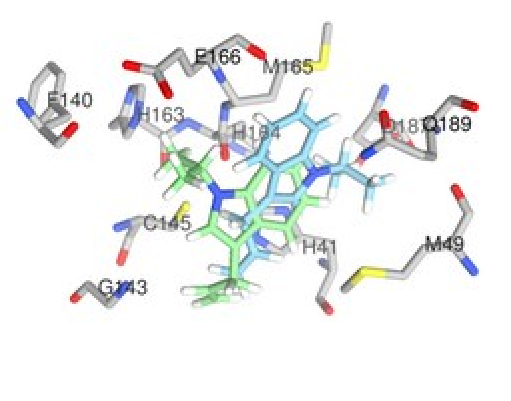	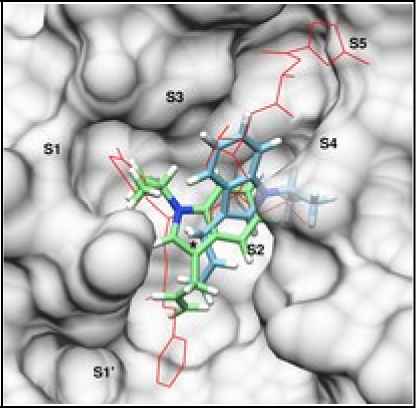
**70**	-31.3	-8.7	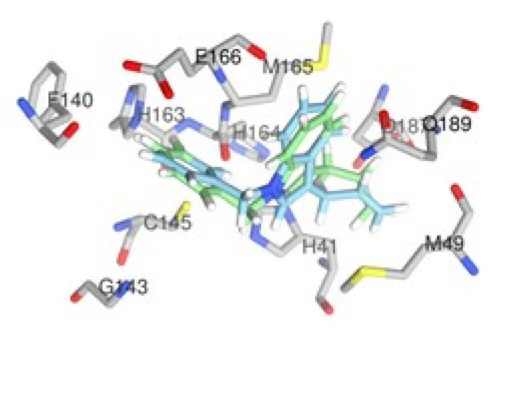	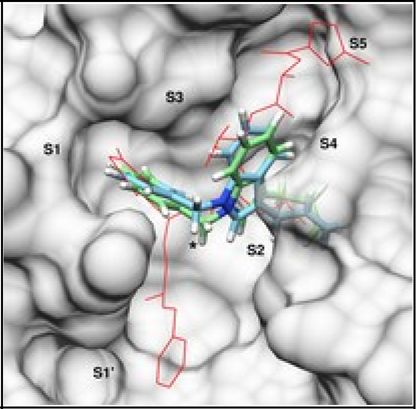
**71**	-30.6	-9.8	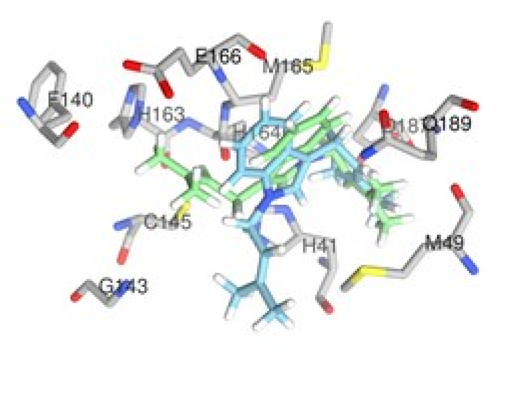	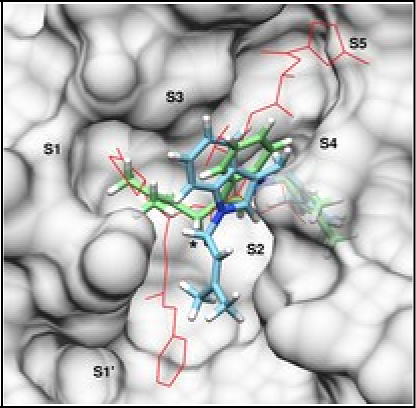
**72**	-28.5	-9.3	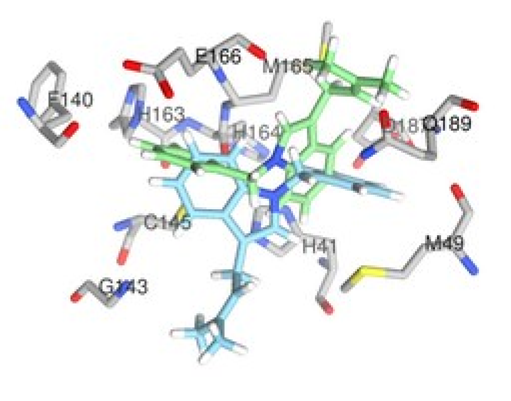	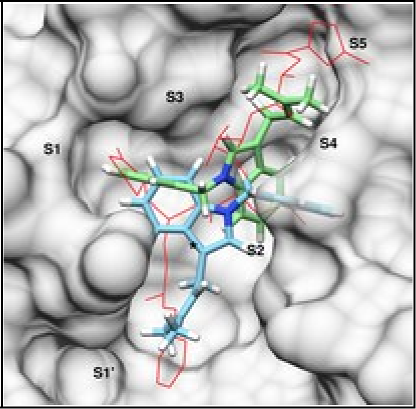

<table>
<tr><td>73</td><td>-30.6</td><td>-8.6</td><td>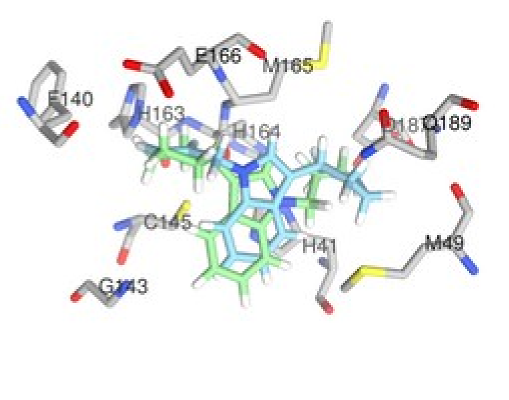</td><td>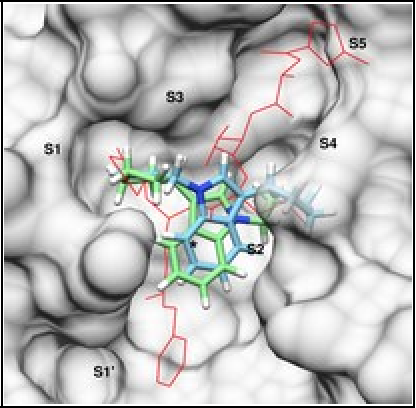</td></tr>
<tr><td>74</td><td>-25.9</td><td>-8.8</td><td>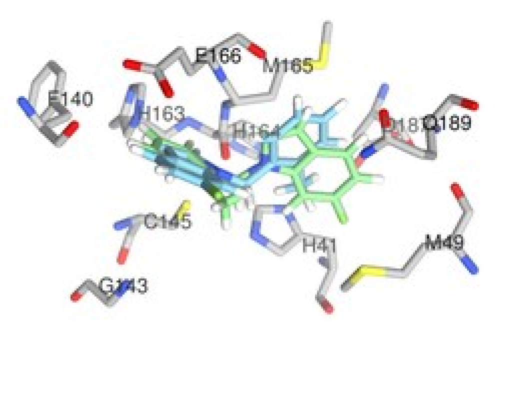</td><td>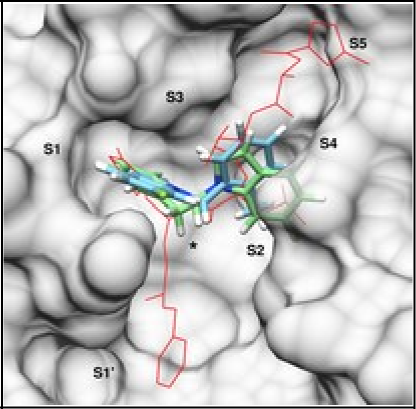</td></tr>
<tr><td>75</td><td>-28.8</td><td>-9.6</td><td>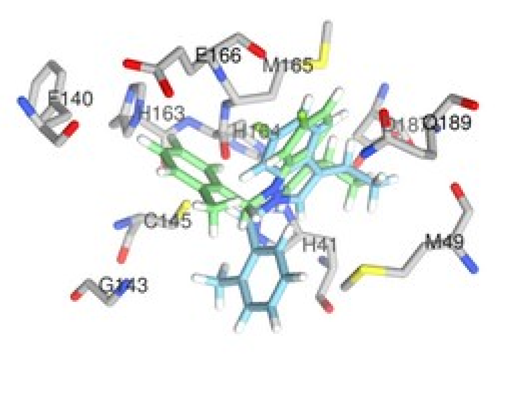</td><td>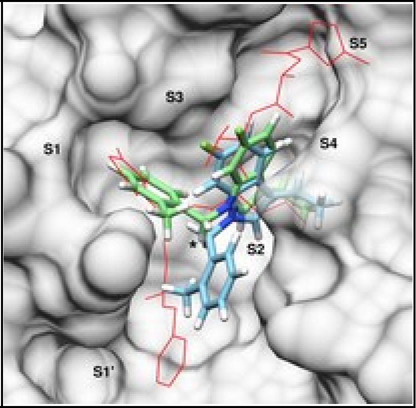</td></tr>
<tr><td>76</td><td>-34.7</td><td>-10.0</td><td>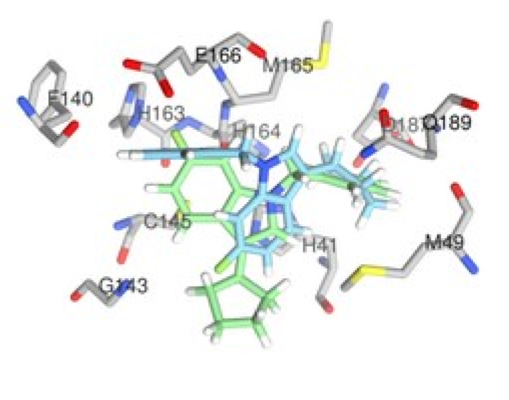</td><td>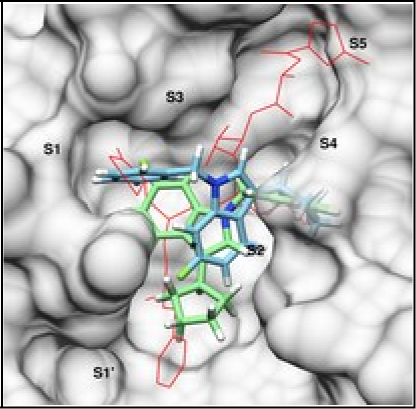</td></tr>
</table>

**77**	-30.8	-10.9	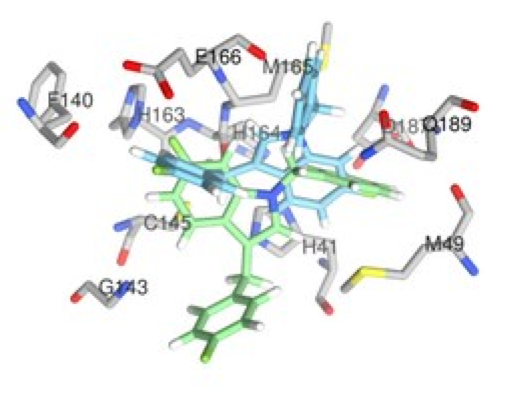	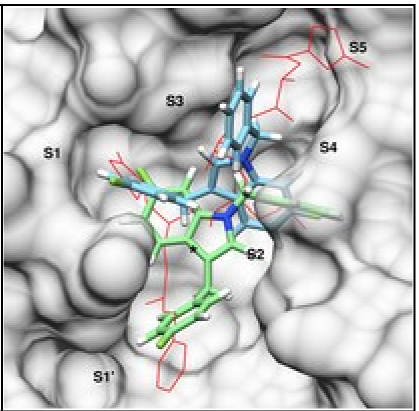
**78**	-32.8	-10.4	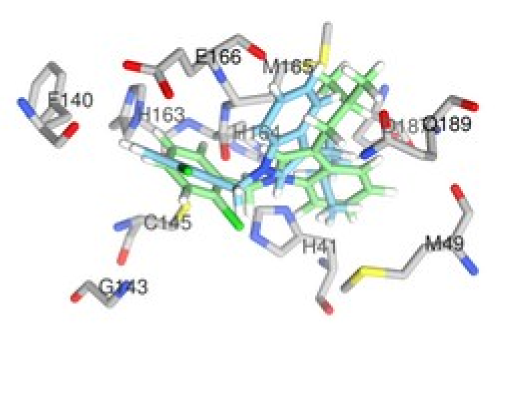	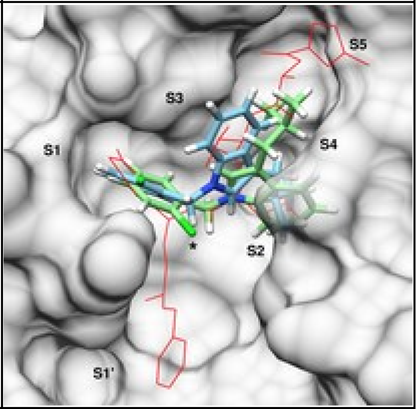
**79**	-28.1	-7.3	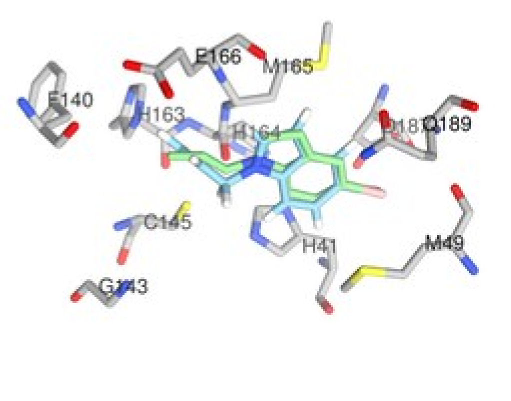	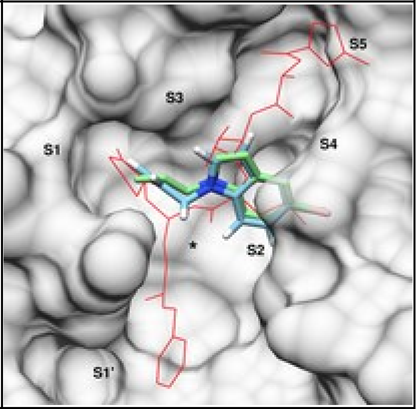
**80**	-28.9	-8.5	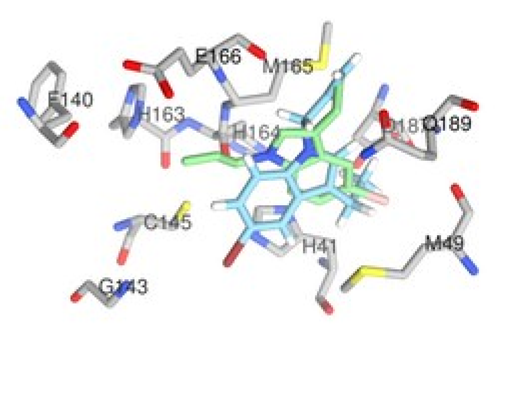	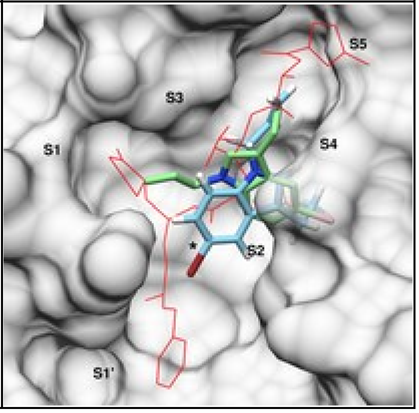

**81**	-33.3	-8.2	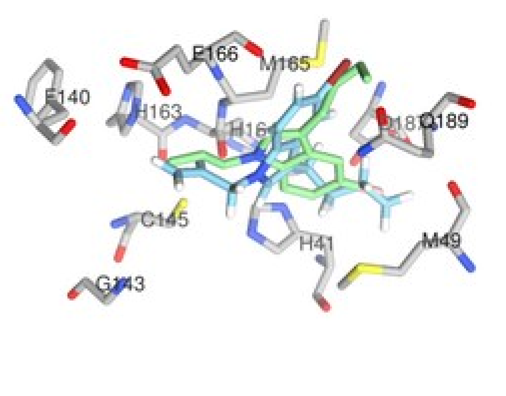	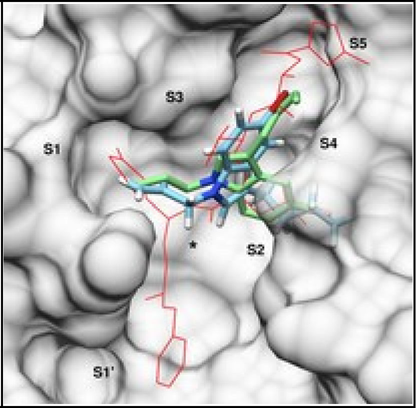
**82**	-33.5	-8.5	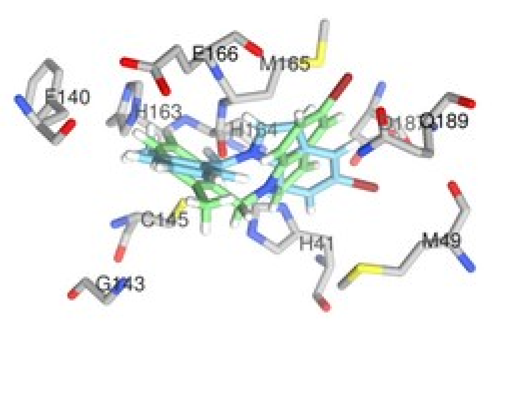	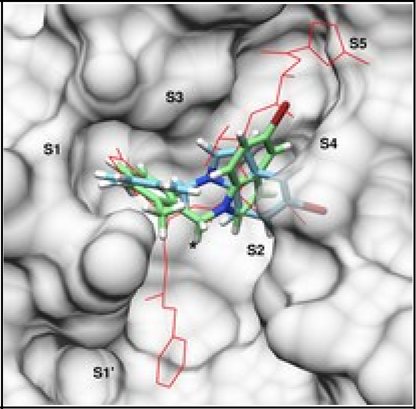
**83**	-33.9	-9.0	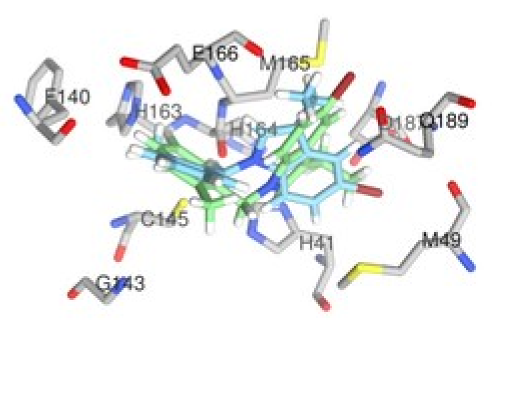	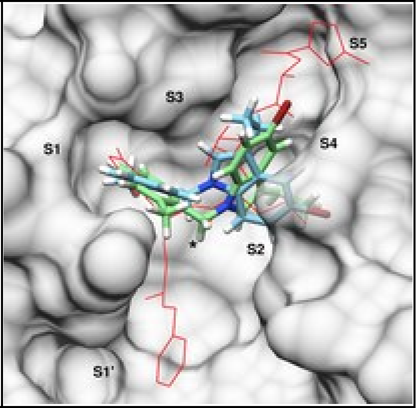
**84**	-37.3	-12.9	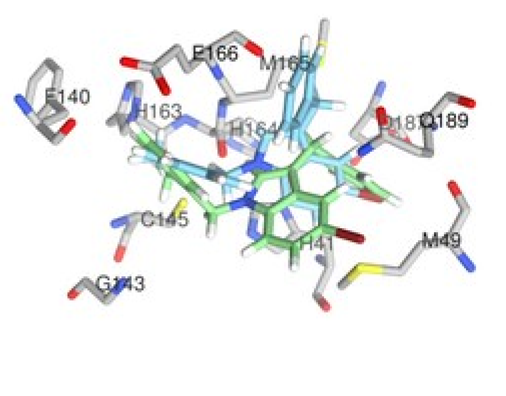	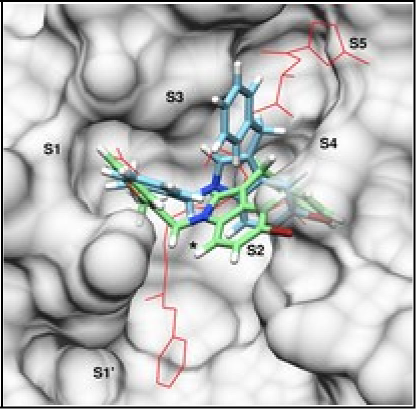

**85**	-33.2	-8.4	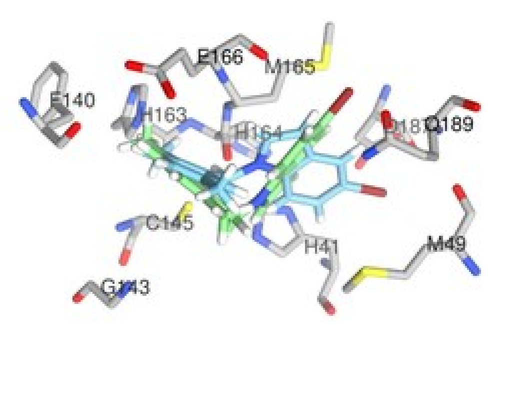	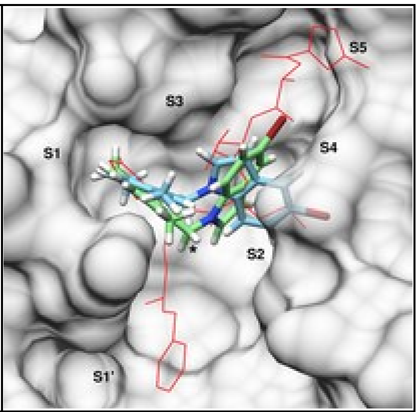
**86**	-35.0	-8.6	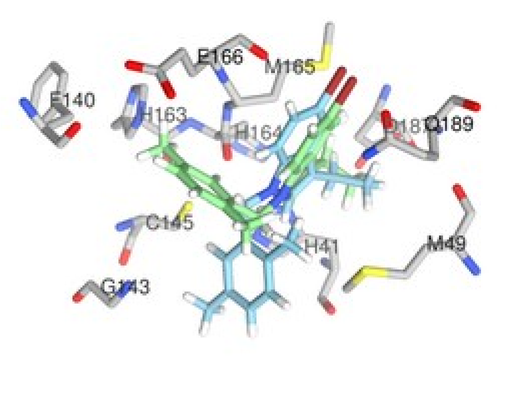	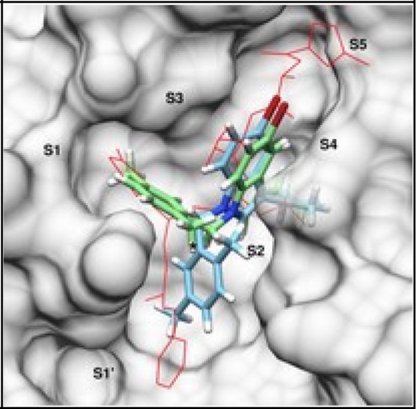
**87**	-38.0	-11.9	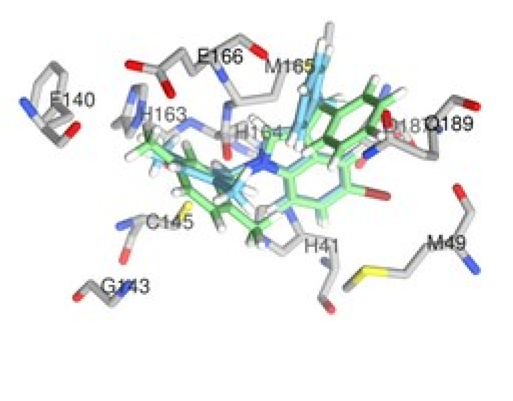	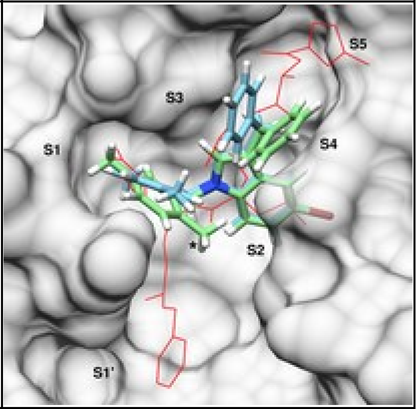
**88**	-33.5	-9.1	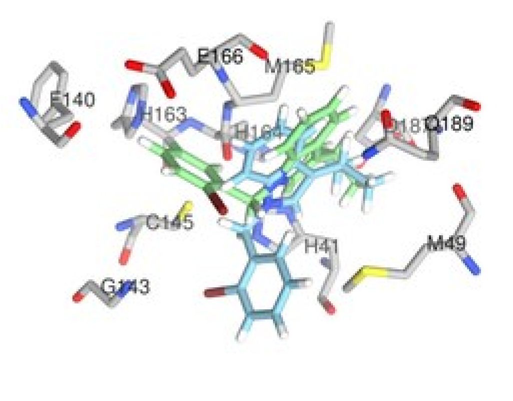	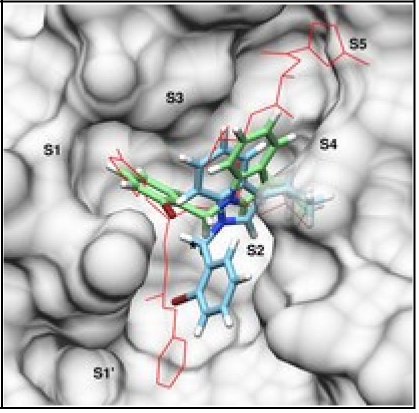

**89**	-33.6	-8.9	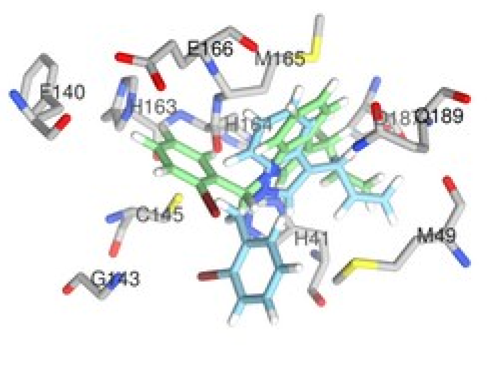	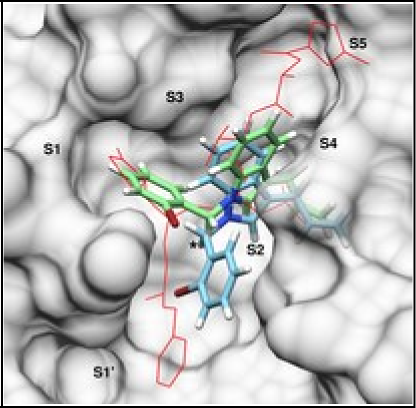
**90**	-34.7	-9.4	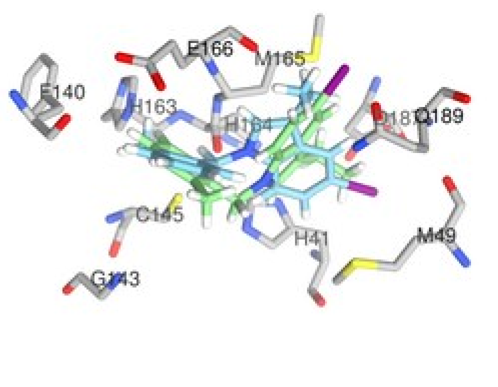	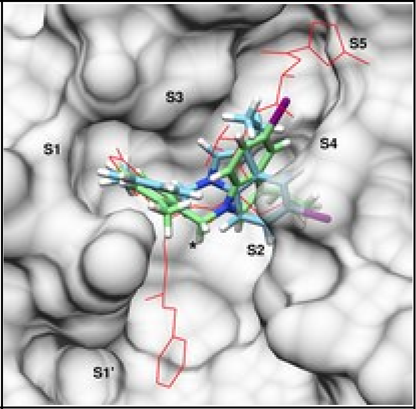
**91_SR**	-30.2	-8.4	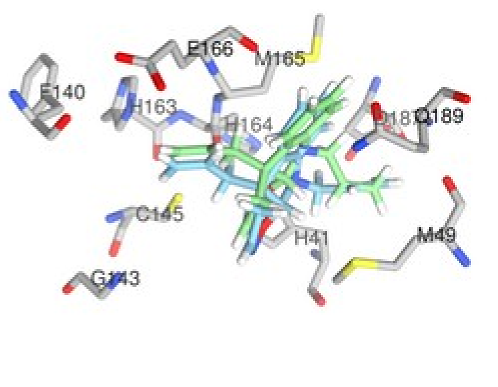	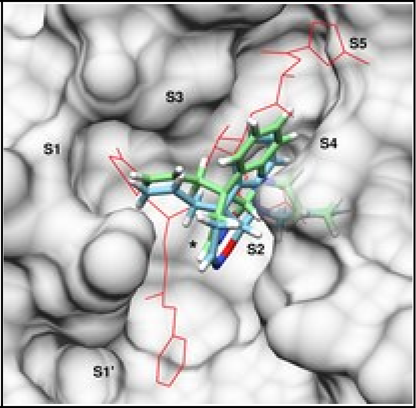
**91_SS**	-26.6	-7.8	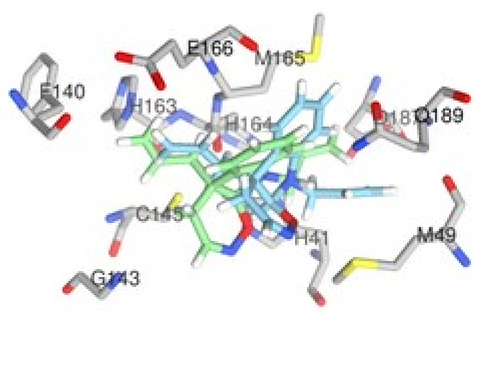	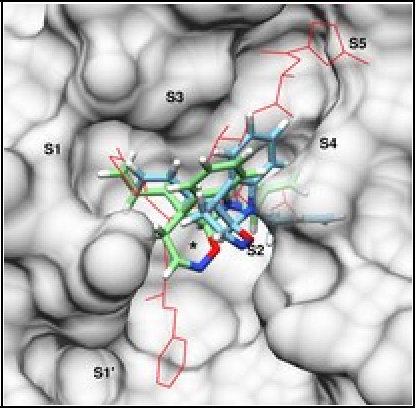

**91_RR**	-25.7	-9.3	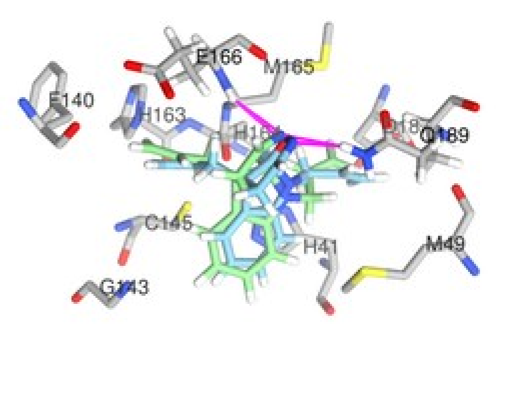	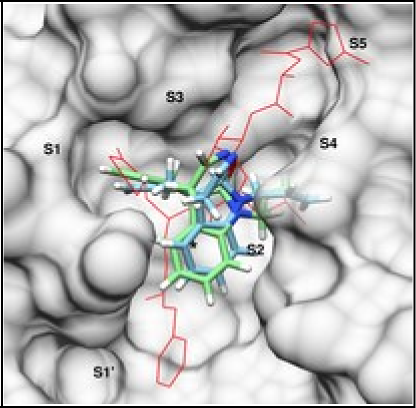
**91_RS**	-27.6	-7.5	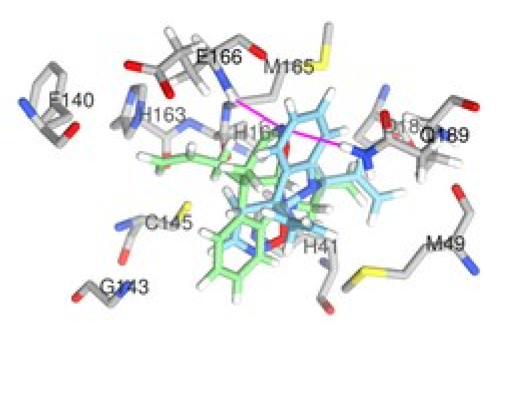	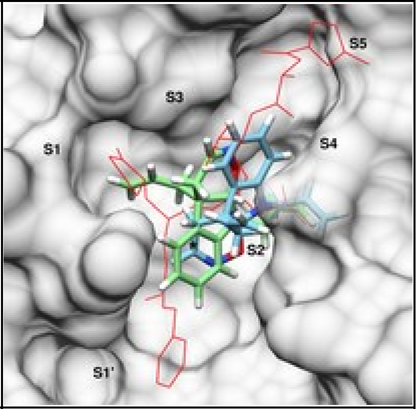
**92_SR**	-31.6	-8.8	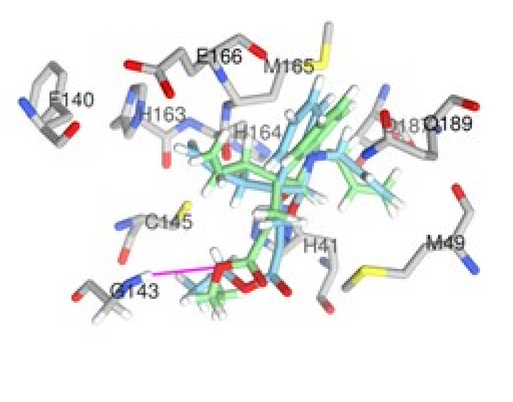	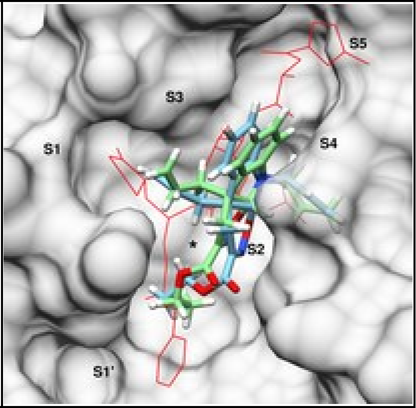
**92_SS**	-32.8	-8.7	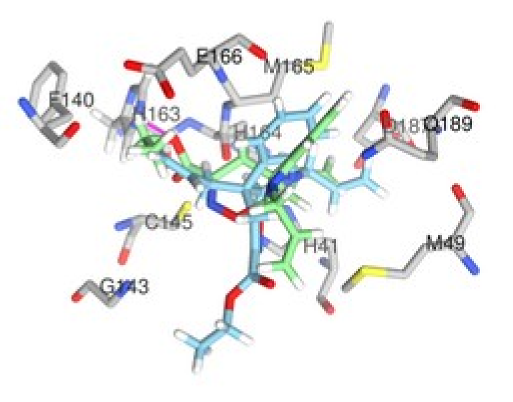	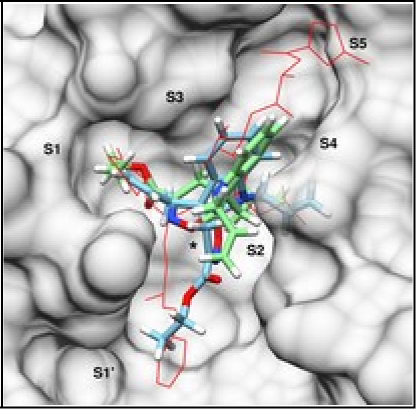

**92_RR**	-31.2	-8.6	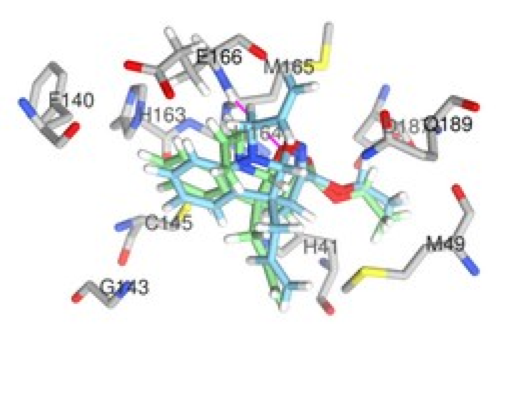	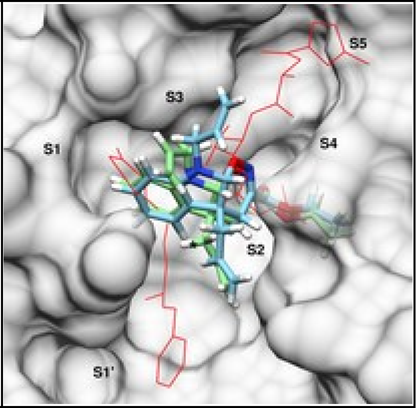
**92_RS**	-33.3	-10.3	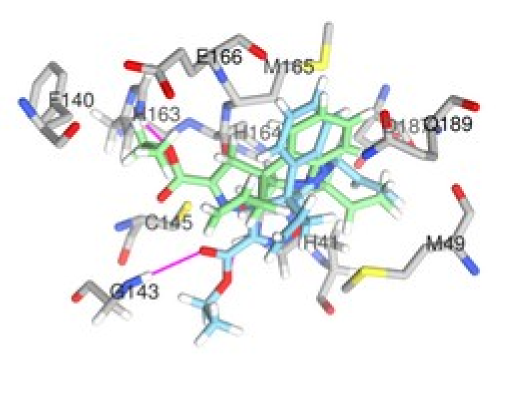	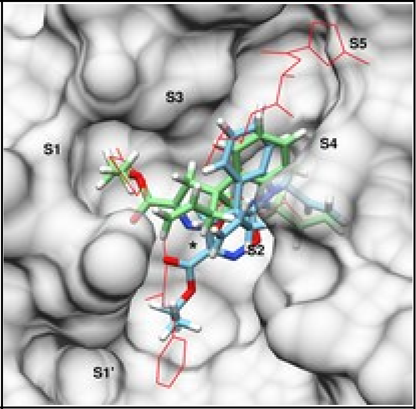
**93_SR**	-31.8	-9.7	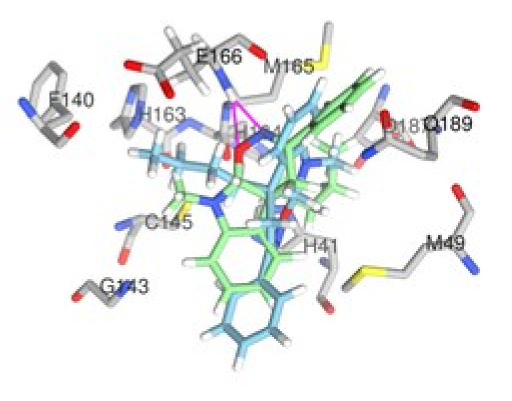	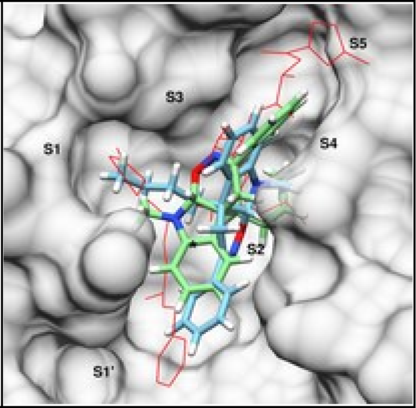
**93_SS**	-31.8	-11.3	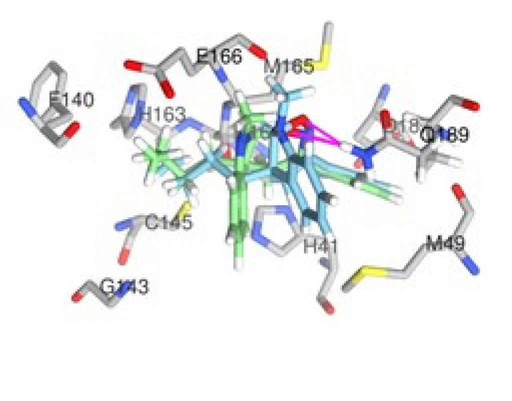	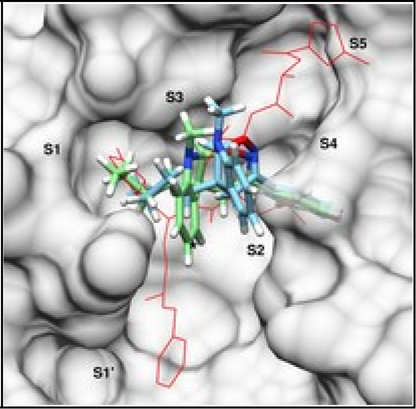

**93_RR**	-32.8	-9.5	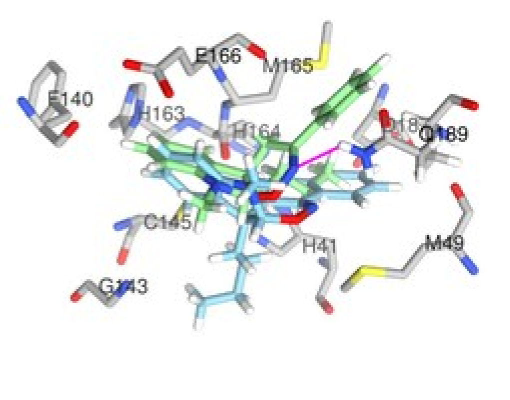	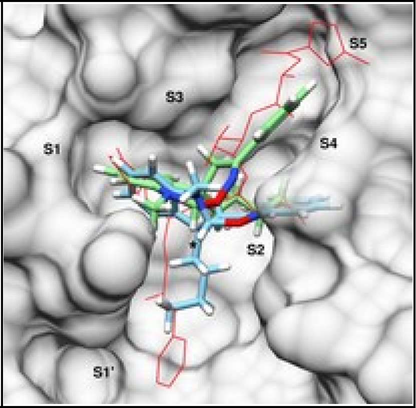
**93_RS**	-34.4	-9.3	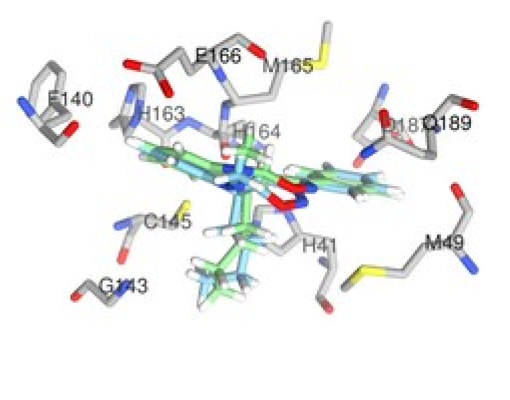	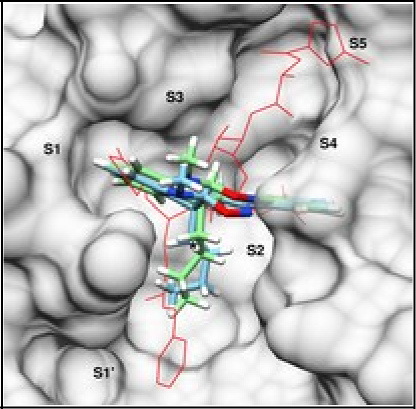
**94_SR**	-27.9	-10.1	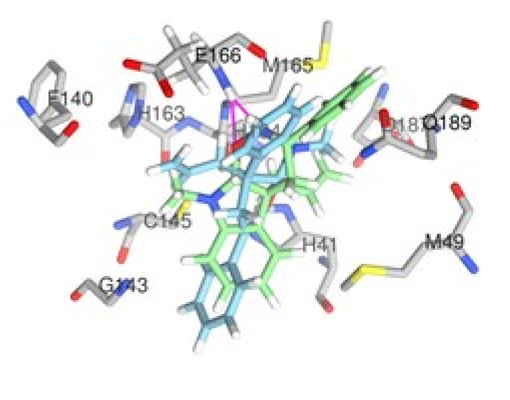	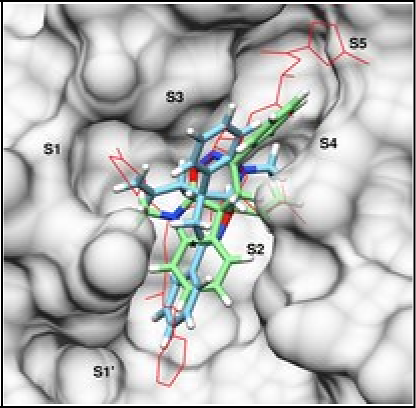
**94_SS**	-26.2	-9.2	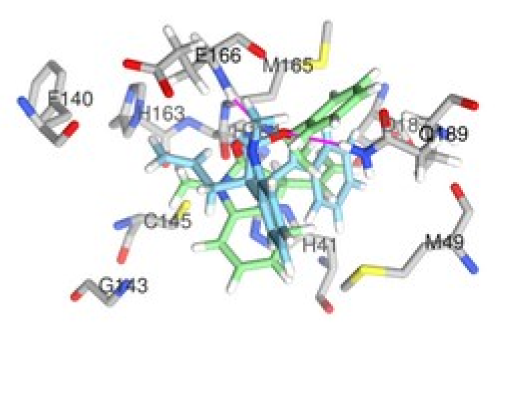	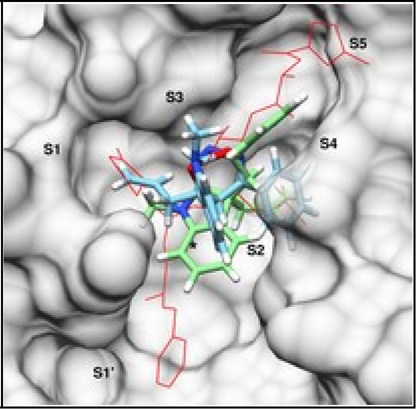

**94_RR**	-28.6	-9.3	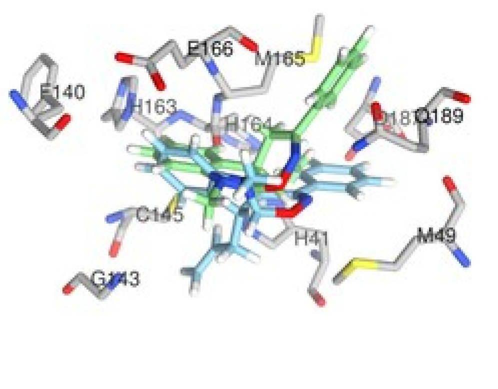	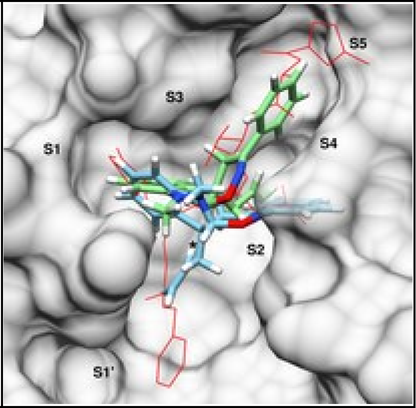
**94_RS**	-28.8	-11.1	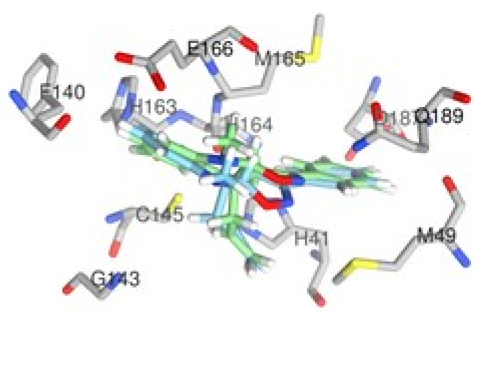	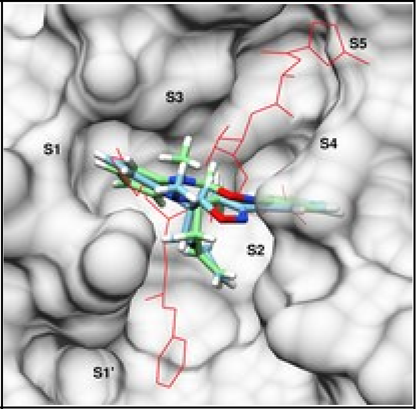
**95_SR**	-32.4	-9.7	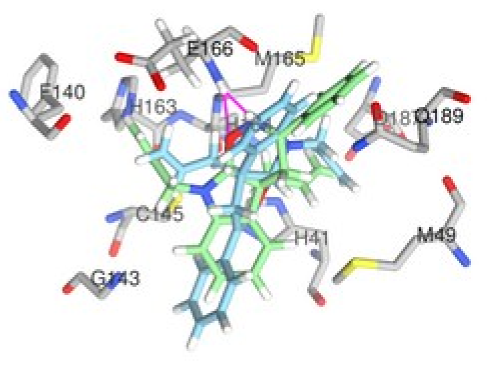	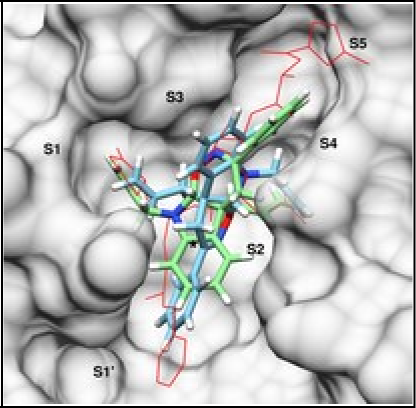
**95_SS**	-30.4	-9.8	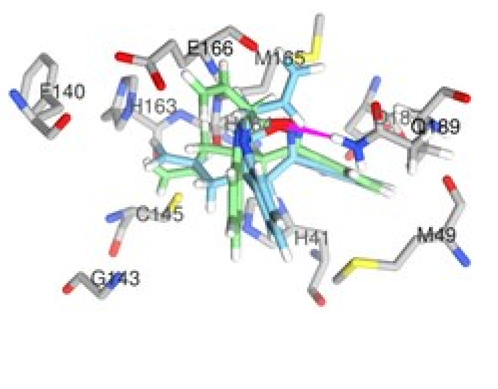	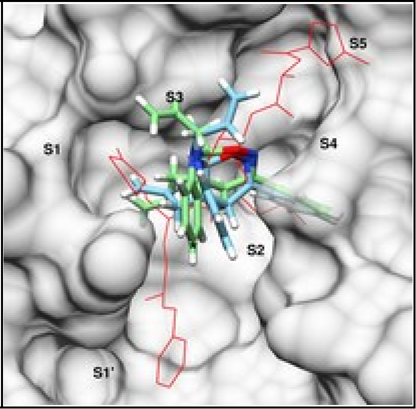

**95_RR**	-30.6	-9.1	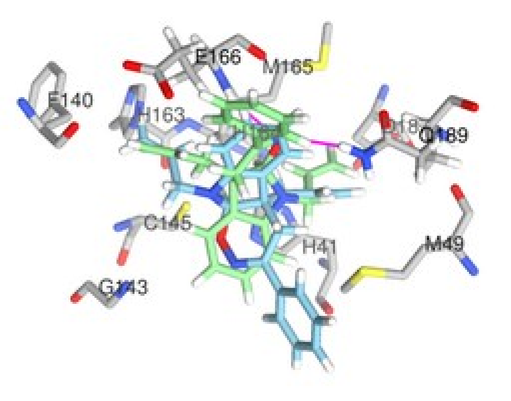	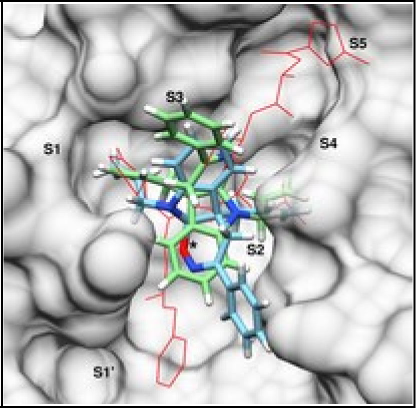
**95_RS**	-31.2	-9.0	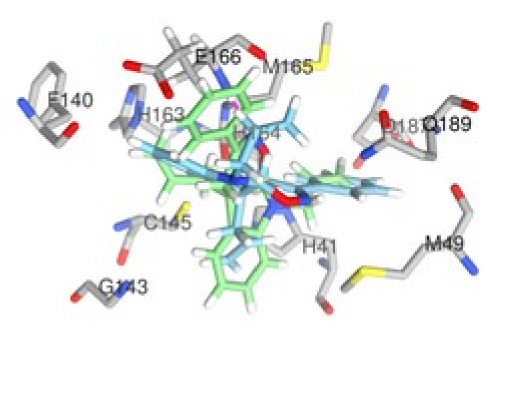	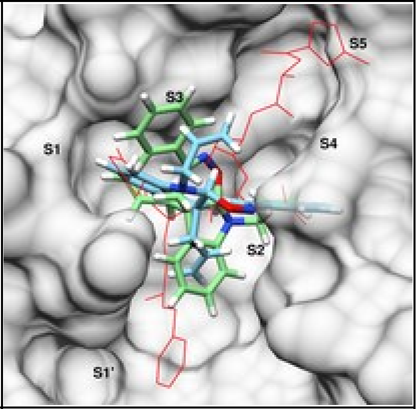
**96_RR**	-34.0	-9.7	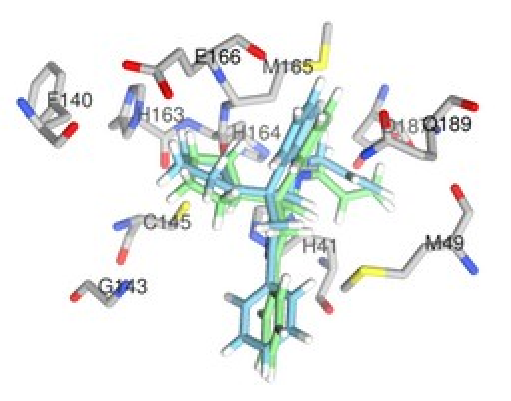	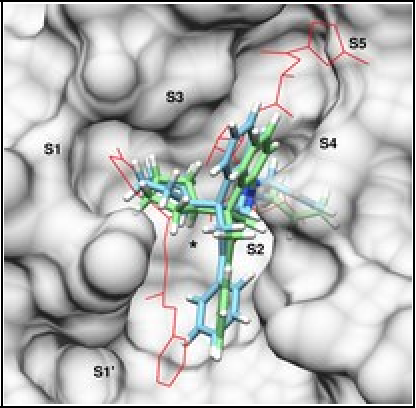
**96_RS**	-33.6	-10.5	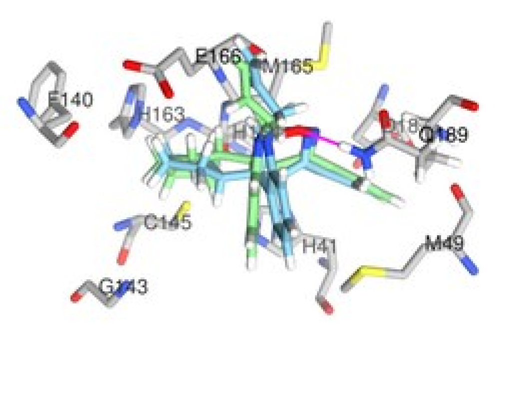	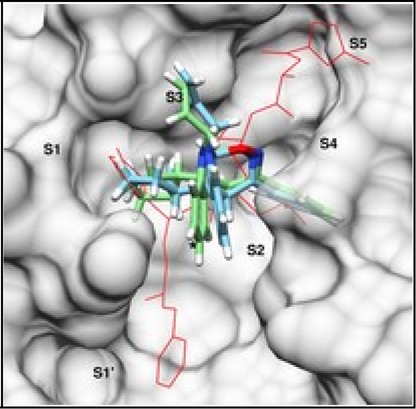

**96_SR**	-30.2	-10.4	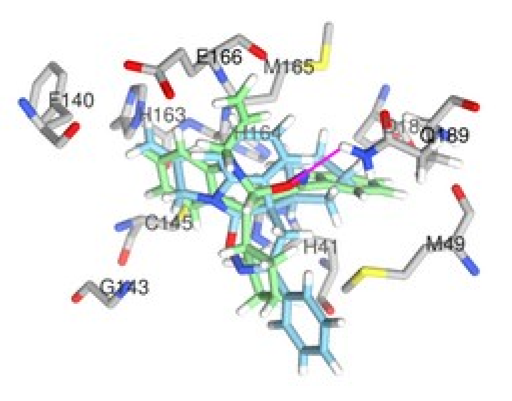	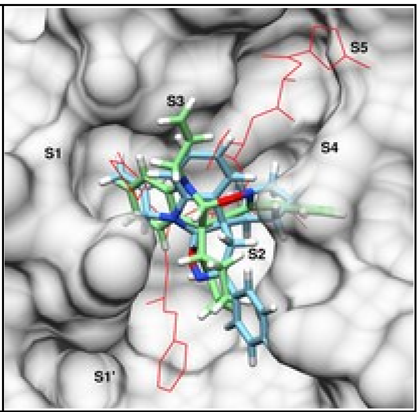
**96_SS**	-29.4	-10.8	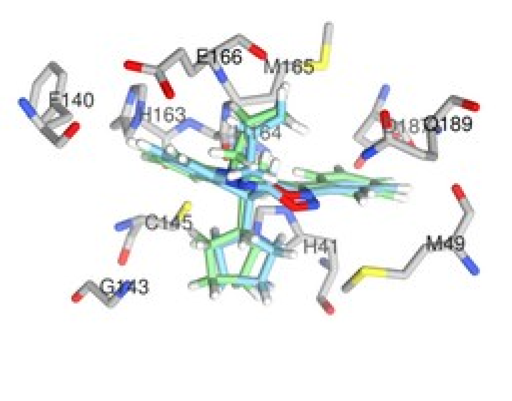	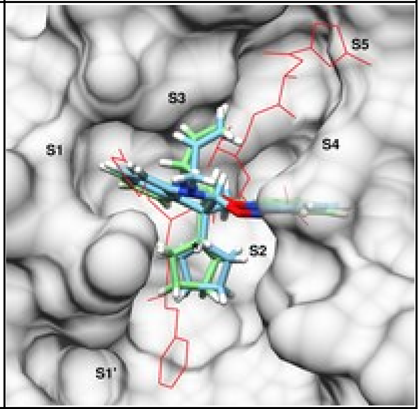
**97_SR**	-37.4	-10.5	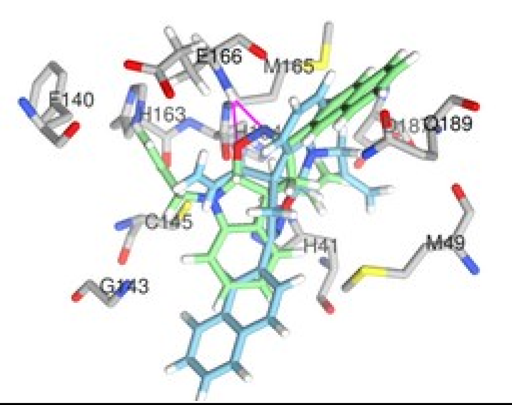	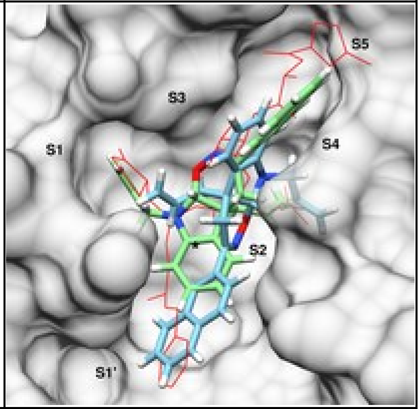
**97_SS**	-32.9	-9.8	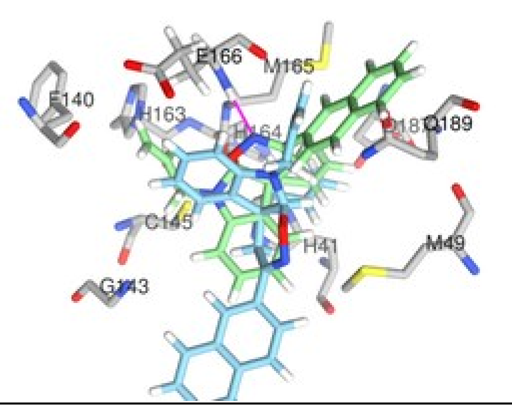	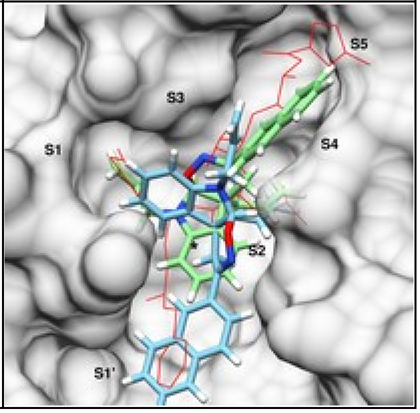

**97_RR**	-34.0	-10.0	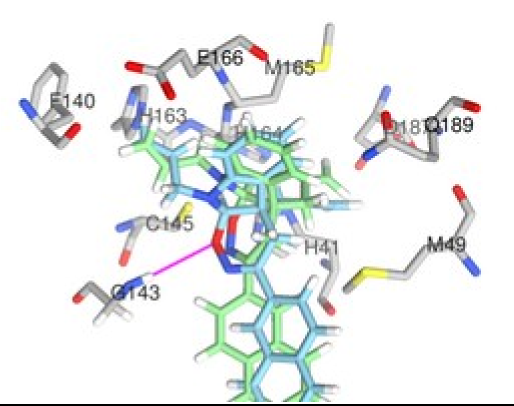	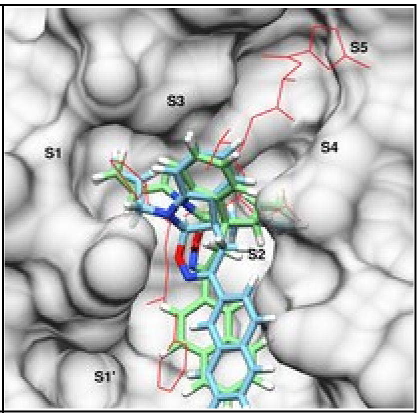
**97_RS**	-35.5	-9.7	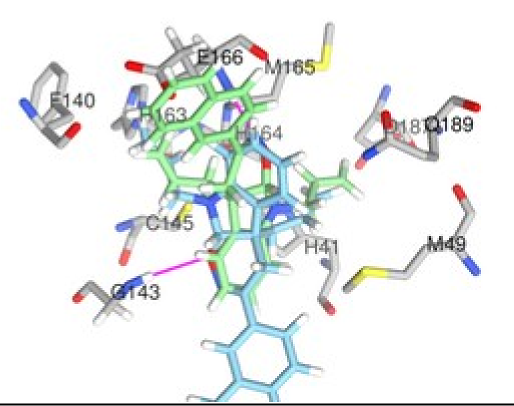	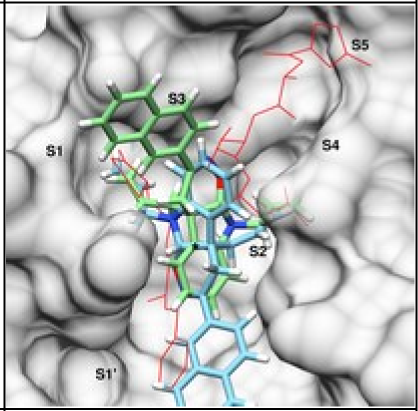
**98_SR**	-29.4	-10.7	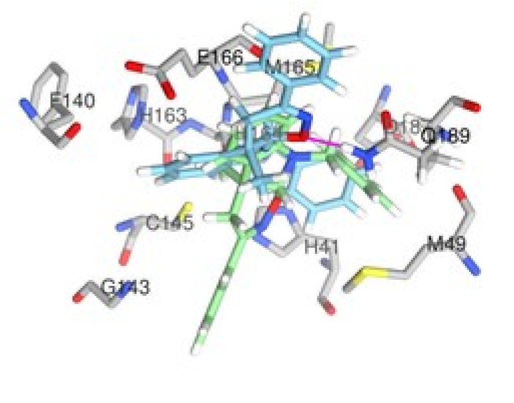	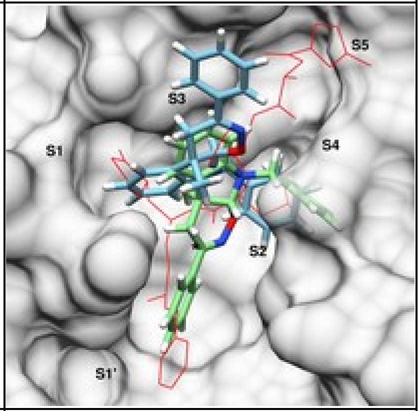
**98_SS**	-31.2	-11.0	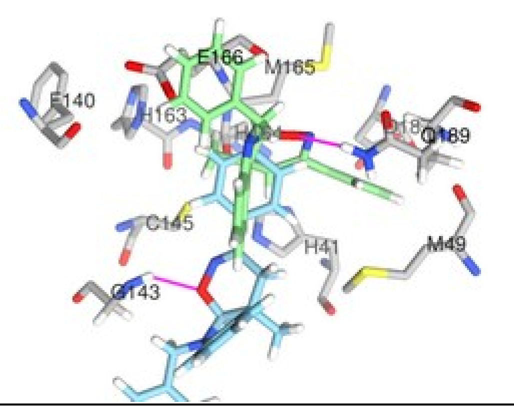	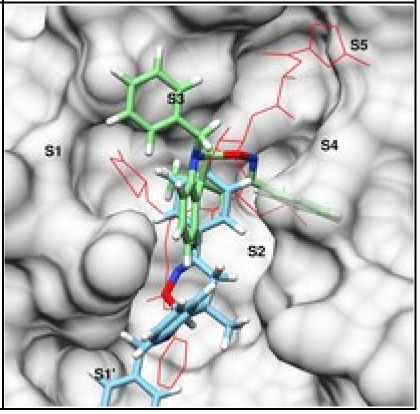

**98_RR**	-29.4	-12.8	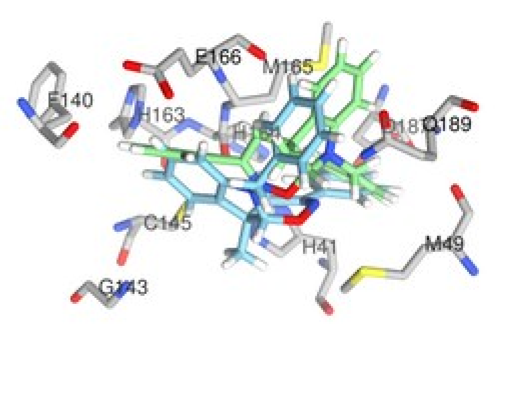	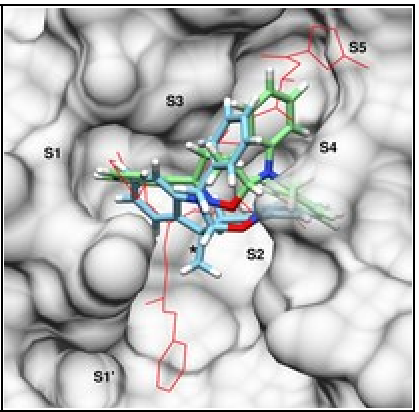
**98_RS**	-30.0	-10.7	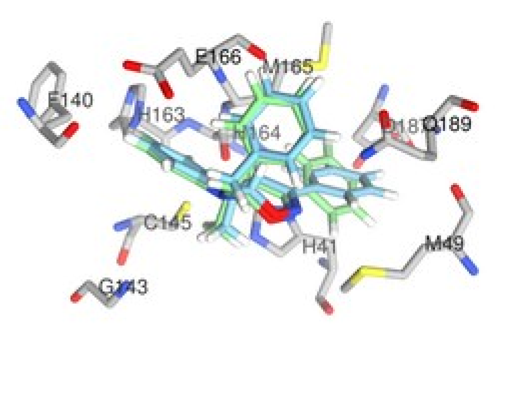	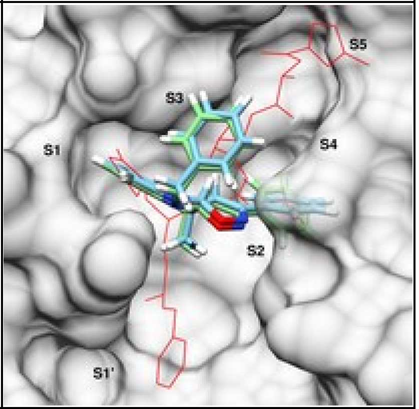
**99_RR**	-30.2	-12.2	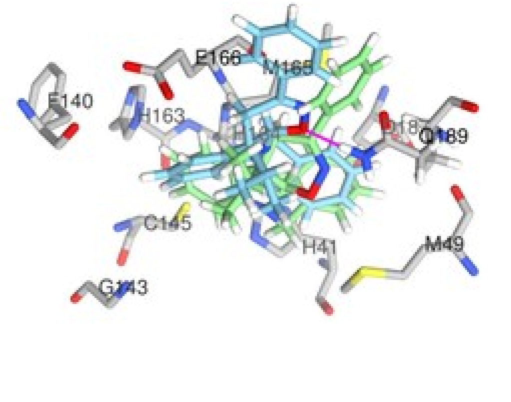	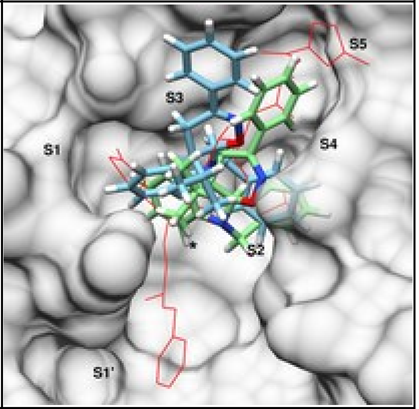
**99_RS**	-32.5	-11.3	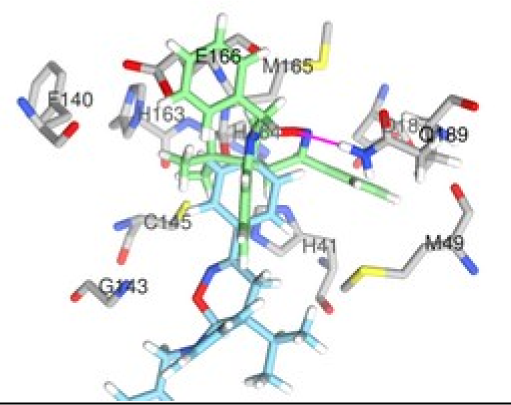	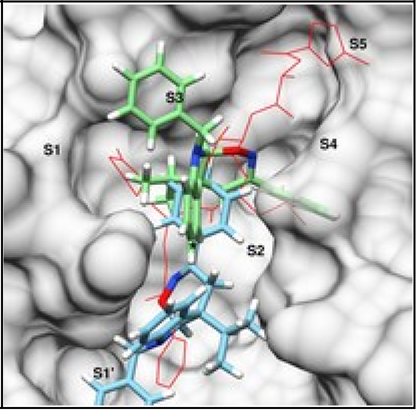

**99_SR**	-26.5	-13.4	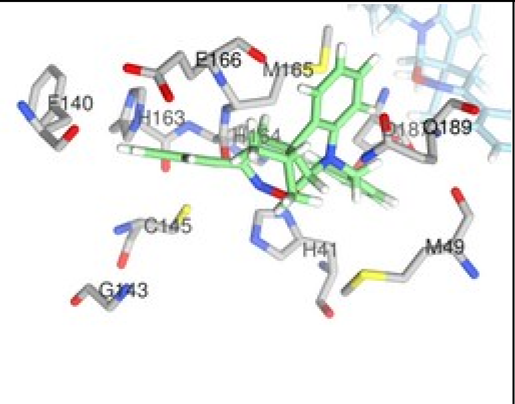	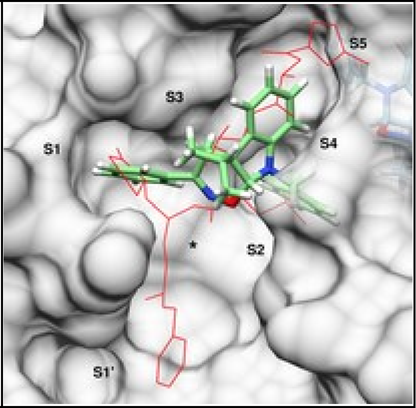
**99_SS**	-29.6	-11.4	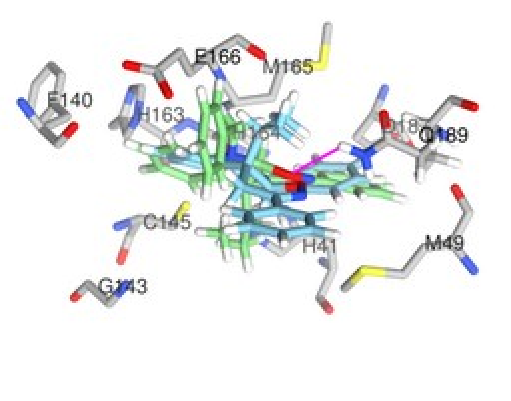	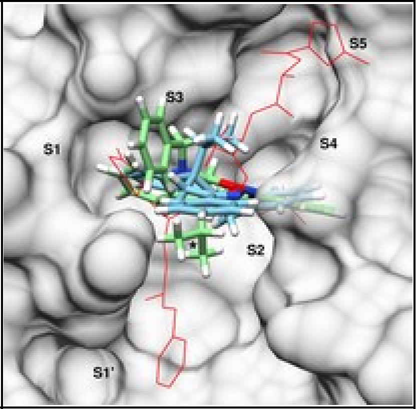
**100_SR**	-30.2	-11.6	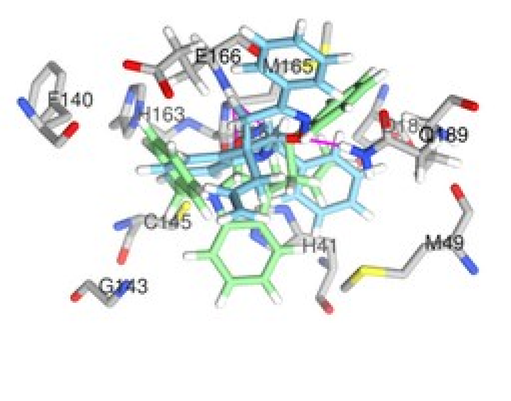	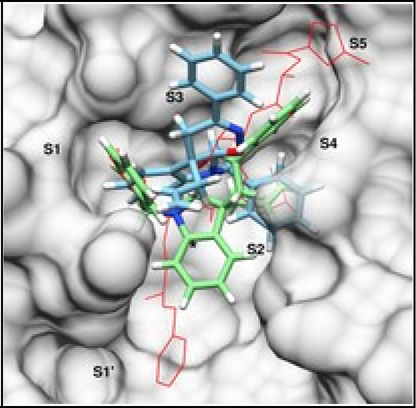
**100_SS**	-31.5	-9.8	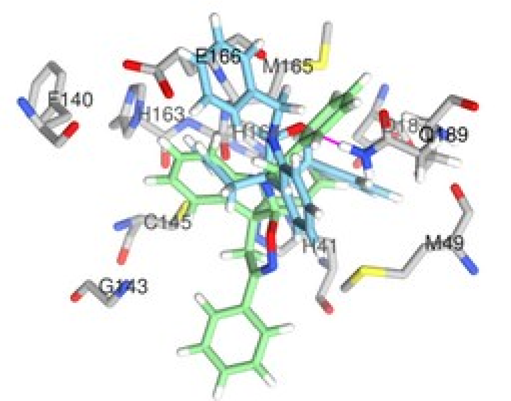	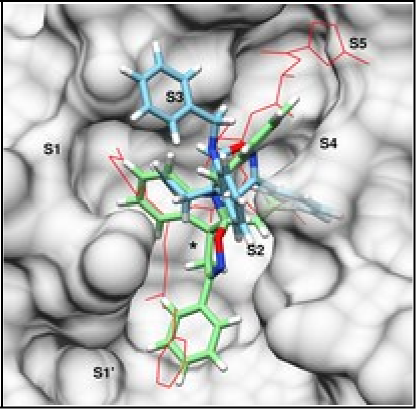

**100_RR**	-31.8	-12.3	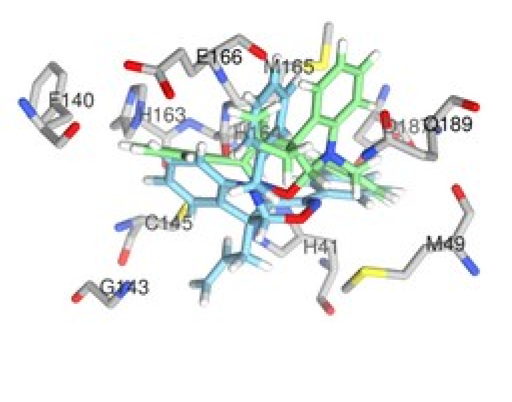	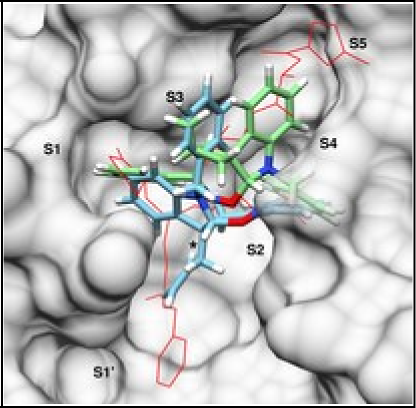
**100_RS**	-31.1	-9.8	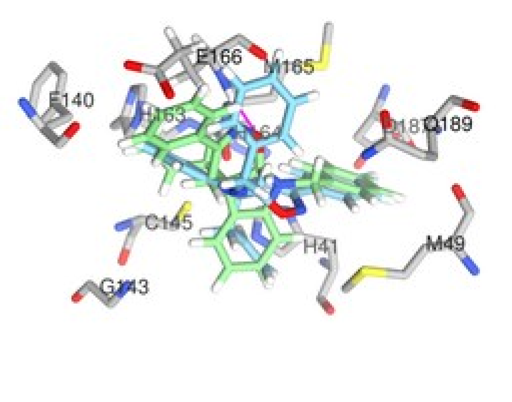	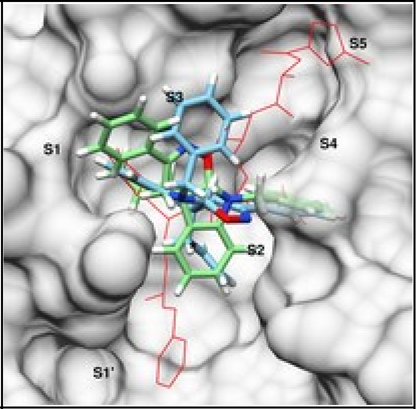
**101_RR**	-29.6	-11.0	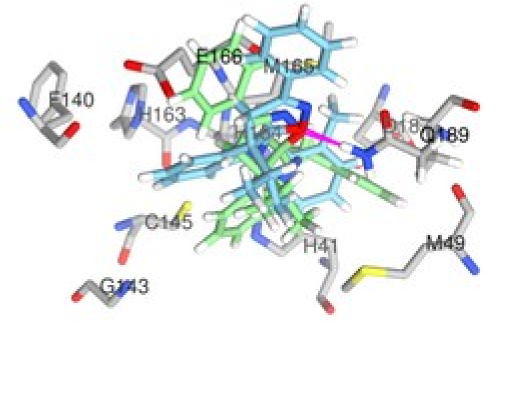	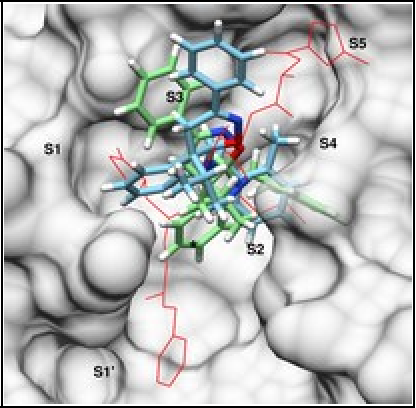
**101_RS**	-33.0	-10.2	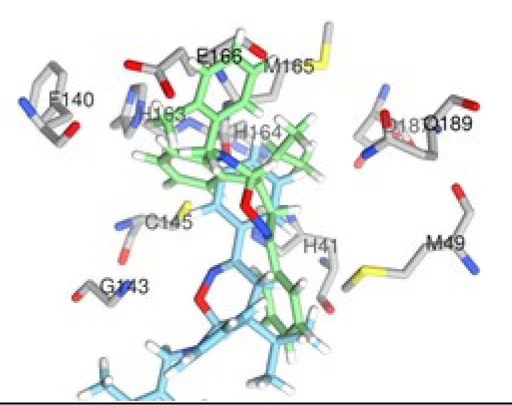	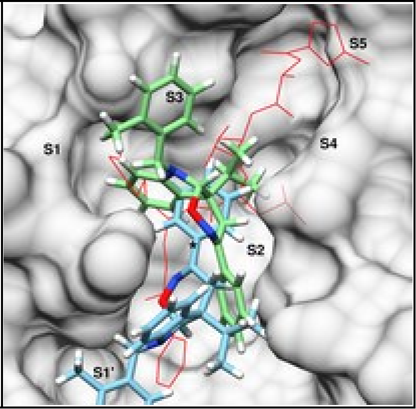

**101_SR**	-33.2	-11.1	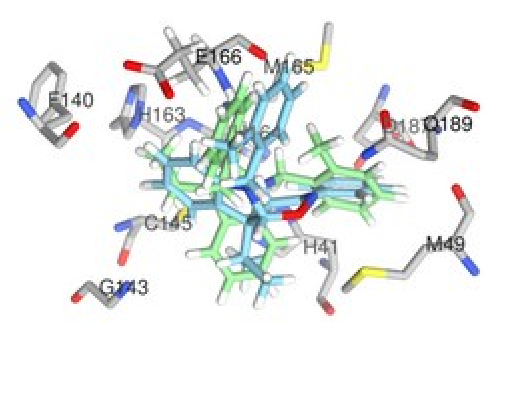	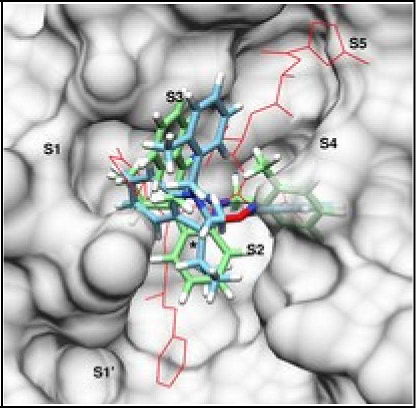
**101_SS**	-25.9	-10.8	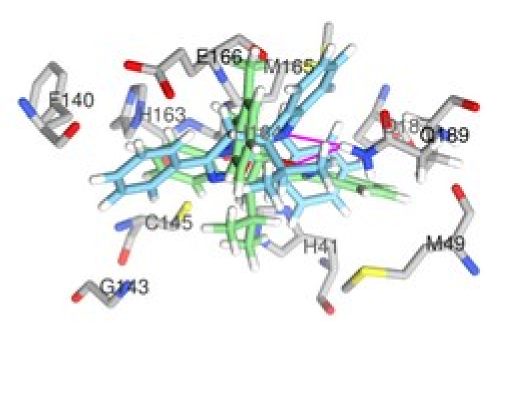	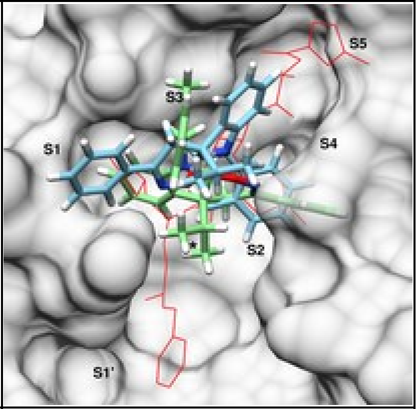
**102_SR**	-25.7	-9.7	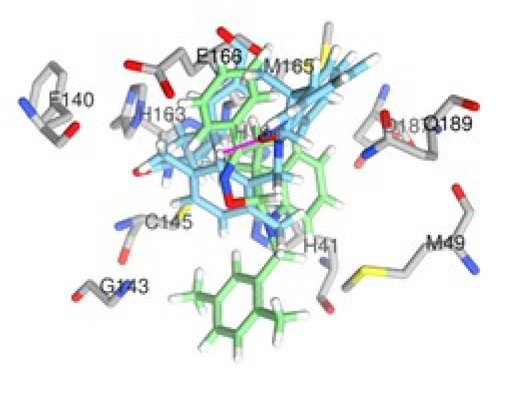	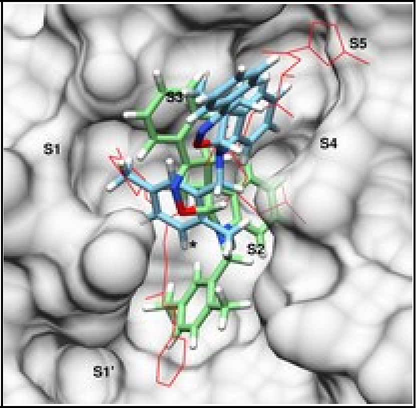
**102_SS**	-31.7	-10.7	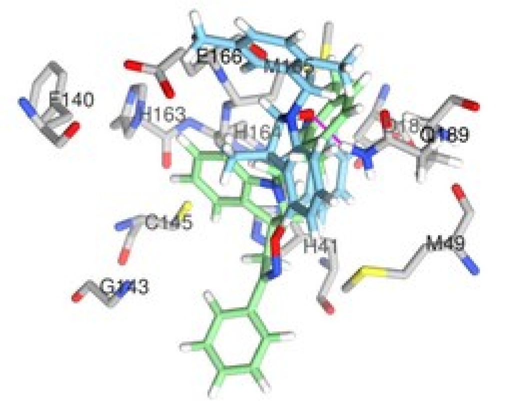	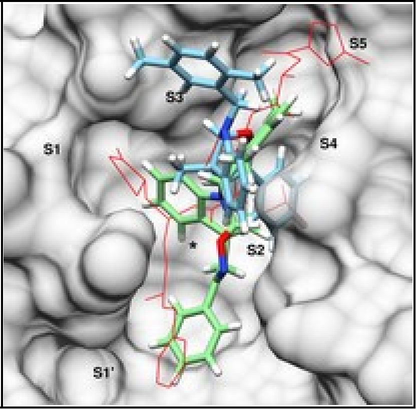

**102_RR**	-28.6	-12.5	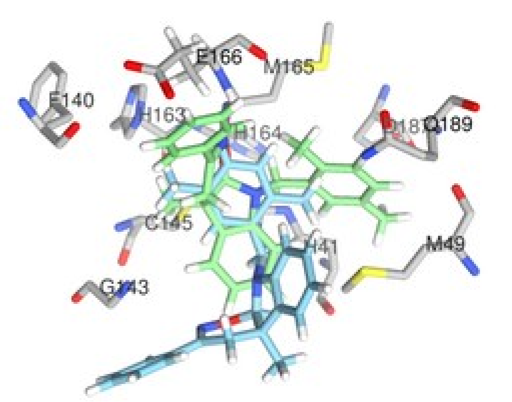	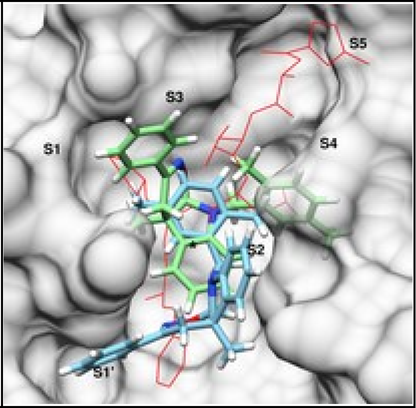
**102_RS**	-31.0	-11.5	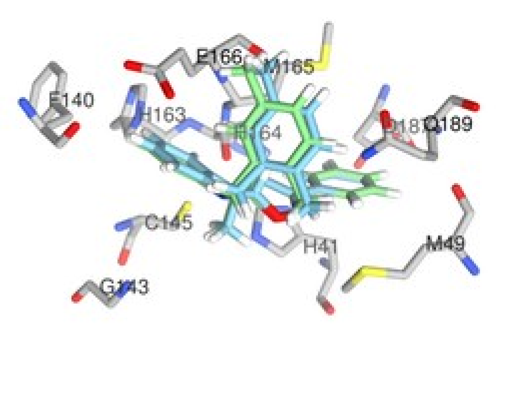	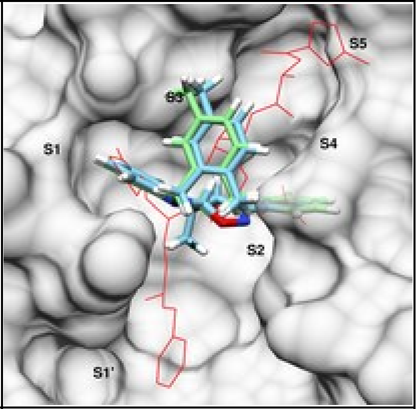
**103_RR**	-26.0	-11.0	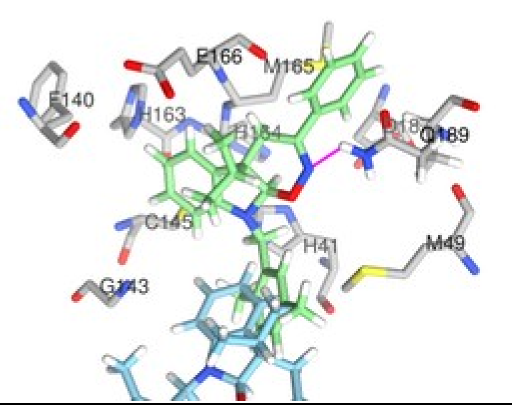	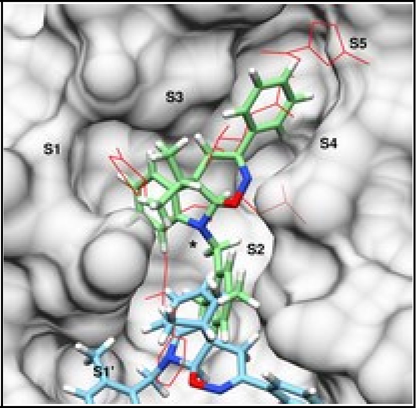
**103_RS**	-32.3	-10.4	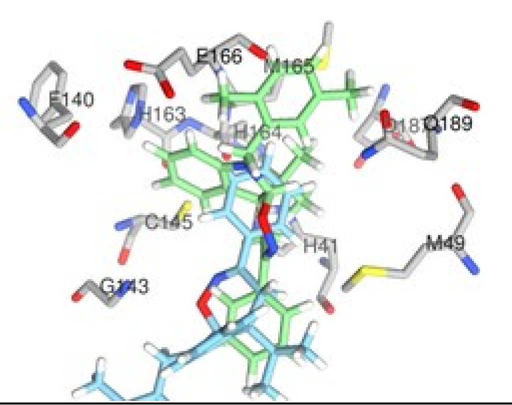	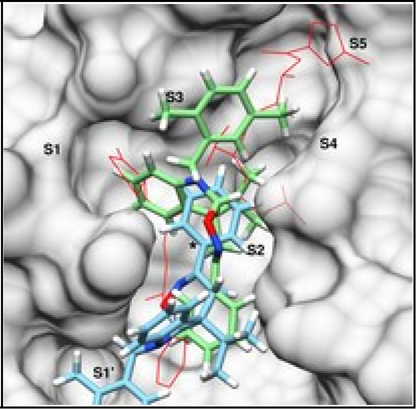

**103_SR**	-34.0	-11.1	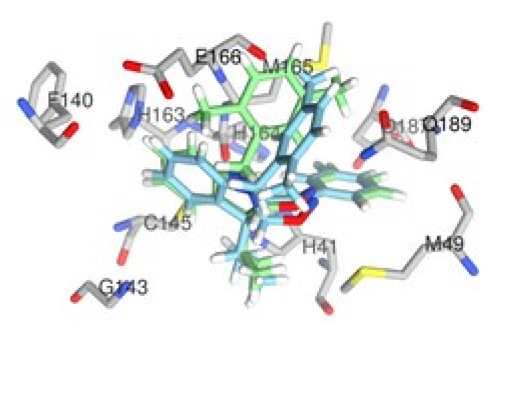	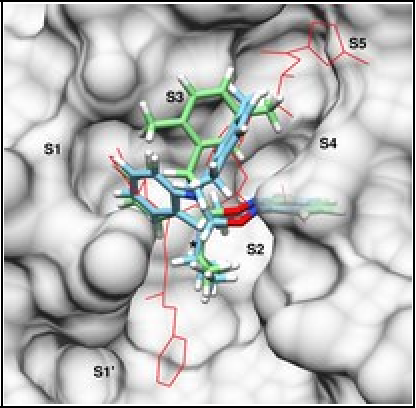
**103_SS**	-32.6	-11.7	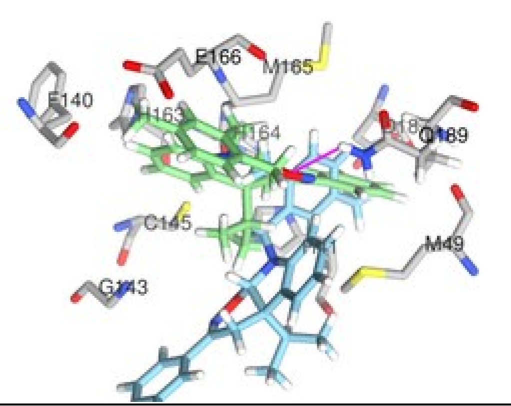	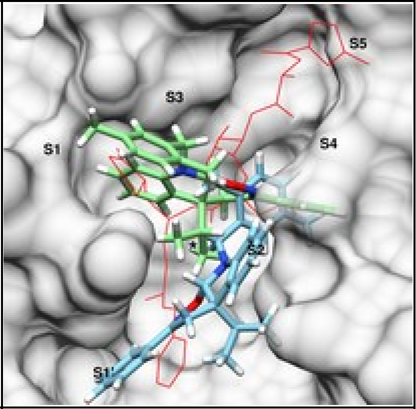
**104_RR**	-31.5	-12.3	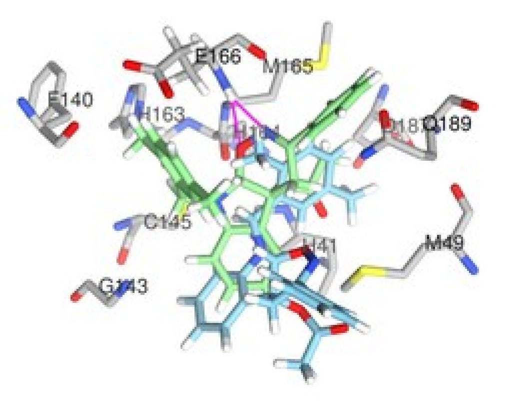	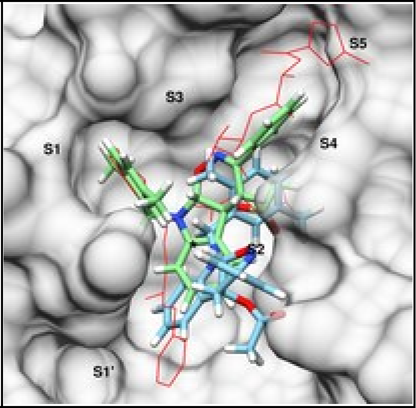
**104_RS**	-35.9	-12.1	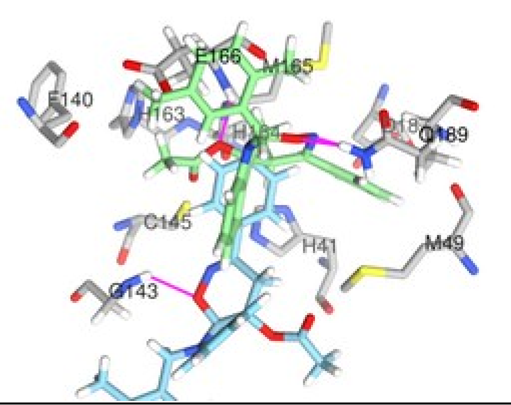	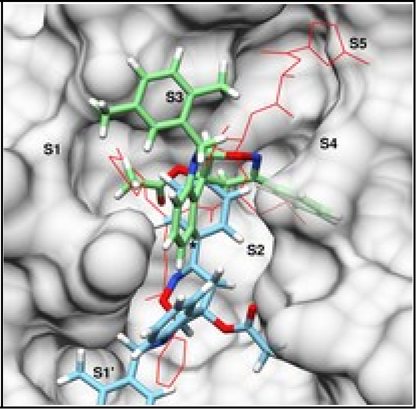

**104_SR**	-33.6	-12.7	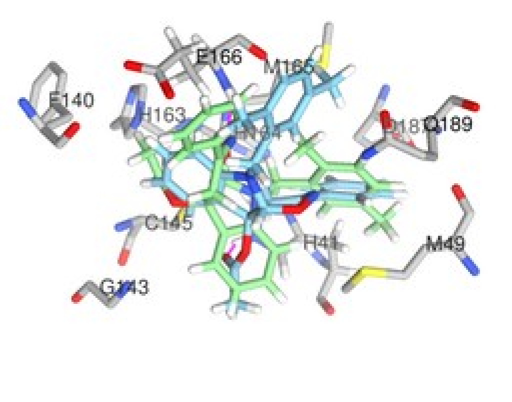	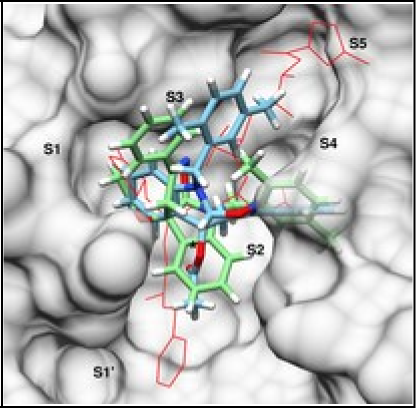
**104_SS**	-35.5	-11.0	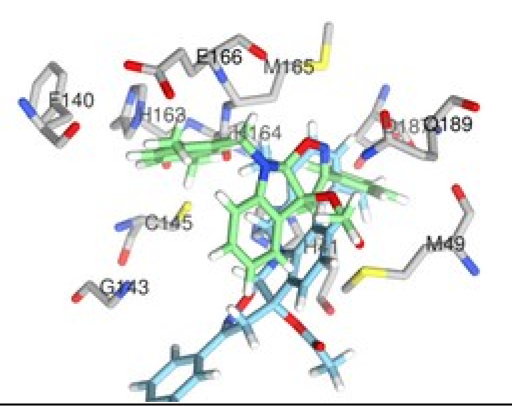	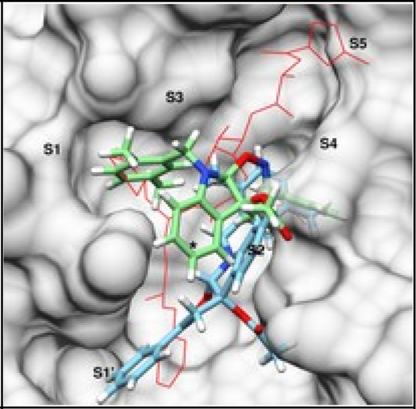
**105_SR**	-32.4	-13.1	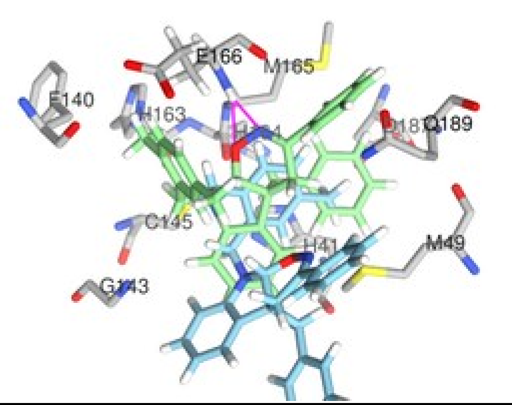	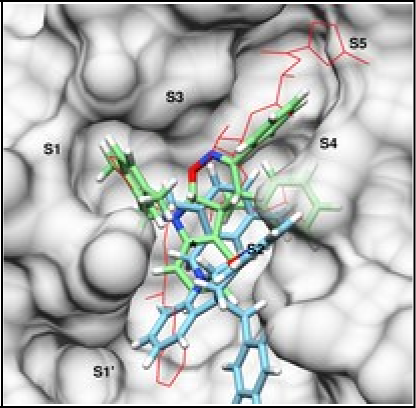
**105_SS**	-33.1	-11.8	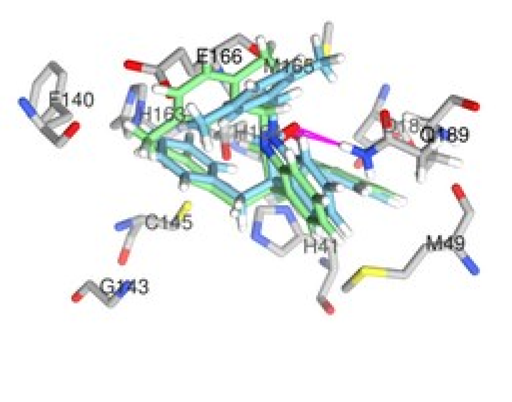	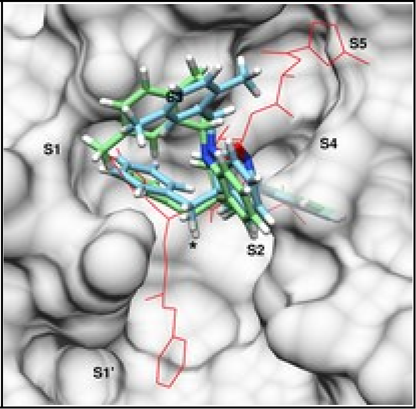

**105_RR**	-30.2	-12.8	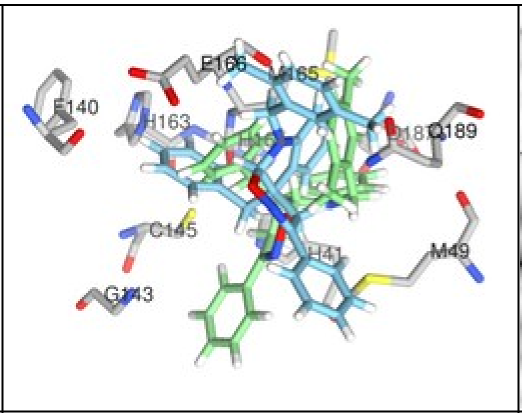	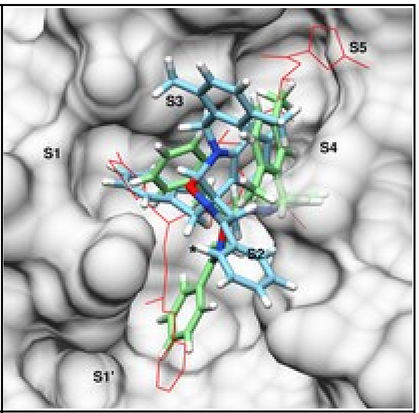
**105_RS**	-36.2	-12.3	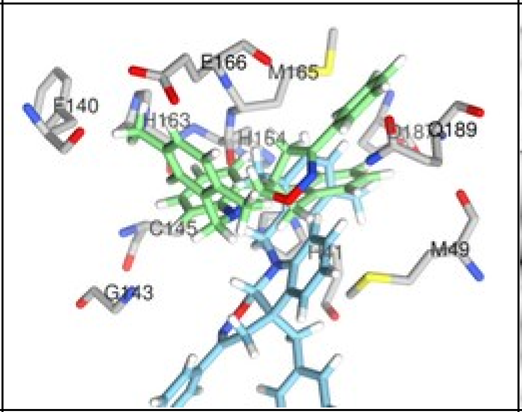	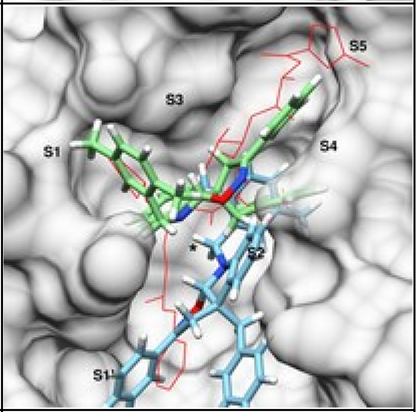
**106_RR**	-30.2	-11.7	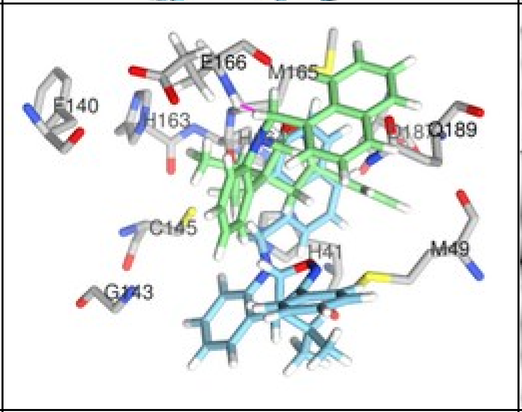	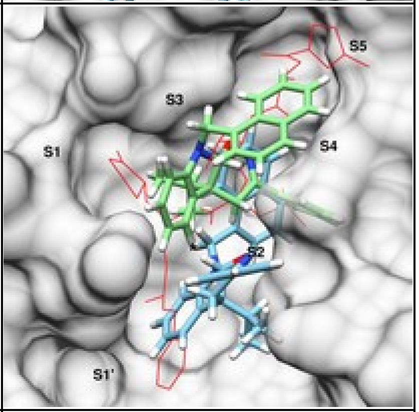
**106_RS**	-34.3	-10.6	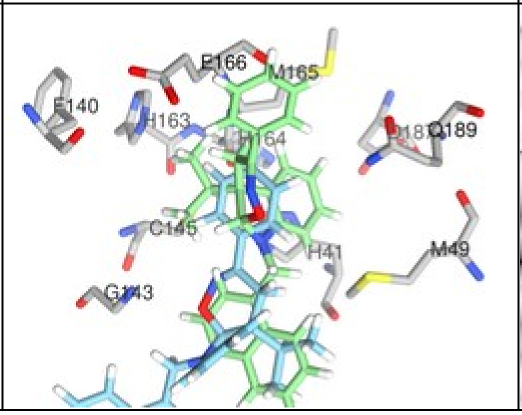	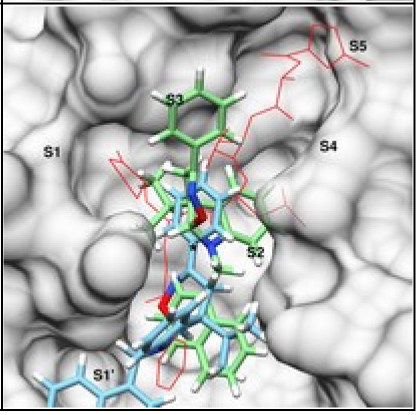

**106_SR**	-33.4	-11.3	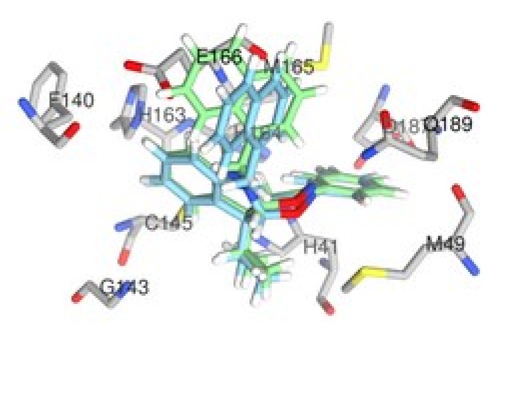	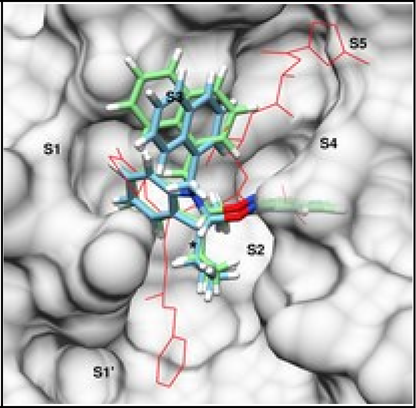
**106_SS**	-28.4	-11.8	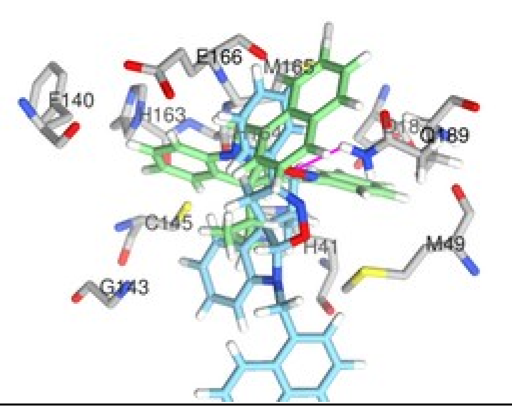	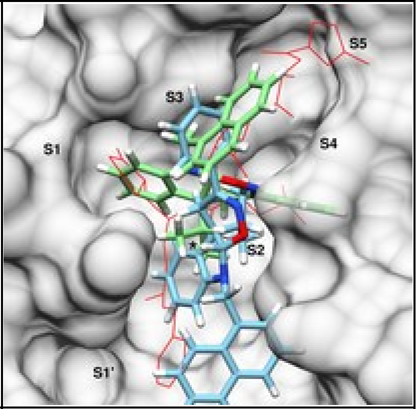
**107_SR**	-32.6	-10.0	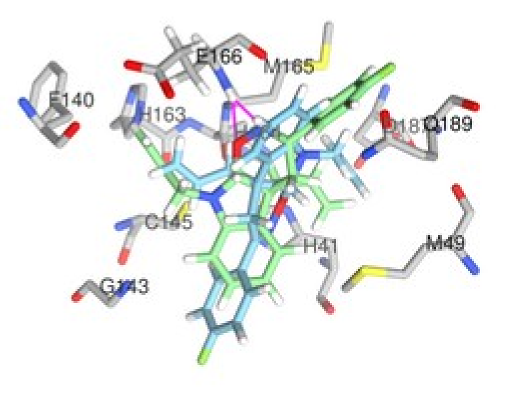	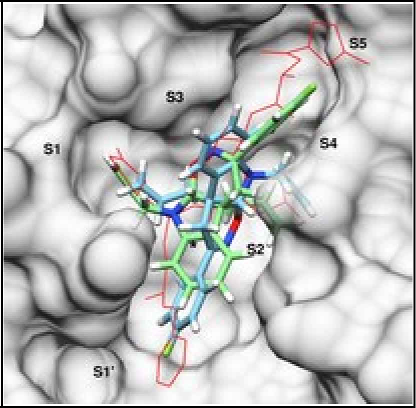
**107_SS**	-32.1	-9.8	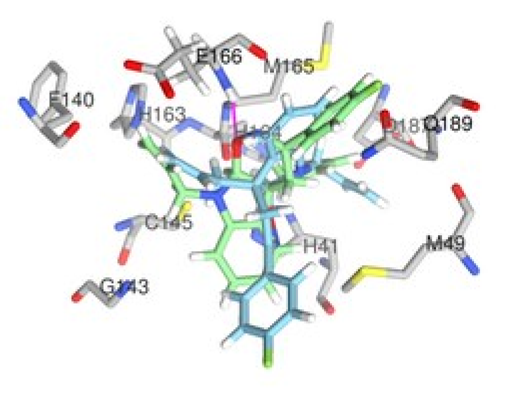	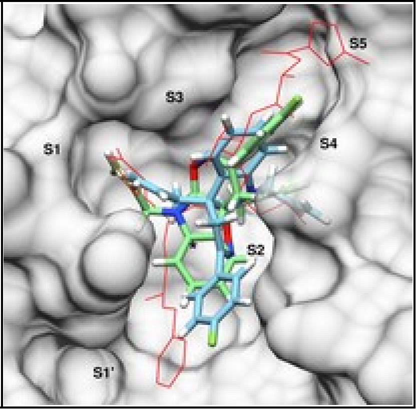

**107_RR**	-30.2	-10.2	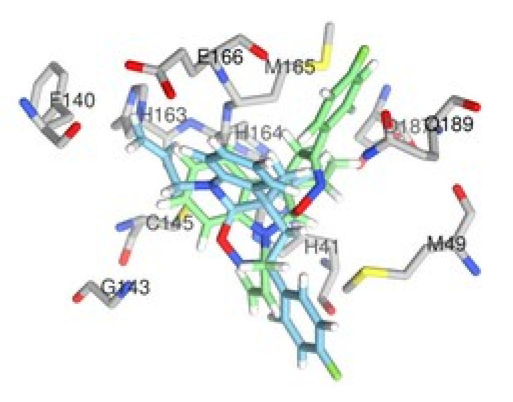	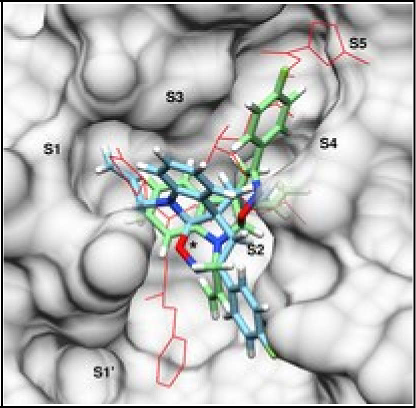
**107_RS**	-28.7	-10.4	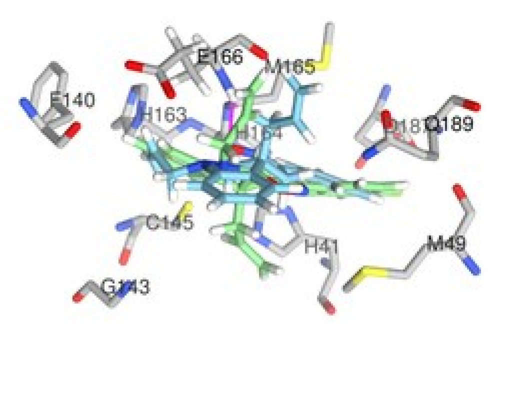	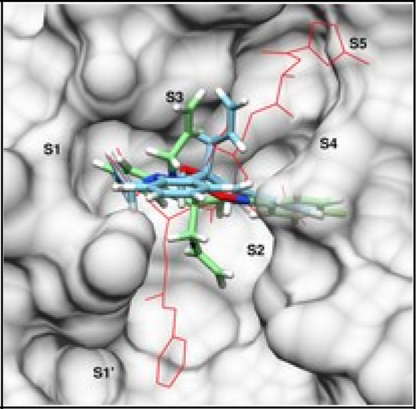
**108_SR**	-36.3	-10.6	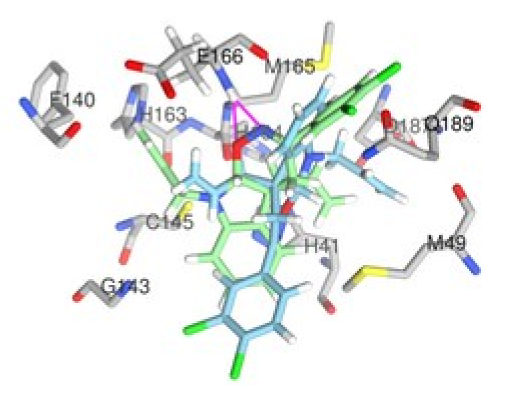	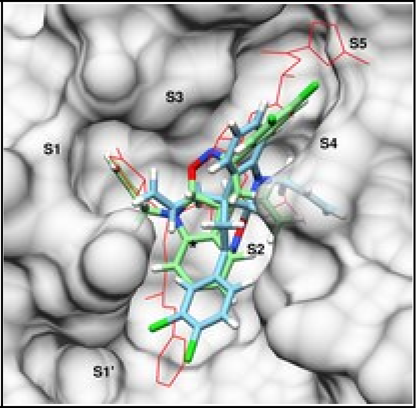
**108_SS**	-33.7	-9.8	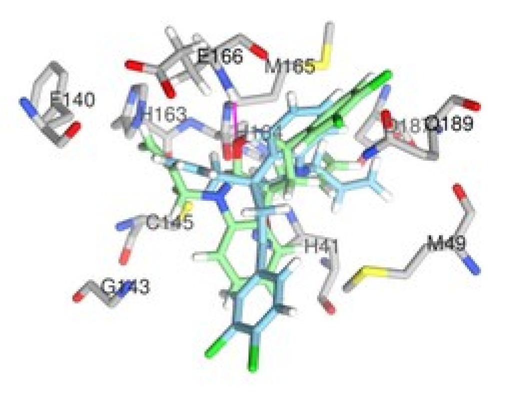	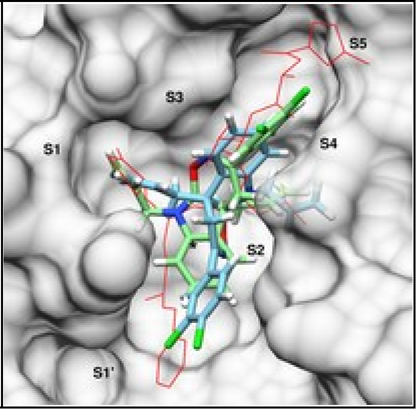

**108_RR**	-34.3	-9.9	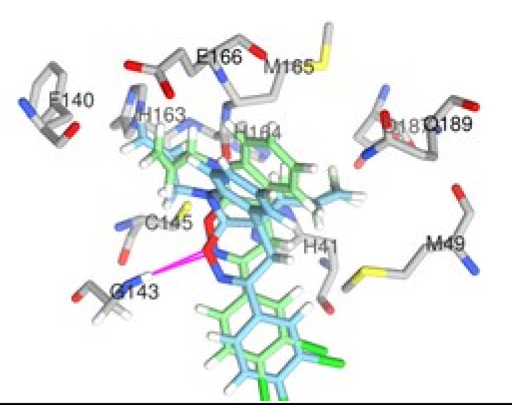	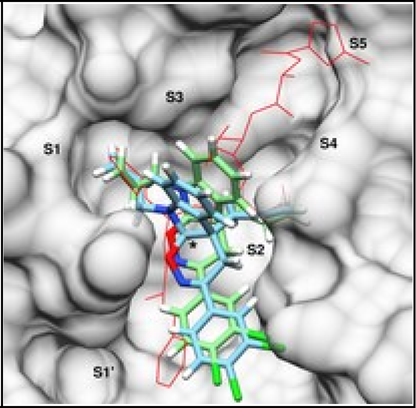
**108_RS**	-34.8	-9.3	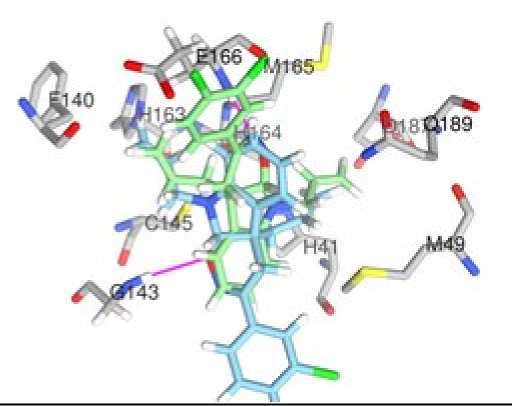	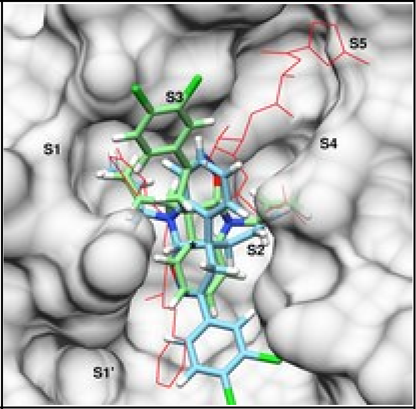
**109_SR**	-33.5	-9.1	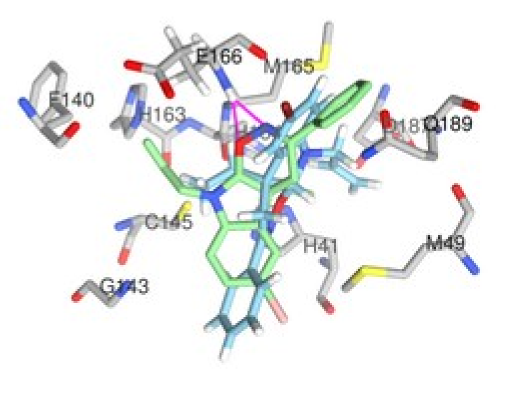	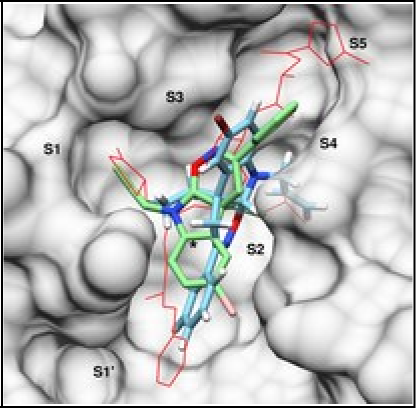
**109_SS**	-31.2	-8.9	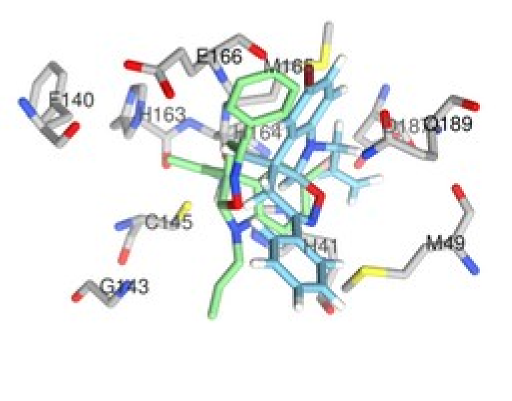	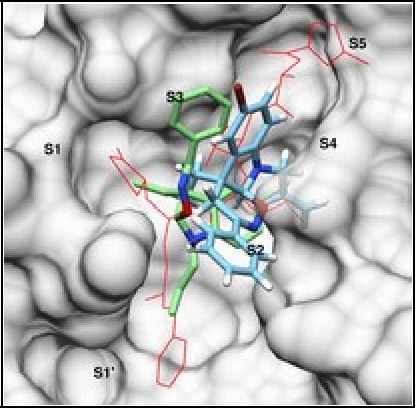

**109_RR**	-30.6	-9.1	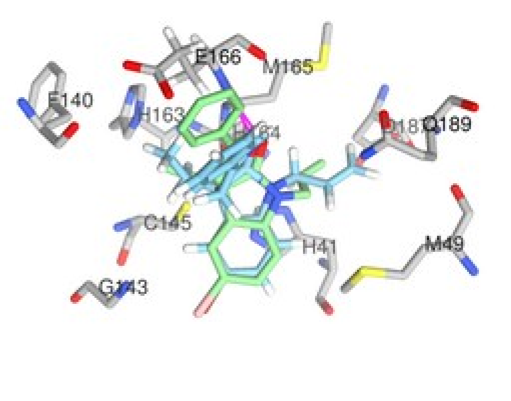	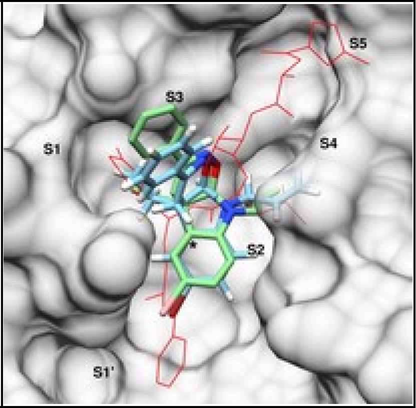
**109_RS**	-29.6	-9.1	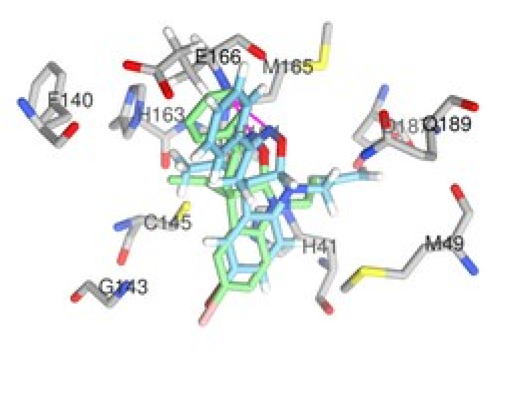	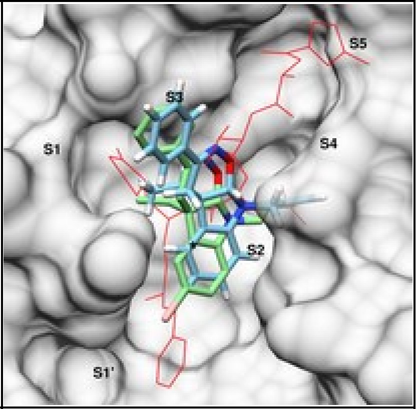
**110_SR**	-39.1	-10.4	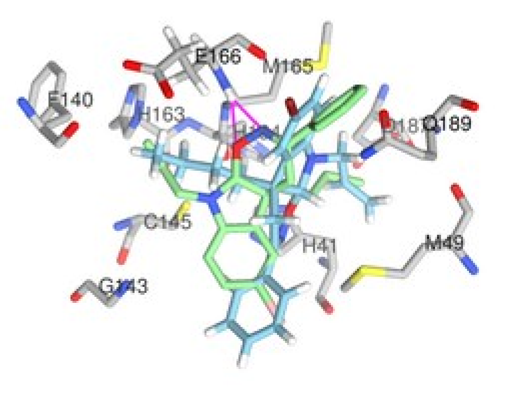	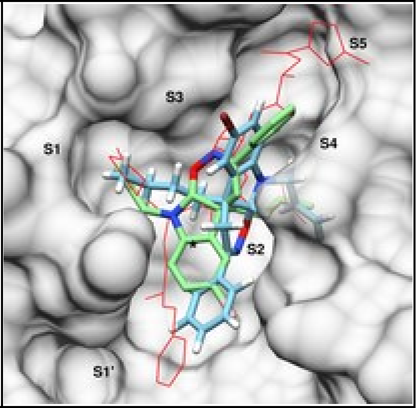
**110_SS**	-35.2	-10.1	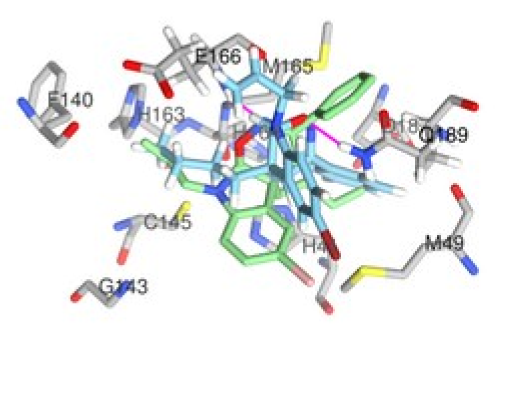	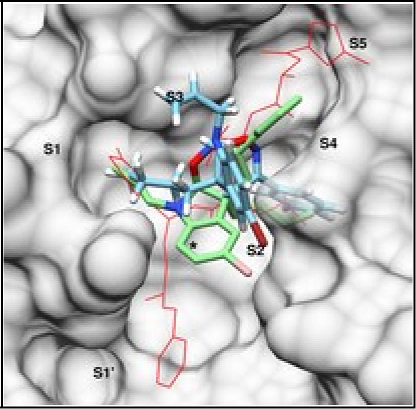

**110_RR**	-33.3	-9.6	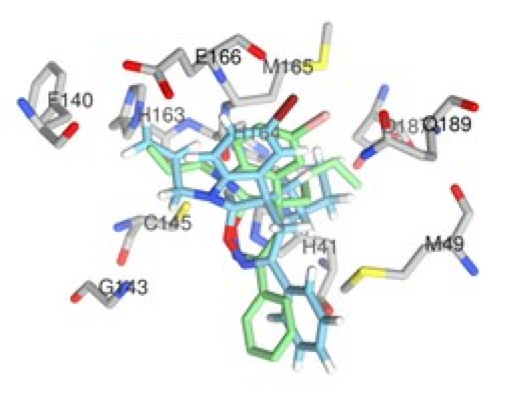	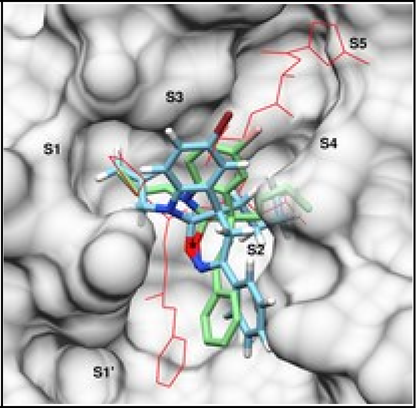
**110_RS**	-32.5	-8.9	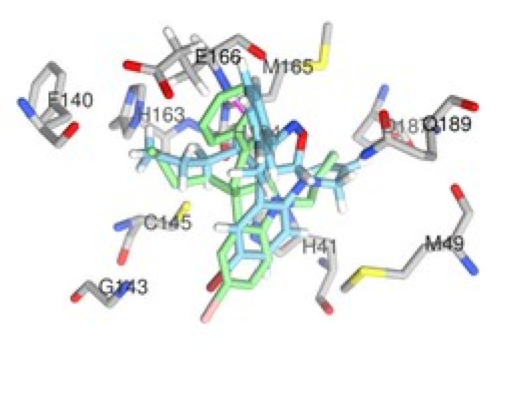	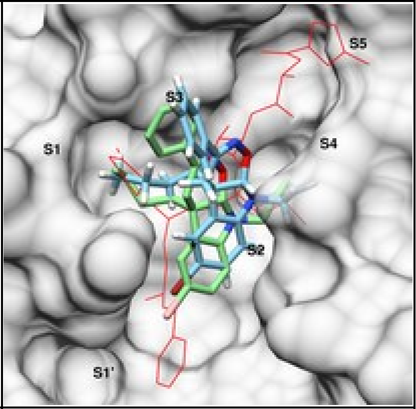
**111_SR**	-29.3	-12.4	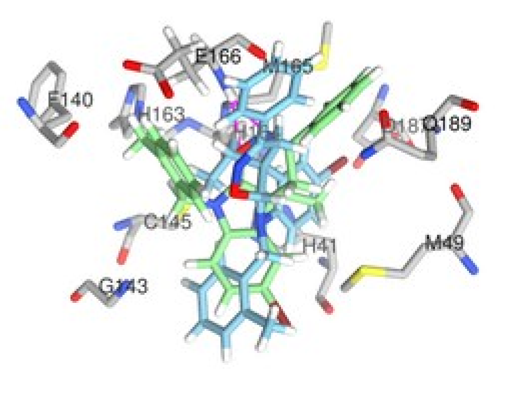	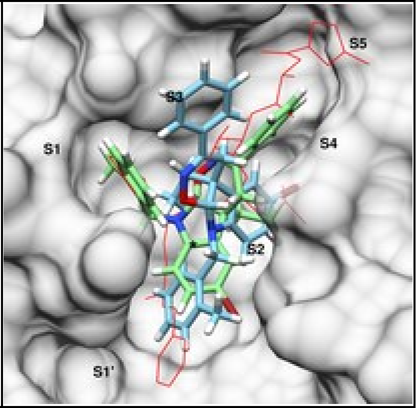
**111_SS**	-33.0	-9.9	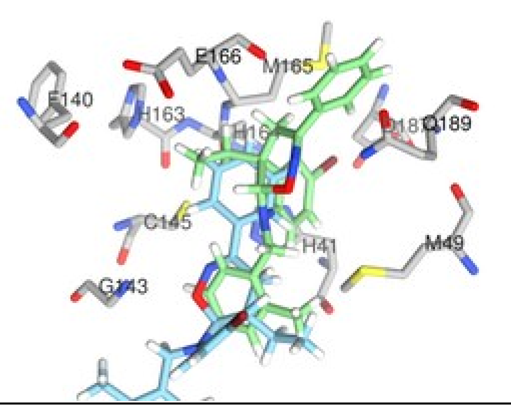	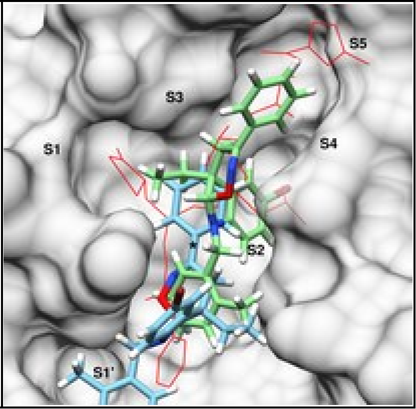

**111_RR**	-27.1	-10.7	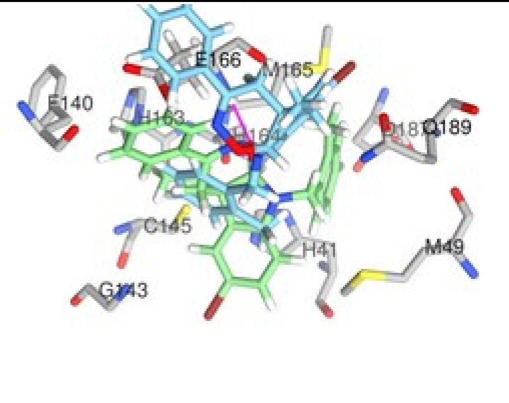	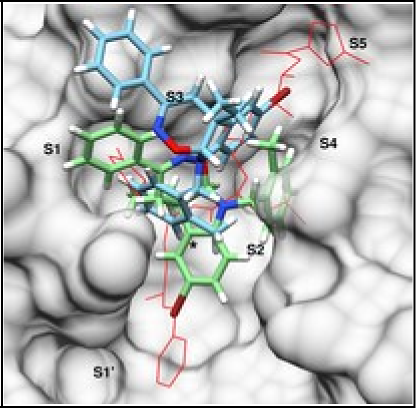
**111_RS**	-29.8	-10.2	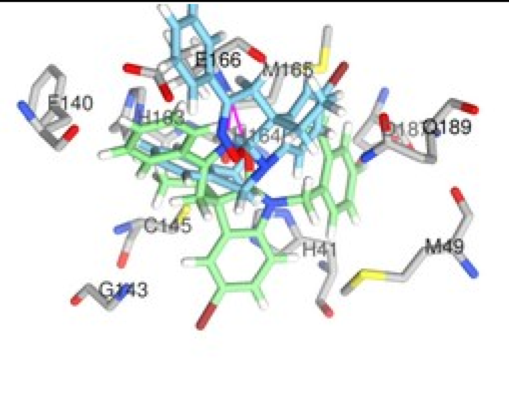	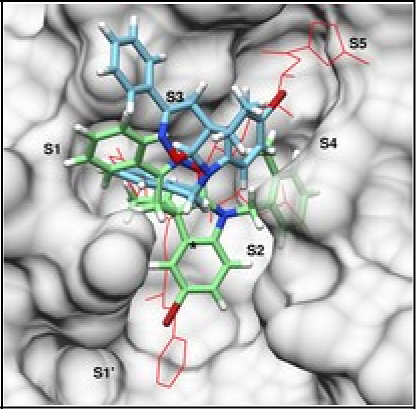
**112_SR**	-30.7	-12.4	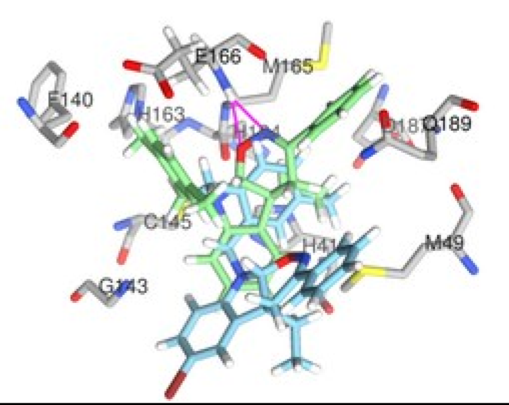	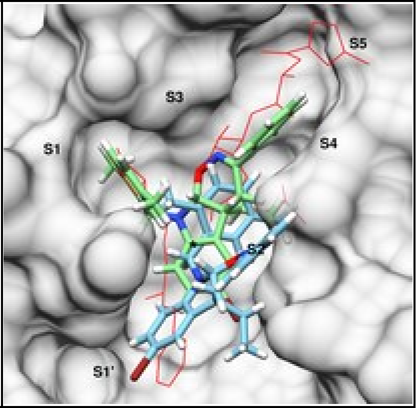
**112_SS**	-33.2	-11.4	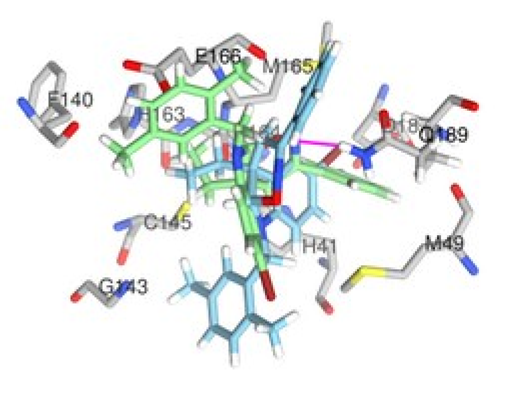	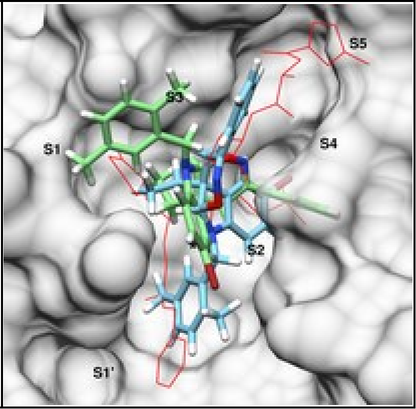

**112_RR**	-29.4	-12.1	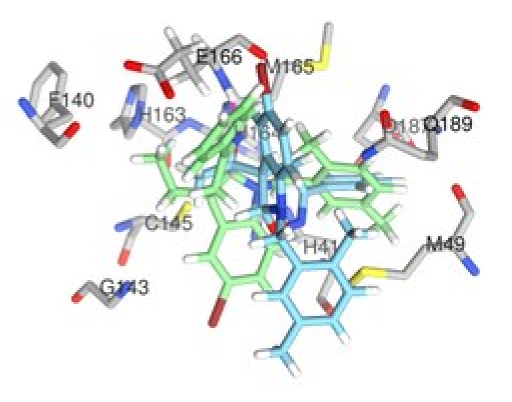	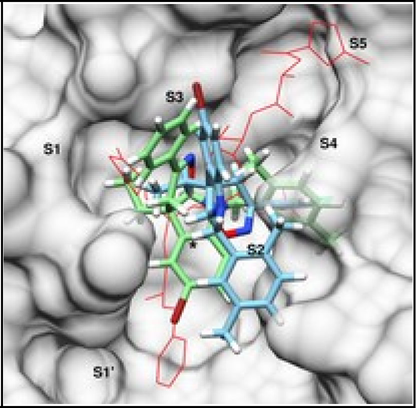
**112_RS**	-33.2	-11.7	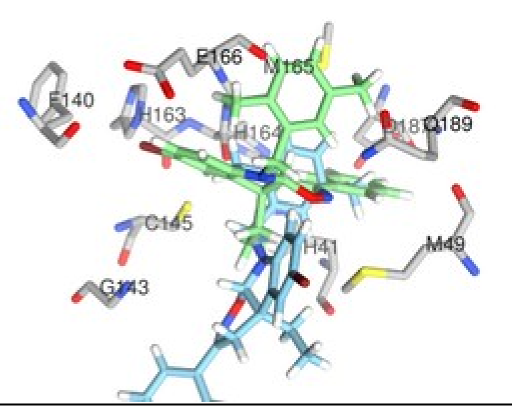	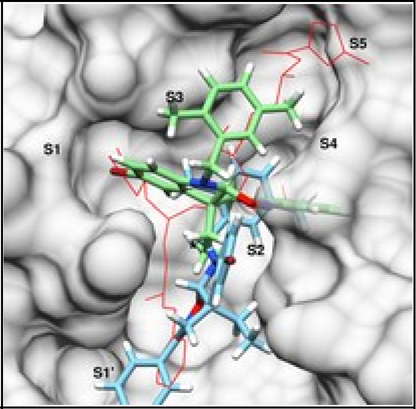
**113_SR**	-29.9	-10.8	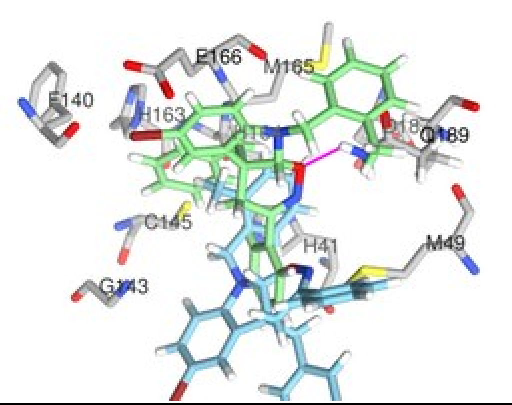	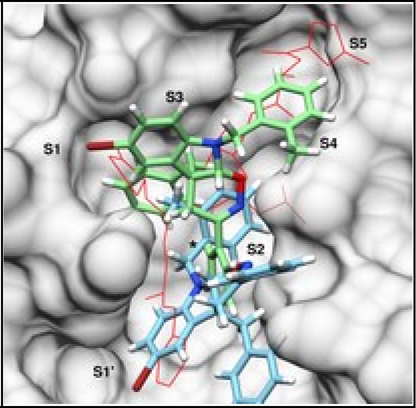
**113_SS**	-35.4	-11.3	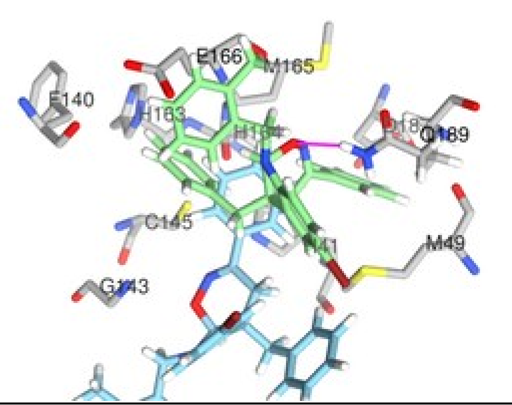	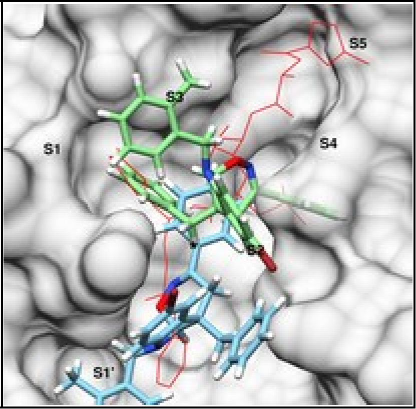

**113_RR**	-31.8	-10.7	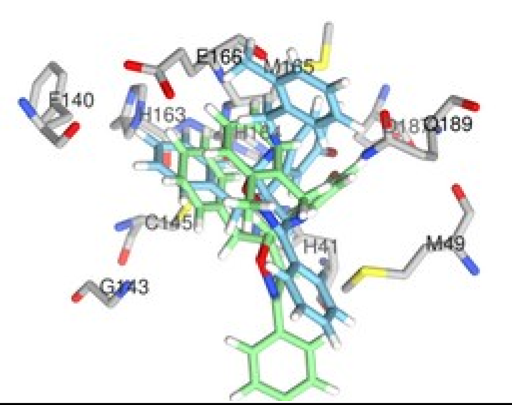	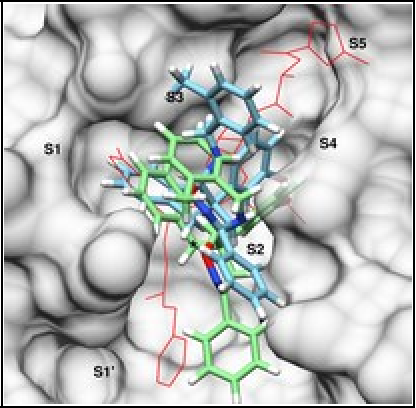
**113_RS**	-31.6	-12.5	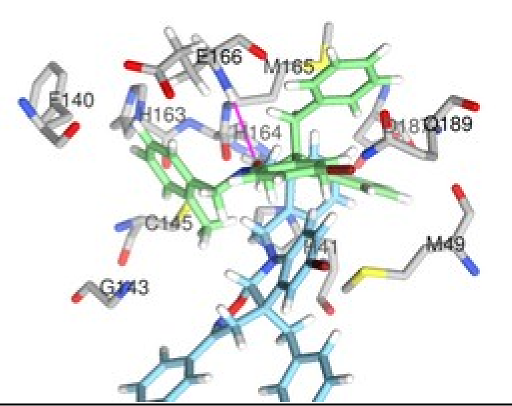	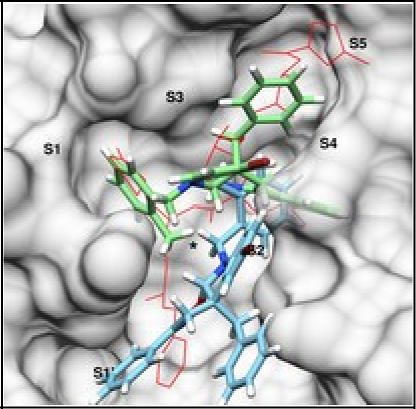
**114_RR**	-31.2	-10.4	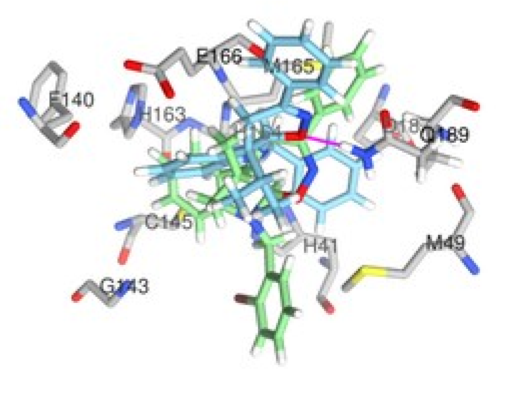	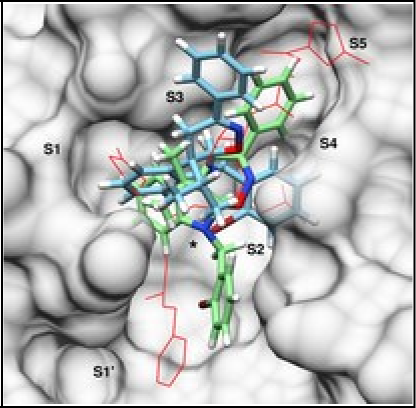
**114_RS**	-32.3	-9.9	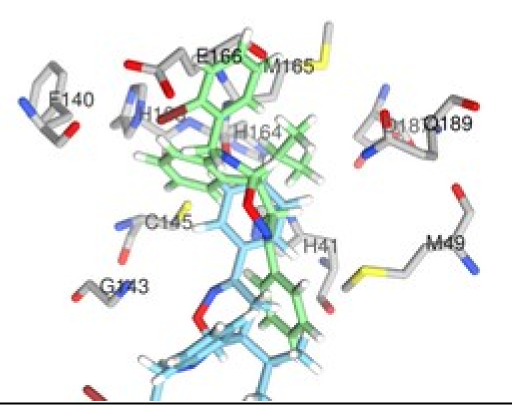	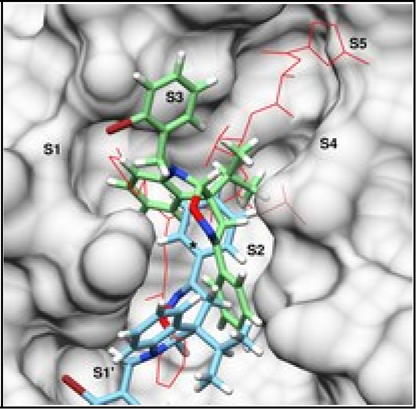

**114_SR**	-33.4	-10.5	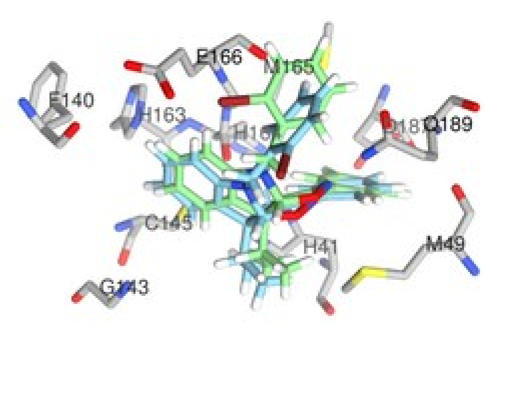	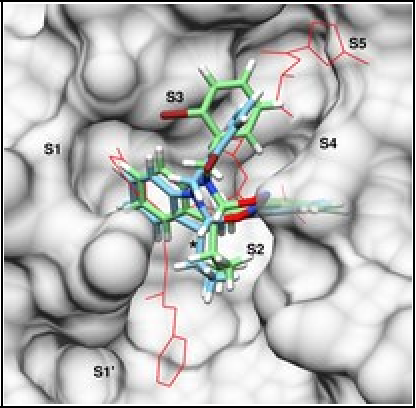
**114_SS**	-27.0	-11.7	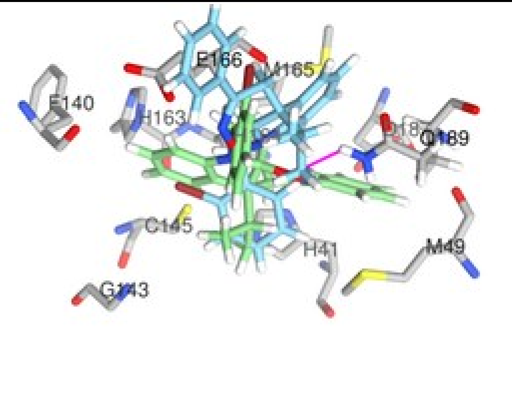	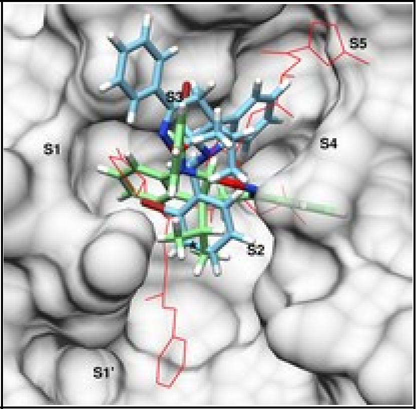
**115_SR**	-29.5	-10.2	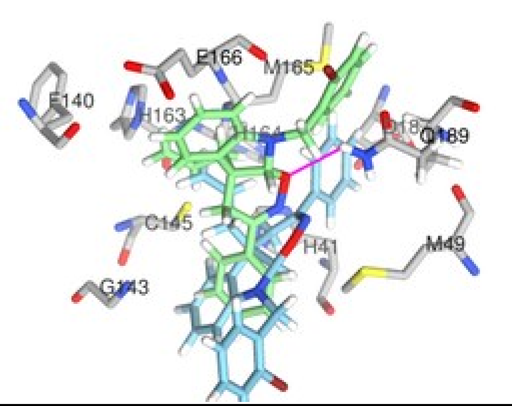	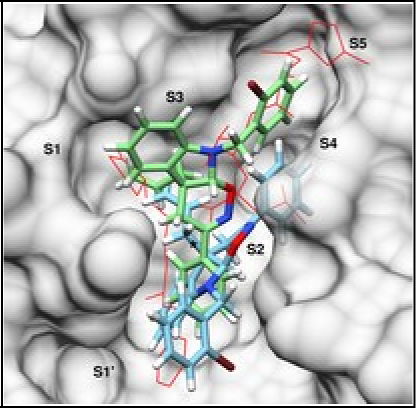
**115_SS**	-36.0	-10.0	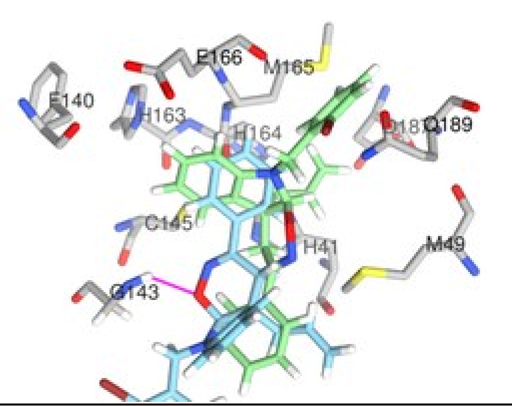	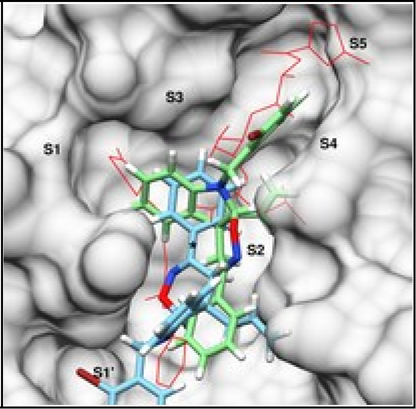

**115_RR**	-33.5	-10.5	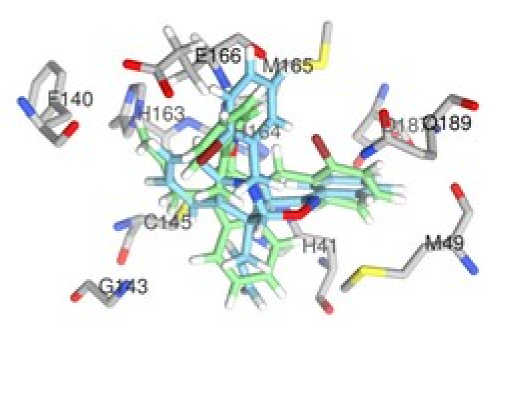	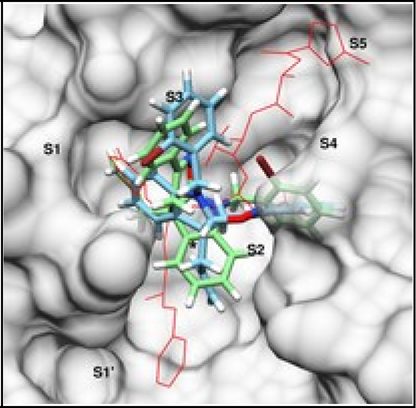
**115_RS**	-32.2	-11.1	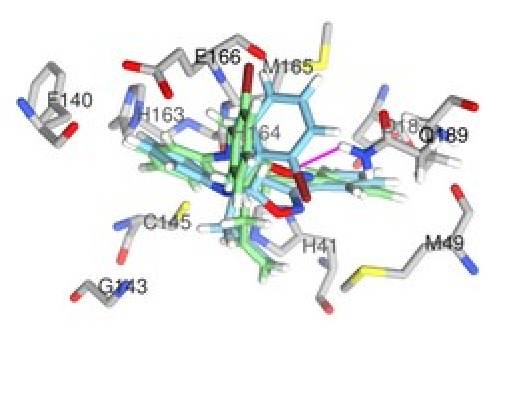	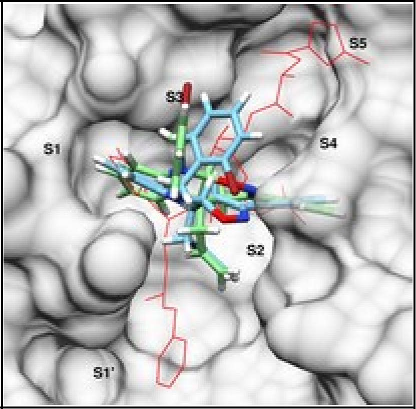
**116_RR**	-34.9	-9.8	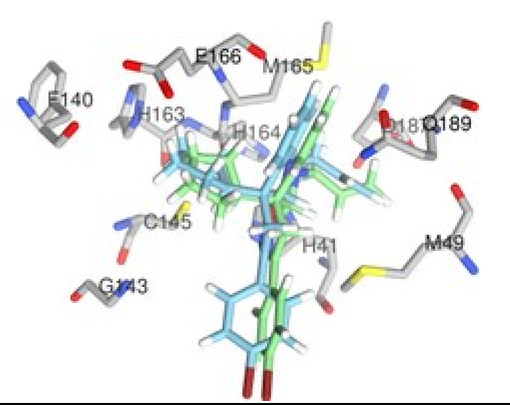	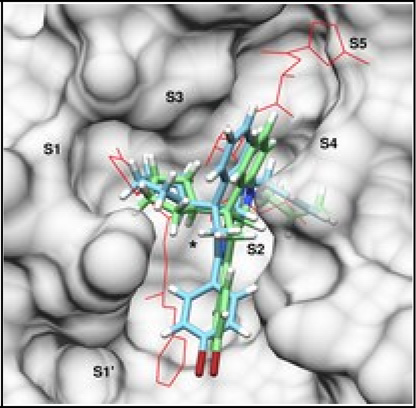
**116_RS**	-31.2	-10.8	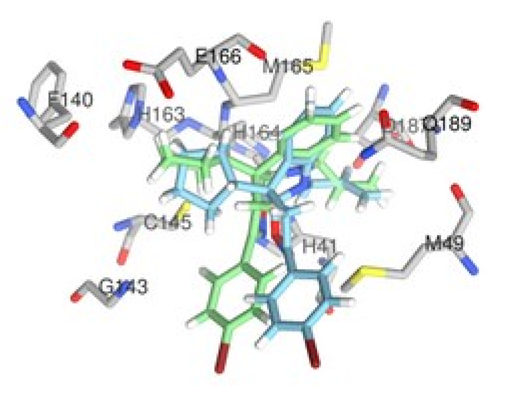	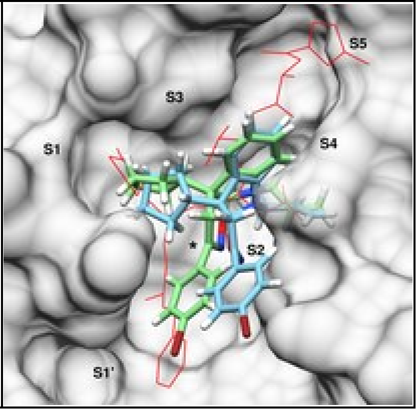

**116_SR**	-36.5	-9.9	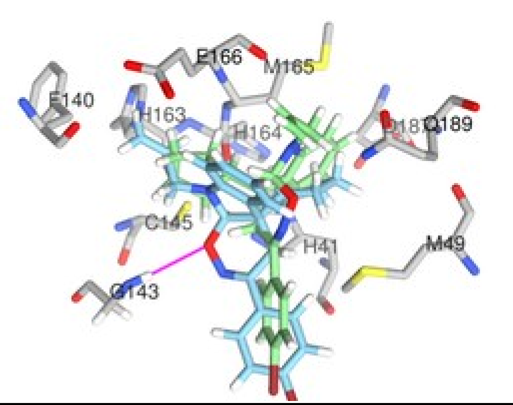	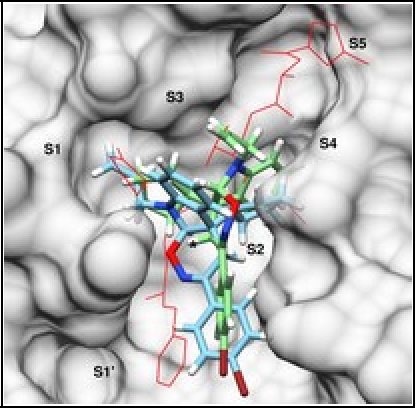
**116_SS**	-30.2	-9.9	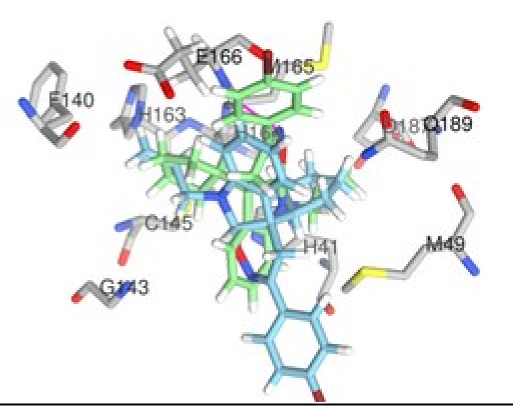	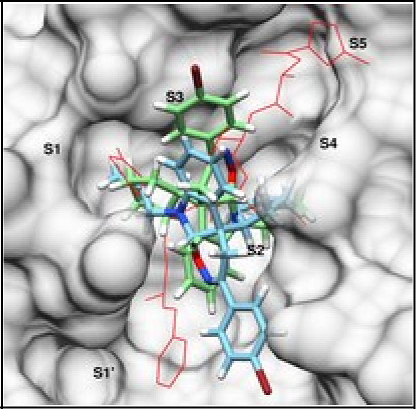
**117_SR**	-28.0	-12.3	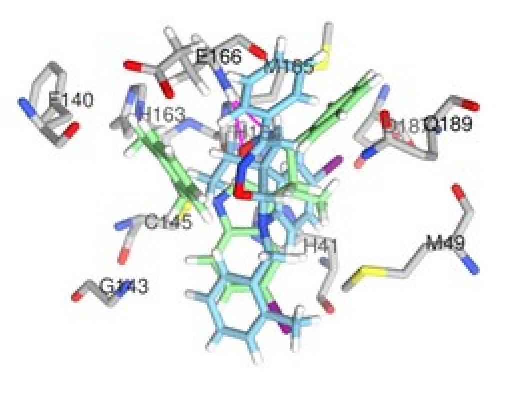	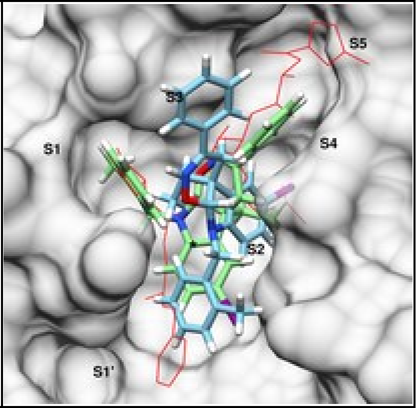
**117_SS**	-33.5	-10.3	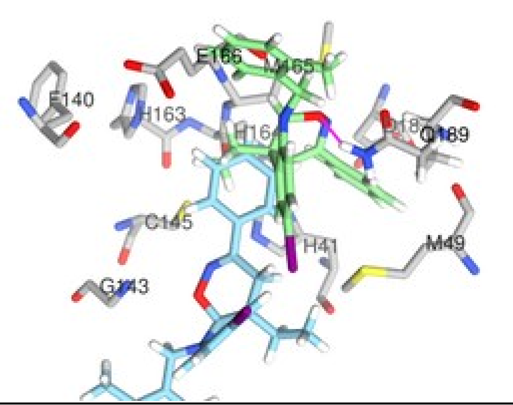	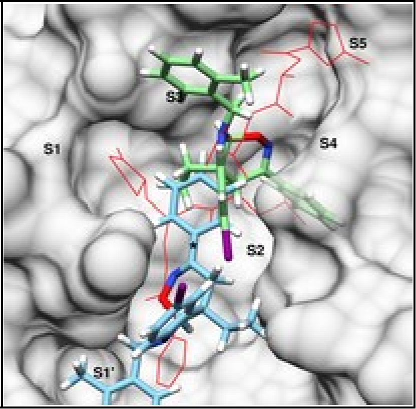

**117_RR**	-26.0	-11.6	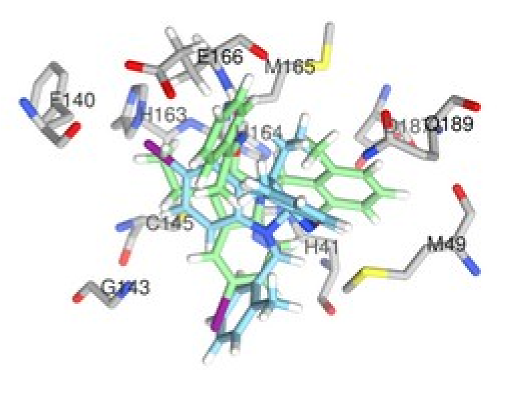	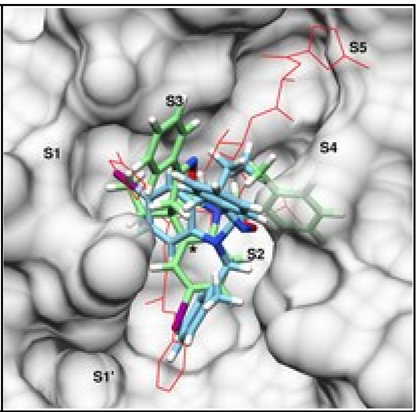
**117_RS**	-29.9	-11.6	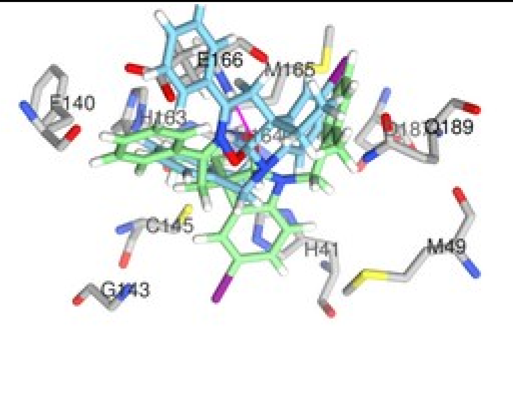	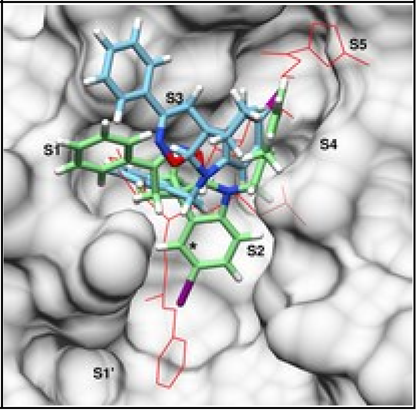
